# The effect of EMPAgliflozin on markers of inflammation in patients with concomitant type 2 diabetes mellitus and Coronary ARtery Disease: the EMPA-CARD randomized controlled trial

**DOI:** 10.1186/s13098-022-00951-5

**Published:** 2022-11-17

**Authors:** Sepehr Gohari, Tara Reshadmanesh, Hadi Khodabandehloo, Amir Karbalaee-Hasani, Hassan Ahangar, Shahram Arsang-Jang, Faramarz Ismail-Beigi, Mohsen Dadashi, Samin Ghanbari, Homa Taheri, Mojtaba Fathi, Muhammad Javad Muhammadi, Reyhaneh Mahmoodian, Atieh Asgari, Mohammadreza Tayaranian, Mehdi Moharrami, Mahsa Mahjani, Bijan Ghobadian, Hossein Chiti, Sheida Gohari

**Affiliations:** 1 Student Research Center, School of Medicine, Zanjan University of Medical Sciences, Zanjan, Iran; 2 Department of Family Medicine, Alborz University of Medical Science, Karaj, Alborz Iran; 3 Department of Clinical Biochemistry, School of Medicine, Zanjan University of Medical Sciences, Zanjan, Iran; 4 Department of Cardiology, School of Medicine, Mousavi Hospital, Zanjan University of Medical Sciences, Zanjan, Iran; 5 Department of Biostatistics, School of Medicine, Zanjan University of Medical Sciences, Zanjan, Iran; 6 Department of Medicine, Case Western Reserve University, University Hospitals Cleveland Medical Center, Cleveland, OH USA; 7 Endocrinology and Metabolism Center, Department of Internal Medicine, Imam Ali Hospital, Alborz University of Medical Sciences, Karaj, Alborz Iran; 8 General Practitioner, School of Medicine, Shahid Beheshti University of Medical Sciences, Tehran, Iran; 9 Endocrinology and Metabolism Research Centre, School of Medicine, Vali-e-Asr Hospital, Zanjan University of Medical Sciences, Zanjan, Iran; 10 Department of Systems Science and Industrial Engineering, State University of New York at Binghamton, Binghamton, NY USA

**Keywords:** SGLT2 inhibitor, Empagliflozin, Randomized controlled trial, Coronary artery disease, Inflammation, Type 2 diabetes mellitus, Oxidative stress, Platelet function, Cytokine

## Abstract

Systemic inflammation and oxidative burden in patients with type 2 diabetes mellitus (T2DM) causes deleterious cardiovascular outcomes. We sought to investigate the clinical antioxidative and anti-inflammatory effects of empagliflozin. Platelet function, oxidant and antioxidant biomarkers and pro-inflammatory agents at baseline and at 26 weeks were measured. A total of 95 patients (41.05% male, mean age 62.85 ± 7.91 years, mean HbA_1c_ 7.89 ± 0.96%) with concomitant T2DM and coronary artery disease (CAD) were randomized (1:1) to receive empagliflozin (10 mg/daily) or placebo. Patients treated with empagliflozin had lower levels of interleukin 6 (IL-6) (adjusted difference (adiff): − 1.06 pg/mL, 95% CI − 1.80; − 0.32, P = 0.006), interleukin 1*β* (IL-1*β*) and high-sensitive C-reactive protein (Hs-CRP) (adiff: − 4.58 pg/mL and − 2.86 mg/L; P = 0.32 and 0.003, respectively) compared to placebo. There were elevations in super oxidase dismutase (SOD) activity, glutathione (GSHr), and total antioxidant capacity (TAC) with empagliflozin (adiff: 3.7 U/mL, 0.57 muM, and 124.08 mmol/L, 95% CI 1.36; 6.05, 0.19; 0.95, and 47.98; 200.18, P = 0.002, 0.004, and 0.002, respectively). While reactive oxygen species (ROS) improved significantly (adiff: − 342.51, 95% CI − 474.23; − 210.79, P < 0.001), the changes in catalase activity (CAT), malondialdehyde (MDA), or protein carbonyl groups (PCG) were not significant. Moreover, the P-selectin antigen expression on platelet surface was significantly reduced (adiff: − 8.81, 95% CI − 14.87; − 2.75, P = 0.005). Markers of glycemic status (fasting blood glucose, HbA_1c_, and HOMA-IR (homeostatic model assessment for insulin resistance) significantly improved (P < 0.001). Among patients with T2DM and CAD, 6-month treatment with empagliflozin can mitigate inflammation, platelet activity and oxidative stress and is associated with clinical cardiovascular benefits.

*Trial Registration* Iranian Registry of Clinical Trials. www.IRCT.ir, Identifier: IRCT20190412043247N2. Registration Date: 6/13/2020. Registration timing: prospective

**Supplementary Information:**

The online version contains supplementary material available at 10.1186/s13098-022-00951-5.

## Introduction

Type 2 diabetes mellitus (T2DM) is associated with cardiovascular diseases and is a leading cause of mortality in diabetic patients [1]. Oxidative stress generates through exposure to reactive oxygen species (ROS) and is known to be associated with the development of T2DM [2]. Hyperglycemia determines overproduction of ROS by the mitochondrial electron transport chain leading to tissue damages and elevated apoptosis. Oxidative stress plays an important role in the development of cardiac hypertrophy and remodeling. The role of oxidative stress and sustained inflammation in the pathogenesis of diabetic atherosclerosis have been pronounced recently, since the chronic increase of ROS precipitates its development [3]. Atherosclerosis is a complex process that entails inflammatory pathways and promotes the production of proinflammatory cytokines such as interleukin 6 (IL-6) [4].

Empagliflozin is a highly selective sodium glucose co-transporter-2 inhibitor (SGLT2-i) which was primarily introduced as a glucose-lowering agent for the treatment of T2DM. A growing body of evidence have identified the antioxidant properties of empagliflozin [5]. In vivo experiments have shown that empagliflozin plays a cardioprotective role by impeding inflammatory responses and myocardial fibrosis in human hearts, and therefore, prevents the progression of diabetic cardiomyopathy [6].

The EMPA-CARD trial aimed to investigate the potential impact of empagliflozin on inflammatory profile in patients with concomitant T2DM and coronary artery disease (CAD).

## Materials and methods

### Clinical methods

This manuscript is written in accordance with the SAMPL and CONSORT guidelines.

#### Trial design

The study protocol has been published in details previously [7]. In summary, this was a multicenter double blind randomized placebo-controlled trial (RCT) that investigated the effects of empagliflozin 10 mg once daily versus placebo in addition to standard anti-diabetic treatment on inflammatory status in patients with concomitant T2DM and CAD. This study was conducted in accordance with the principles of the declaration of Helsinki and all subsequent revisions. Our study was approved by the ethics committee of Zanjan university of Medical Sciences, and the protocol was prospectively registered on the Iranian Registry of Clinical Trials (www.IRCT.ir, Identifier: IRCT20190412043247N2). A written informed consent was obtained prior to the study recruitment from all patients.

#### Participants and eligibility assessments

The included patients aged from 45 to 75 with T2DM and had documented CAD confirmed by coronary angiography (CAG) plus they had been treated with standard anti-ischemic and anti-diabetic medications for at least 3 months prior to this study. The eligible HbA_1C_ prior to recruitment for inclusion was between 6.5 to 9.5%. The key exclusion criteria were: history of SGLT2-i medications as well as any allergic reaction to SGLT2-i drugs and the use of anti-coagulants or anti-platelet drugs (except aspirin 80 mg/daily), pioglitazone, antioxidant supplements, alcohol and anti-inflammatory drugs for at least 3 months prior to enrollment; heart failure (New York Heart Association class III-IV) or left ventricular ejection fraction (LVEF) < 40%; body mass index (BMI) ≥ 40 kg/m^2^; history of transient ischemic attack (TIA), acute coronary syndrome (ACS), cerebrovascular accident (CVA), coronary artery bypass graft (CABG) surgery or percutaneous coronary intervention (PCI) during past 3 month; eGFR ≤ 45; history of recent infection (1 month prior to blood sampling); presence of pregnancy or lactation; history of diabetic ketoacidosis (DKA) or malignancy; anemia (hemoglobin < 10 g/dl) or thrombocytopenia (Platelet count < 100,000/µl).

The study recruitment phase began on June 29, 2020 and ended on March 13, 2021. Patients were recruited at 4 clinical centers (Mousavi hospital [coronary angiography registry], Vali-e-Asr hospital, and two cardiology clinics of Zanjan University of Medical Sciences) in Zanjan, Iran. Before recruitment, the registered records of patients were screened to assess their eligibility based on the inclusion/exclusion criteria. Further, they were interviewed by the investigators to be recruited to the study.

#### Intervention, randomization and follow up

At the day of recruitment (baseline), patients went through a series of evaluations that were comprised of blood samplings, directed interviews by the filling checklists that incorporated demographic information, food frequency questionnaire (FFQ), physical activity questionnaire using international physical activity questionnaire (IPAQ), 2-dimentional transthoracic echocardiography (2D-TTE), electrocardiography (ECG), and physical examination. Further patients were randomized into 2 groups: 10 mg of empagliflozin and placebo. The randomization process was employed according to the stratified randomization method. Stratification of patients was based on age, gender and HbA_1c_. With the help of Winpepi software (V 11.6), the randomization sequence was created and by the sealed envelope method, the allocation sequence was concealed until the day of recruitment. Patients were followed up every 4 weeks through phone calls. At the end of weeks 2, 12, and 26, patients attended clinic visits for safety assessments, physical examination, and thorough checkups. At each visit, patients were advised to maintain a fixed physical activity throughout their participation in the trial. All patients’ drug containers were collected at the end of week 26.

#### Outcomes

The primary outcome was changes in levels of plasma IL-6 after 26 weeks between the two groups. Secondary outcomes were: (1) changes in levels of other inflammatory biomarkers [High-sensitivity C-reactive Protein (Hs-CRP) and Interleukin 1-beta (IL-1*β*)], (2) changes oxidative stress markers in plasma [superoxide dismutase (SOD) enzyme activity, reduced glutathione (GSHr), catalase (CAT) enzyme activity, ROS, total antioxidant capacity (TAC), malondialdehyde (MDA), and protein carbonyl groups (PCG)], (3) platelet function alternations (P-selectin Ag expression intensity), and (4) glycemic status [fasting blood sugar (FBS), HbA_1c_, and homeostatic model assessment for insulin resistance (HOMA-IR)].

All the secondary outcomes were assessed by the changes from baseline to week 26 of treatment between each study arms.

#### Blinding and study monitoring

Empagliflozin and placebo tablets were given to participants with the same color and shape. Following enrollment, a code was assigned to each individual patient. Patients, researchers and healthcare providers who collected information, assessed the outcomes and analyzed the final data were all blinded to the assigned treatment (Empagliflozin or placebo), classified into groups of A and B. Data source verification in addition to study monitoring were instituted by the Research Council of Zanjan University of Medical Sciences, Iran, independent from the sponsors or any conflicting interests. Adverse event, regardless of their relevance, were all documented in the standard adverse events form and were either reported by the patients or were identified by our team of investigators throughout the study follow-up (until week 28).

### Experimental methods

#### Materials and instruments

Elisa kits used for measuring cytokines included Human IL-6 Elisa Kit (Diaclone, France) and Human IL-1*β* Elisa Kit (Zellbio, Germany). Laboratory kits used for measuring the oxidative stress biomarkers (except ROS) included SOD Activity Kit (KSOD96), CAT Activity Kit (KCAT96), Thiol Content Assay Kit (KTHI96), Total Antioxidant Assay Kit (KTAC96), Lipid Peroxidation Kit (KMDA96), and Protein Carbonyl Assay Kit (KPCA96) were purchased from KIAZIST Company, Iran. 2'-7'dichlorofluorescin diacetate (H2DCFDA) (D399, Invitrogen, USA) was used to assess Cellular ROS. Platelet agonist used to activate platelet was thrombin receptor activator peptide (TRAP-6 (SFLLRN), 4,031,274.0005; Bachem, Germany) and antibodies used for flow cytometric measurement of platelet function were FITC Mouse Anti-Human CD62P (Clone AK-4) (555,523, BD Pharmingen, USA) and FITC Mouse IgG1, κ Isotype Control (Clone MOPC-21) (555,748, BD Pharmingen, USA). The instruments used for the laboratory measurements were as follow: 3-30 K Centrifuge (Sigma, Germany), FACSCalibure Flow Cytometer (BD Pharmingen, USA), UV2100 spectrophotometer (UNICO, China), Infinite M200 plate reader (Tecan, Austria), and ELISA reader apparatus (BD Biosciences, USA).

#### Experimental design and setting

Blood sampling was performed on the day of recruitment, on week 26 and whenever there was a suspicion for any adverse event. Sampling was performed at 2 centers; for routine laboratory tests, whole blood samples were submitted directly to the Mousavi Hospital core clinical laboratory. The eligible patients were also referred to the target hospital for sample collection. For cytokine and oxidative stress (except ROS) measurements, whole blood was centrifuged at 3500 rpm for 15 min and separated plasma was allocated in 3 microtubes and stored in an ultra-low temperature freezer tagged with the patients’ unique codes (Biobank). After all measurements the remaining samples were anonymized, and the biobank was discontinued. The process of blood sampling to plasma freezing was less than 60 min for all samples. At the end of each phase (Week 0 and 26) the collected samples were defrosted, and the mentioned biomarkers were measured according to the Kits’ standard protocol.

#### Measurement of intra-cellular lymphocytic ROS

Peripheral blood mononuclear cells (PBMC) isolation procedure was performed as following: (1) whole blood sample was collected using 21-gauge needle in 5 ml lithium heparin vacuum tubes. (2) Fresh heparinized blood was diluted 1:1 ratio with Phosphate-buffered saline (PBS) and mixed well at room temperature. (3) Ficoll–Hypaque density gradient centrifugation was used for PBMCs isolation [8]. (4) 10 ml diluted blood was separated into two conical tubes containing 2 mL ficoll and then centrifuged in a horizontal rotor centrifuge (40 min at 800×*g* at 18 °C). (5) The buffy coat layer was removed and washed with PBS (12 min at 420×*g* at 18 °C). Trypan blue staining was performed to determine viability and counting isolated PBMC. The overall cell viability was more than 90% in all subjects.

Intracellular lymphocytic ROS (including hydrogen peroxide, peroxynitrite, and hydroxyl radical) level was measured using H2DCFDA prob. Given the cell viability assessments the concomitant PI staining did not perform. The Isolated PBMC was divided into 2 tubes of unstained and test with 8 × 10^5^ cells per tube. After titration, 1 µl H2DCFDA in addition to pre-heated PBS were added to the to the isolated PBMC of test tube (final volume = 1 mL) and for the unstained tube, only pre-heated PBS was added (Final volume = 1 mL). Both tubes were incubated at 37 °C in a dark and tightly sealed environment for 40 min. After the incubation period, both tubes were washed twice with PBS (× 1500 rpm for 10 min). Flow cytometry was used to discriminate between lymphocytes and monocytes using the forward and sideward scatter. After lymphocyte zone gating, the mean fluorescent intensity (MFI) of tubes were read on FL1-H Channel [9]. The data obtained by flow-cytometry was analyzed using FlowJo V7.6.5.

#### Performing platelet activation test

Strategies employed to reduce unwanted platelet activation included: (1) blood sampling was performed using 21-gauge needle size, (2) the platelet activation test was performed on whole blood to minimize the time interval between blood collection to platelet activation and repeated physical stress caused by ultra-centrifuging, and (3) the time interval between sample collection to data acquisition was planned to be less than 90 min.

The platelet activation test was performed in biotechnology department in school of pharmacy of Zanjan University of Medical Sciences. The protocol used in this study was obtained by the previously published optimized protocol performed by Huskens et al. [10]. Fresh blood collected into sodium citrate (3.2%) tubes were incubated at 37 °C for 10 min. After that, to minimize the formation of platelet aggregation, the blood was diluted 1:4 in pre-heated N-2-Hydroxyethylpiperazine-N'-2-Ethanesulfonic Acid (HEPES)-buffered saline. From the diluted blood, 20 µl was added to 4 tubes. Based on the label of each tube the following reagents were added: (1) Tube1 (Unstained): 80 µl of preheated HEPES-buffered saline (2) Tube 2 (Negative control): 20 µl of FITC-conjugated anti-P-selectin Ab + 60 µl of preheated HEPES-buffered saline (3) Tube 3 (Isotype control): 20 µl of FITC-conjugated Mouse IgG κ Ab + 60 µl of preheated HEPES-buffered saline (4) Tube 4 (Test): 20 µl of FITC-conjugated anti-P-selectin Ab + 60 µl mixture of preheated HEPES-buffered saline and TRAP (30 µmol/L). The tubes were incubated for exactly 20 min at 37 °C. Flow cytometry used to discriminate platelet from other cells using the forward and sideward scatter both on Log mode using the previous literature [11]. After platelet zone gating, the MFI of tubes were read on FL1-H Channel. The data obtained by flowcytometry was analyzed using FlowJo V7.6.5.

### Statistical consideration

#### Sample size calculation

According to a previous study in which, the SGLT2-i arms (after intervention) had a mean plasma of IL-6 5.8 (pg/ml), and standard deviation (SD) of 8.9. The minimal important difference (MID) was calculated 4.45 using the formula 0.5× SD [12]. The distributional method was applied for calculation of sample size using the following formula (13):

$$n=\frac{{({Z}_{1-\frac{\propto }{2}}+{Z}_{1-\beta })}^{2}\times ({\delta }_{1}^{2}+{\delta }_{0}^{2})}{{(MID)}^{2}}$$

With the study power of 80% and a 2-sided alpha level of 0.05, the sample size was calculated 41 in each arm. Finally, our study intended to randomize 100 patients preparing the required space for the possible dropouts during study. The calculation of our sample size was administered by G-power software (version 3.1.9.2).

#### Statistical analysis

Results from categorical variables are reported as frequencies (percentage) and continuous variables are reported as means (SD). Analysis was done through the intent-to-treat approach which consists of including of all randomized participants in the same group that they were originally allocated. With reference to the central limit theorem, the means (SD) were used for the analysis of the non-normally disturbed continuous data [14]. Ahead of sample size calculation, the primary and secondary outcomes were considered confirmatory and exploratory, respectively. Between-group comparisons of changes from baseline to 26-week of treatment for the primary and secondary outcomes were based on ANCOVA model. Chi-square test was used to analyze categorical variables. Probability value of < 0.05 was considered statistically significant. All statistical tests were carried out in the in Rv.4 environment. The primary data obtained by the FFQ, and flow cytometry was processed by the Nutritionist software version 4 (N4) and FlowJo software V7.6.5, respectively.

## Results

### Baseline and demographic characteristics

**Fig. 1:**
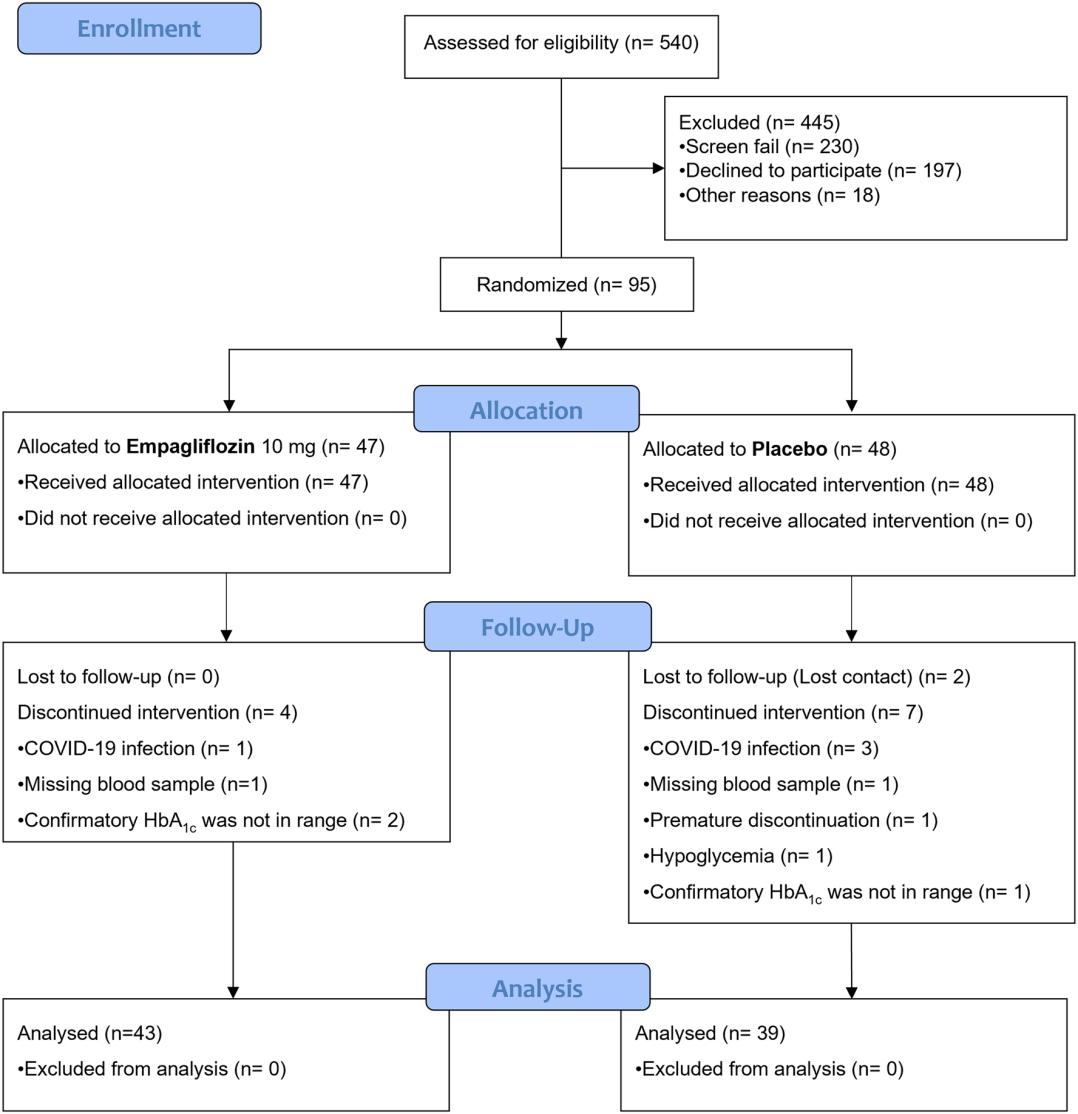
CONSORT flow diagram

After medical record screening, 540 patients met the inclusion criteria, and they were invited for interview during pre-recruitment phase. At the recruitment phase, 95 patients underwent 1:1 randomization and were assigned to receive either empagliflozin 10 mg or placebo. During the treatment period, 13 patients were excluded (4 in empagliflozin and 9 in placebo) (Fig. 1).

Baseline characteristics are summarized in Table 1. Most participants had hypertension (HTN) and hyperlipidemia (HLP) (93.6% and 80.9% in empagliflozin versus 89.6% and 70.8% in placebo group). The majority of participants were being treated with metformin (97.9% versus 95.8%) and had angiotensin enzyme convertase inhibitor (ACEi)/ angiotensin receptor blocker (ARB) as anti-hypertensive medications (87.2% versus 89.6%). Since all of the participants had both T2DM and CAD, almost all of them were already on statins regardless of their past medical history of HLP (97.9% versus 91.7%). The baseline FFQ result indicated no significant difference between two arms regarding the phytochemical index as well as total calorie intake and total carbohydrate intake. Moreover, no significant difference was observed regarding physical activity at baseline. The IPAQ global physical score of patients at baseline showed low to moderate activity. Overall, the 2 groups were well-balanced at entry for demographics and disease characteristics as none of the variables had a significant difference between two arms at baseline (P > 0.05).

**Table 1:** Baseline demographic and laboratory variables randomized in 2 arms

Parameters	Empagliflozin **(n = 47)**	Placebo **(n = 48)**	Mean Diff	P	95% CI
**Demographic**					
Age	62.08 (8.02)	63.60 (7.82)	-1.52	0.352	− 4.75; 1.71
Gender				0.122	
Male	23 (48.9%)	16 (33.3%)			
Female	24 (51.1%)	32 (66.7%)			
Comorbidities (yes)					
HTN	44 (93.6%)	43 (89.6%)		0.714	
HLP	38 (80.9%)	34 (70.8%)		0.339	
Drug history (yes)					
Metformin	46 (97.9%)	46 (95.8%)		0.999	
Sulfonylurea	17 (36.2%)	13 (27.1%)		0.341	
DPP4-i	8 (17.0%)	4 (8.3%)		0.232	
Insulin	4 (8.5%)	3 (6.3%)		0.714	
BB	42 (89.4%)	44 (91.7%)		0.740	
ACEi/ARB	41 (87.2%)	43 (89.6%)		0.720	
CCB	17 (36.2%)	15 (31.3%)		0.612	
Diuretic	20 (42.6%)	20 (41.7%)		0.930	
Statins	46 (97.9%)	44 (91.7%)		0.362	
**Laboratory**					
IL-6 (pg/mL)	4.91 (1.83)	5.19 (2.10)	-0.28	0.492	− 1.10; 0.53
IL-1b (pg/mL)	37.06 (10.36)	34.05 (10.24)	3.00	0.171	− 1.32; 7.32
Hs-CRP (mg/L)	10.07 (8.79)	9.85 (7.90)	0.22	0.901	− 3.29; 3.73
SOD (U/mL)	12.58 (6.32)	11.22 (6.49)	1.36	0.316	− 1.32; 4.05
CAT (mU/mL)	436.19 (148.58)	427.50 (101.81)	8.69	0.747	− 44.67; 62.06
GSHr (muM)	3.09 (0.74)	3.26 (0.95)	-0.17	0.350	− 0.53; 0.19
ROS	1087.25 (687.91)	1010.22 (669.83)	77.03	0.592	− 207.39; 361.46
TAC (mmol/l)	645.38 (80.52)	666.49 (81.70)	-21.11	0.223	− 55.30; 13.08
MDA (nmol/mL)	6.25 (0.83)	6.31 (1.08)	-0.06	0.752	− 0.47; 0.34
PCG (nmol/mg)	0.11 (0.5)	0.12 (0.06)	-0.01	0.568	− 0.03; 0.02
CD62-P Ag Exp	231.26 (37.96)	238.78 (32.93)	-7.53	0.322	− 22.55; 7.50
FBS (mg/dL)	163.14 (40.84)	167.74 (48.64)	-4.60	0.629	− 23.48; 14.28
HbA_1c_ (%)	8.05 (0.97)	7.75 (0.94)	0.30	0.141	− 0.10; 0.70
HOMA-IR	6.52 (3.17)	7.01 (3.48)	-0.49	0.514	− 1.99; 1.00
Calcium (mg/dL)	9.73 (0.51)	9.53 (0.53)	0.21	0.078	− 0.02; 0.44
Sodium (mEq/L)	139.38 (4.34)	138.45 (2.34)	0.93	0.239	− 0.63; 2.48
Potassium (mmol/L)	4.31 (0.37)	4.33 (0.47)	0.87	-0.015	− 0.21; 0.18
WBC count (× 10^9^/L)	7.34 (1.93)	7.08 (1.44)	0.27	0.484	− 0.49; 1.03
Hb (g/dL)	13.87 (1.49)	13.88 (1.57)	-0.00	0.991	− 0.68; 0.67
Hematocrit (%)	41.32 (4.46)	40.76 (4.14)	0.56	0.560	− 1.35; 2.47
Plt count (× 10^9^/L)	240.38 (76.53)	220.21 (55.10)	20.17	0.181	− 9.59; 49.93
BUN (mmol/L)	14.81 (3.95)	16.81 (7.31)	-2.00	0.121	− 4.55; 0.54
Creatinine (mg/dL)	0.98 (0.19)	1.01 (0.23)	-0.04	0.413	− 0.13; 0.05
AST (U/L)	16.95 (6.56)	20.28 (10.21)	-3.33	0.078	− 7.03; 0.38
ALT (U/L)	21.26 (11.52)	23.02 (13.23)	-1.76	0.514	− 7.11; 3.59
PTT (Sec)	32.60 (5.63)	30.86 (5.13)	1.73	0.153	− 0.66; 4.13
**IPAQ**					
Physical Activity (MET min/week)	968.63 (627.53)	871.37 (511.16)	97.26	0.450	− 157.99; 352.51
**FFQ**					
TEI (Kcal/day)	2166.72 (283.78)	2090.49 (337.12)	56.46	0.413	− 80.25; 193.17
PHYs Intake (Kcal/day)	856.42 (203.88)	792.61 (218.19)	59.04	0.232	− 38.47; 156.55
DPI	39.53 (7.61)	28.27 (9.93)	1.39	0.493	− 2.62; 5.39
TPI (g/day)	90.32 (18.47)	84.00 (15.21)	4.80	0.215	− 2.84; 12.44
TSI (g/day)	106.18 (21.34)	109.17 (16.36)	-3.76	0.398	− 12.57; 5.05

Data are presented as mean (SD) or frequency (%)

*HTN* Hypertension, *HLP* Hyperlipidemia, *DPP4-i* Inhibitors of dipeptidyl peptidase-4 inhibitor, *BB* Beta blocker, *ACEi* Angiotensin convertase enzyme inhibitor, *ARB* Angiotensin receptor blocker, *CCB* Calcium channel blocker, *IL-6* Interleukin 6, *IL-1B* Interleukin 1-B, *Hs-CRP* High Sensitivity C-Reactive Protein, *SOD* Superoxide dismutase Enzyme Activity, *CAT* Catalase enzyme activity, *GSHr* Reduced glutathione, *ROS* Reactive oxygen species, *TAC* Total antioxidant Capacity, *MDA* Malondialdehyde, *PCG* Protein carbonyl groups, *CD62-P Ag Exp* CD62-P Ag Expression intensity, *FBS* Fasting blood sugar, *HbA*_*1c*_ Glycated hemoglobin, *HOMA-IR* Homeostatic model assessment for insulin resistance, *WBC* White blood cell, *Hb* Hemoglobin, *Plt* Platelet, *BUN* Bound urea nitrogen, *AST* Aspartate aminotransferase, *ALT* Alanine aminotransferase, *PTT* Partial thromboplastin time, *IPAQ* International physical activity questionnaire, *FFQ* Food frequency questionnaire, *TEI* Total energy intake, *PHYs intake* Phytochemical intake, *DPI* Dietary phytochemical index, *TPI* Total protein intake, *TSI* Total sugar intake

^a^Values are presented as Mean fluorescent intensity (MFI)

^b^Values are presented as the level of PCG/ mg of protein

### Outcomes

#### Primary outcome

**Fig. 2:**
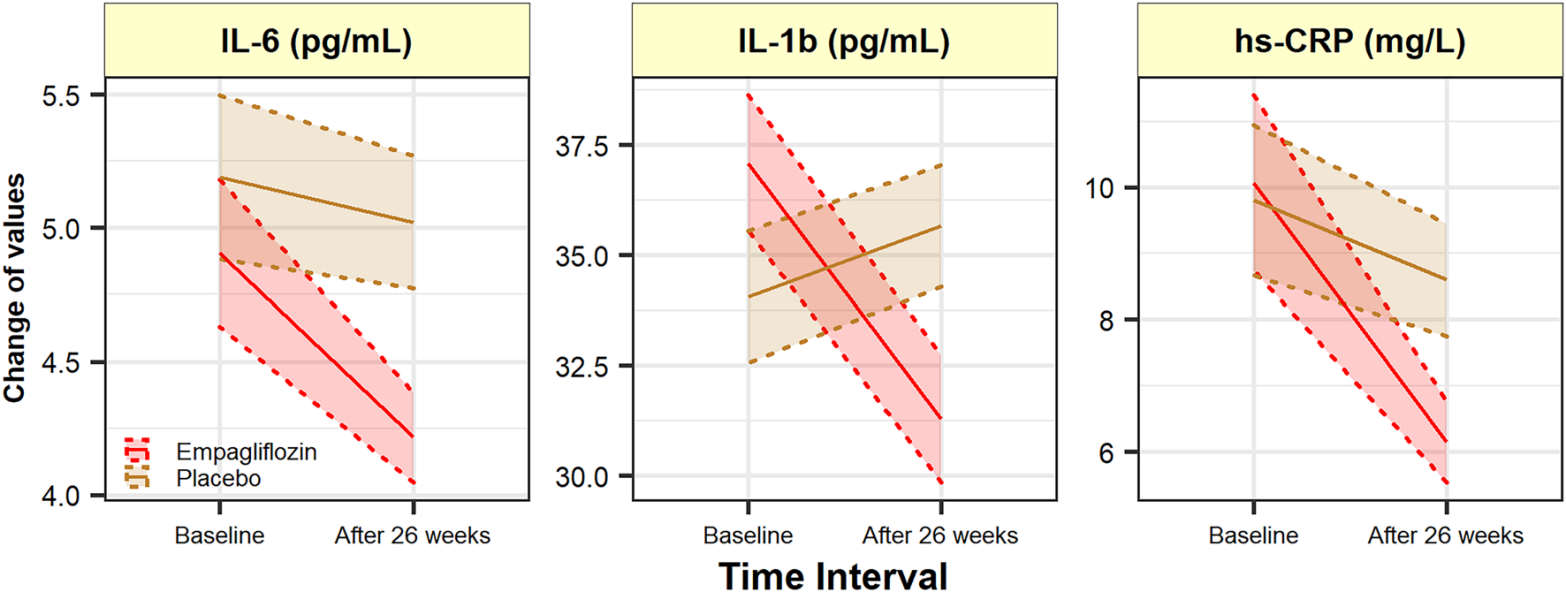
The measured inflammatory biomarkers at baseline and week 26 revealed a significant reduction in all IL-6, IL-1*β*, and Hs-CRP in empagliflozin group compared to placebo (P < 0.05). *IL-6* Interleukin 6, *IL-1 β*: Interleukin 1-beta, *Hs-CRP*: High sensitivity C-reactive protein

Baseline IL-6 was 4.91 (1.83) pg/mL and 5.19 (2.10) pg/mL for the groups assigned to empagliflozin and placebo respectively. The adjusted difference between group at the end of the study was − 1.06 pg/mL (− 1.80; − 0.32), P = 0.006 (Table 2, Fig. 2).

**Table 2:** Changes in laboratory variables among 2 arms

Variables	Empagliflozin n = 43		Placebo n = 39		Mean Diff	P	95% CI
	Baseline	Week 26	Baseline	Week 26			
**Inflammatory**							
IL-6 (pg/mL)	4.91 (1.83)	4.21 (1.13)	5.19 (2.10)	5.22 (1.84)	− 1.06	0.006	− 1.80; − 0.32
IL-1b (pg/mL)	37.06 (10.36)	31.25 (9.58)	34.05 (10.24)	36.48 (10.50)	− 4.58	0.032	− 8.76; − 0.41
Hs-CRP (mg/L)	10.07 (8.79)	6.14 (4.09)	9.85 (7.90)	9.03 (6.35)	− 2.86	0.003	− 4.71; -1.02
**Oxidative stress**							
SOD (U/mL)	12.58 (6.32)	16.91 (8.24)	11.22 (6.49)	12.18 (6.54)	3.70	0.002	1.36; 6.05
CAT (mU/mL)	436.19 (148.58)	462.67 (157.70)	427.50 (101.81)	425.34 (128.23)	32.14	0.329	− 32.99; 97.26
GSH (muM)	3.09 (0.74)	3.70 (0.90)	3.26 (0.95)	3.21 (0.81)	0.57	0.004	0.19; 0.95
ROS^a^	1087.25 (687.91)	769.63 (598.84)	1010.22 (669.83)	1066.95 (679.95)	− 342.51	< 0.001	− 474.23; − 210.79
TAC (mmol/l)	645.38 (80.52)	917.17 (181.02)	666.49 (81.70)	793.51 (136.76)	124.08	0.002	47.98; 200.18
MDA (nmol/mL)	6.25 (0.83)	6.09 (0.53)	6.31 (1.08)	6.24 (0.89)	− 0.13	0.350	− 0.41; 0.15
PCG^b^ (nmol/mg)	0.11 (0.5)	0.09 (0.06)	0.12 (0.06)	0.10 (0.05)	− 0.02	0.222	− 0.04; 0.01
**Platelet function**							
CD62-P Ag Exp^a^	231.26 (37.96)	221.81 (38.51)	238.78 (32.93)	236.56 (34.97)	− 8.81	0.005	− 14.87; − 2.75
**Glycemic status**							
FBS (mg/dL)	163.14 (40.84)	145.23 (41.09)	167.74 (48.64)	169.08 (50.19)	− 18.60	< 0.001	− 25.35; − 11.85
HbA_1c_ (%)	8.05 (0.97)	7.35 (1.04)	7.75 (0.94)	7.82 (0.91)	− 0.65	< 0.001	− 0.83; − 0.48
HOMA-IR	6.52 (3.17)	5.30 (2.89)	7.01 (3.48)	6.94 (3.53)	− 1.18	< 0.001	− 1.47; − 0.89

Data are presented as mean (SD)

Abbreviations used are similar to Table 1

^a^Values are presented as Mean fluorescent intensity (MFI).

^b^ Values are presented as the level of PCG/ mg of protein.

#### Secondary outcomes

**Fig. 3:**
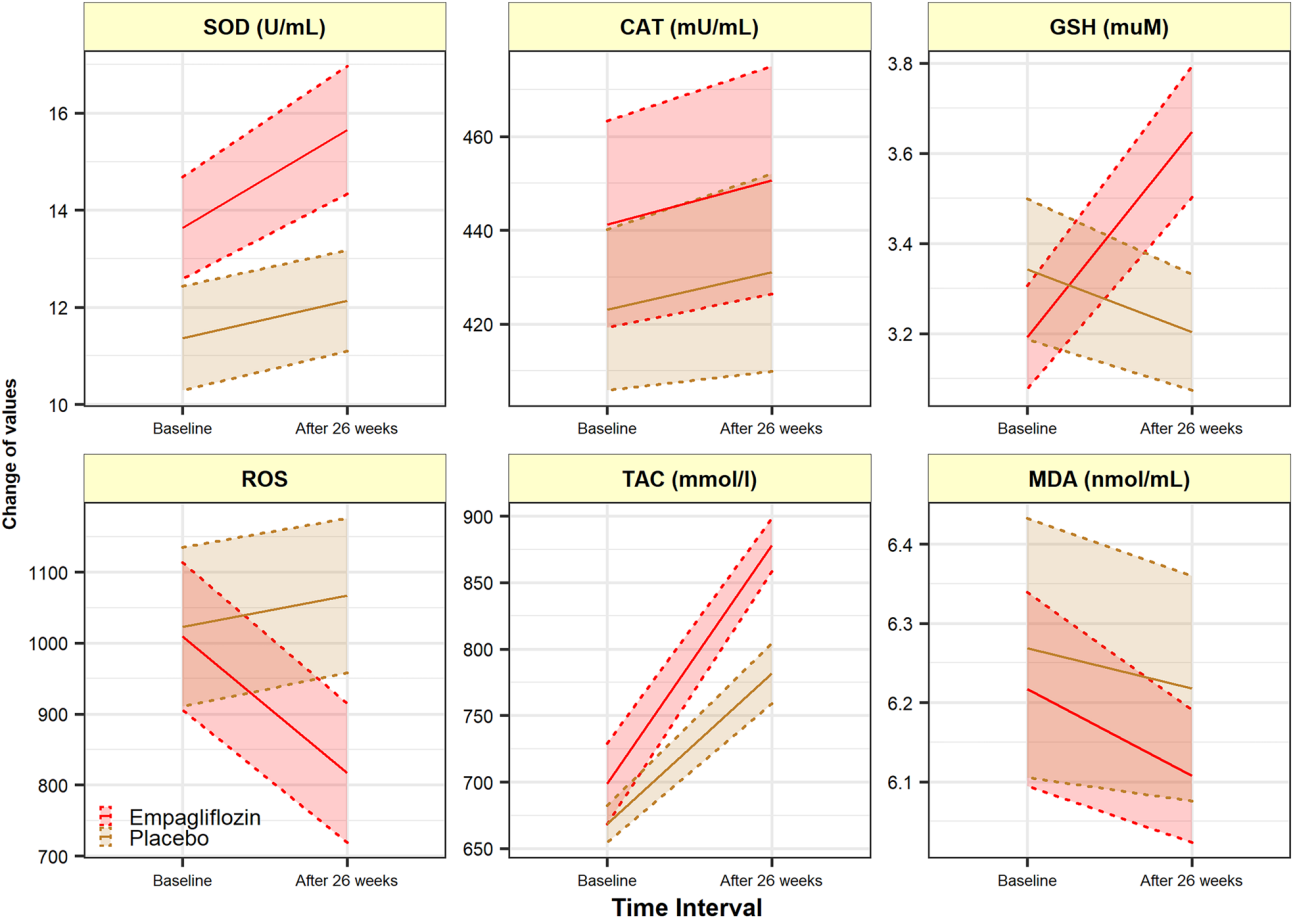
Changes in oxidative stress markers at week 26 from baseline. A significant increase observed in SOD enzyme activity, GSHr, and TAC in empagliflozin group compared to placebo, while a significant reduction observed in lymphocytic ROS (P < 0.05). *SOD*: Superoxidase dismutase, *CAT* Catalase enzyme activity, *GSH* Reduced Glutathione, *ROS* Reactive oxygen species, *TAC* Total antioxidant capacity, *MDA* Malondialdehyde

**Fig. 4:**
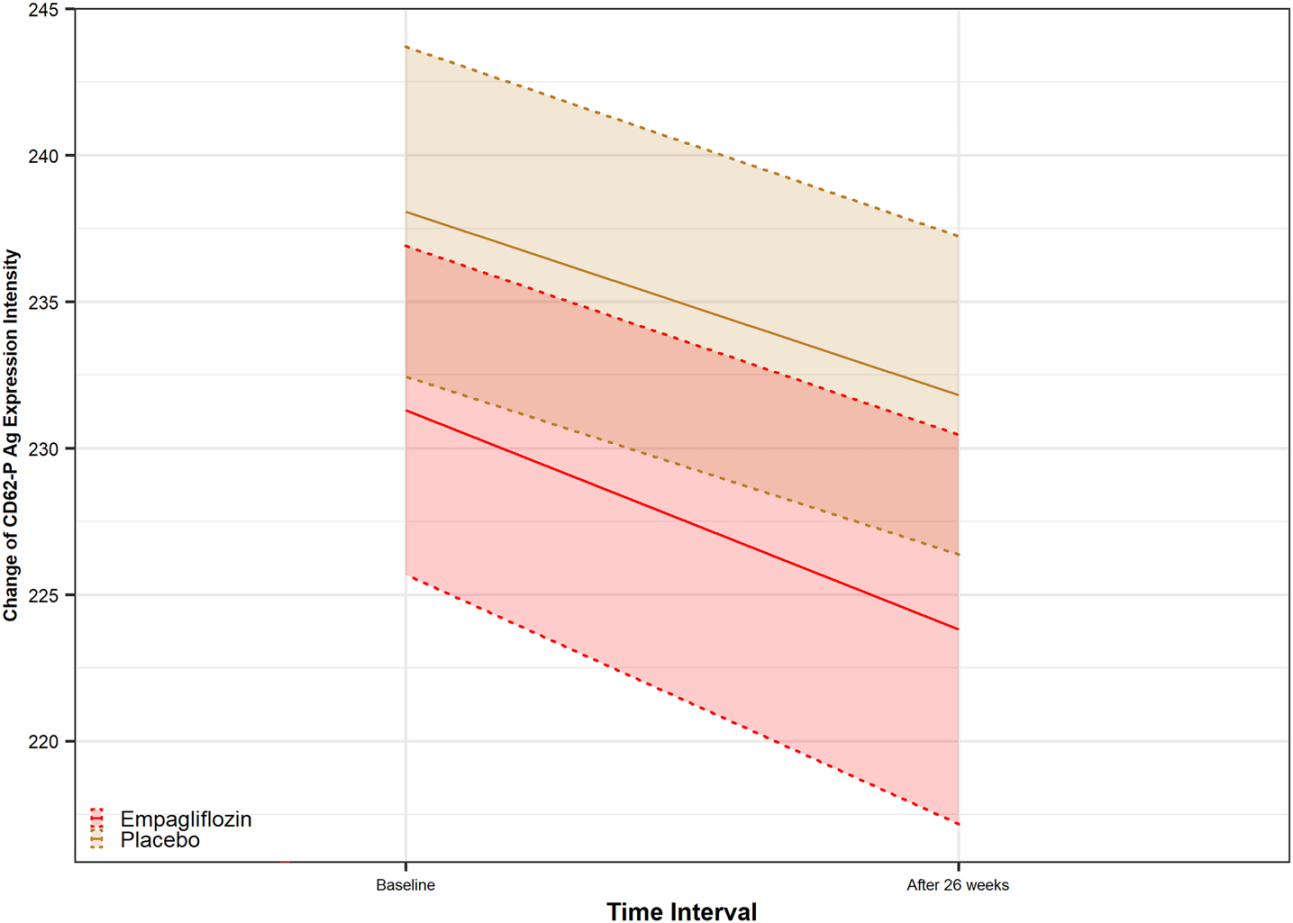
The P-selectin Ag expression on platelet surface changes after 26 weeks of treatment from baseline. A significant reduction observed in mean MFI between 2 study arms (P < 0.05)

Baseline and values at 6-months (adjusted for changes from baseline) for biomarkers of inflammation, oxidative stress, platelet function, and glycemic status are summarized in Table 2. Treatment with Empagliflozin relative to placebo reduced both IL-1*β* and Hs-CRP at 26 weeks [adjusted mean difference: − 4.58 (− 8.76; − 0.41), P = 0.032; and − 2.86 (− 4.71; − 1.02), P = 0.003, respectively] (Fig. 2). The adjusted mean changes for the SOD, GSHr, ROS, and TAC were significantly different between the two study arms [3.70 (1.36; 6.05), P = 0.002; 0.57 (0.19; 0.95), P = 0.004; − 342.51 (− 474.23; − 210.79); P < 0.001); 124.08 (47.98; 200.18); P = 0.002, respectively]. The result of per-patient changes of ROS is presented Additional file 1 in Appendix. Empagliflozin compared to placebo showed neither superiority nor inferiority regarding the adjusted mean changes from baseline for outcomes of CAT, MDA, and PCG [32.14 (− 32.99; 97.26), P = 0.329, observed power = 0.16; 0.350 (− 0.41; 0.15), P = 0.350, observed power = 0.15; − 0.02 (− 0.04; 0.01), P = 0.222, observed power = 0.23, respectively] (Fig. 3). After 26 weeks of treatment with empagliflozin, P-selectin Ag expression on the activated platelets’ surface decreased significantly [231.26 (37.96) to 221.81 (38.51) versus 238.78 (32.93) to 236.56 (34.97), with an adjusted mean difference: -8.81 (-14.87; -2.75), P = 0.005] (Fig. 4). The result of per-patient changes of CD62P Ag expression is presented in Additional file 2 in the Appendix.

**Fig. 5:**
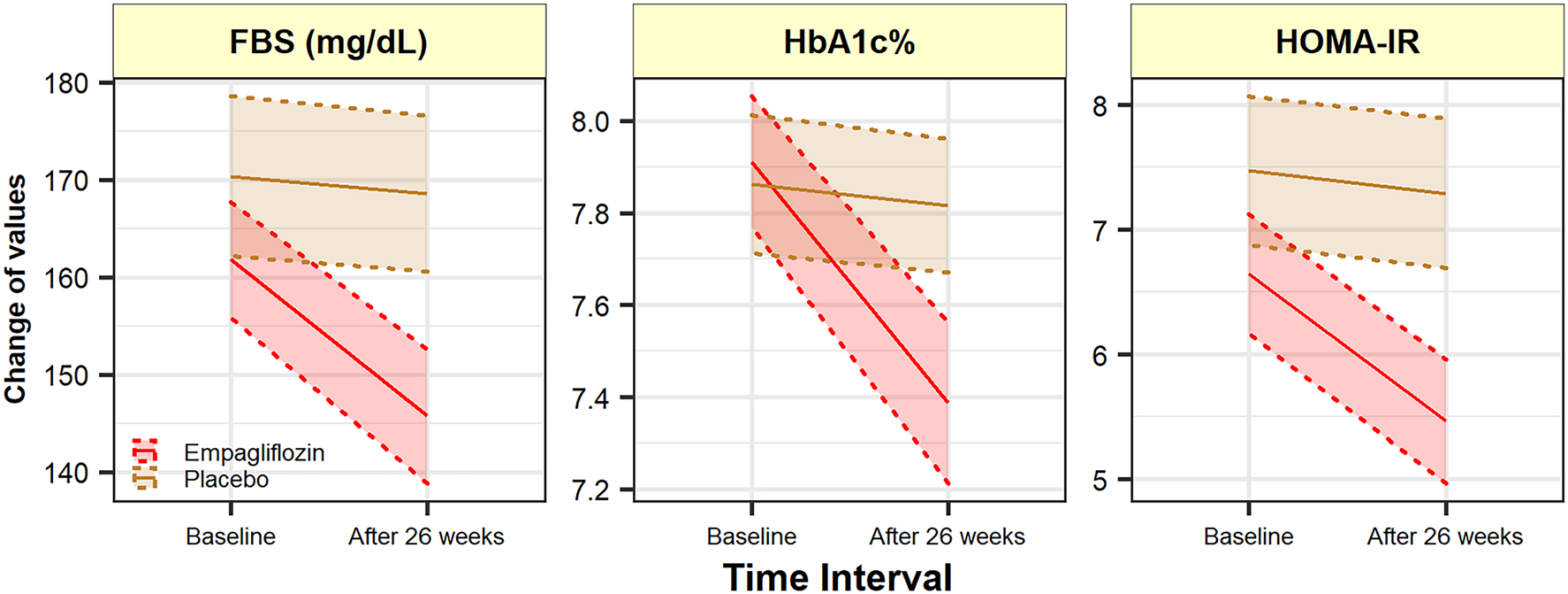
Changes of glycemic status and insulin resistance after 26 weeks of treatment from baseline. Compared to placebo, empagliflozin has reduced all the markers of glycemic status after the treatment period (P > 001). *FBS* Fasting blood sugar, *HbA*_*1c*_ Glycated Hemoglobin, *HOMA-IR* Homeostatic model assessment for insulin resistance

With regards to the glycemic status, empagliflozin compared to placebo had significantly reduced both FBS and HbA_1c_ after treatment [adjusted mean difference: − 18.60 (− 25.35; − 11.85); P < 0.001; and − 0.65 (− 0.83; − 0.48), P < 0.001, respectively]. Moreover, HOMA-IR as the index of insulin resistance was also significantly reduced in empagliflozin compared to placebo [adjusted mean difference: − 1.18 (− 1.47; − 0.89), P < 0.001) (patients who used insulin as anti-diabetic therapy were excluded from this analysis) (Fig. 5).

### Adverse events

No serious drug related adverse event was observed during and after 2 weeks of treatment. The incidences of adverse events during follow-up were 4.26% (2/43) in empagliflozin and 2.08% (1/39) in placebo. The 2 patients in the placebo group were hospitalized for COVID-19 infection. No deaths had occurred during the study period in patients who had not been lost to follow up. Totally, the treatment was well tolerated in both empagliflozin and placebo groups.

## Discussion

EMPA-CARD trial demonstrated that compared with placebo, the addition of 10 mg empagliflozin to standard antihyperglycemic treatment in participants with concomitant T2DM and CAD was associated with a significant reduction in proinflammatory biomarkers. Moreover, empagliflozin substantially ameliorated oxidative stress.

Based on our review of the literature, there has not been a RCT to address the status of oxidative stress in patient with T2DM. As discussed previously, IL-6 is an important source for promoting systemic inflammation in diabetic patients which results in development and progression of cardiovascular complication, particularly arterial atherosclerosis [7]. This present study revealed that empagliflozin can significantly reduce the level of IL-6 after 26 weeks of treatment. Several molecular pathways have been proposed in animal models and in vitro studies for this effect. mRNA expression of pro-inflammatory cytokines such as IL-6 and IL-1*β* are tightly regulated by the signaling pathways of NF-*κ*B, AMP-activated protein-kinase (AMPK) and Janus tyrosine kinase/signal transducer and activator of transcription (JAK/STAT) in pro- and anti-inflammatory responses [15]. On the other hand, T2DM has been shown to promote foam cell formation in hyperactive macrophages in arterial walls and accelerate the progression of atherosclerotic plaque [16]. In an in vitro model, empagliflozin suppressed the secretion of IL-6, IL-1*β* and tumor necrosis factor-alpha (TNF-*α*) by blocking the NF-*κ*B, c-Jun N-terminal kinases (JNK), and STAT1/3 phosphorylation in RAW 264.7 M1 macrophages [17]. Other animal models suggested that empagliflozin may have provided its cardiovascular and renal protection by means of reducing pro-inflammatory cytokines (IL-6, TNF-*α* and Monocyte Chemoattractant Protein-1 [MCP-1]) through down-regulating NF-*κ*B and activating AMPK [18, 19]. The antioxidant systems activate several pathways to scavenge free radicals and transform them into lower hazardous substances in order to reduce cellular damage and regulate the level of systemic inflammation. T2DM and CAD are characterized with chronic inflammatory processes that markedly enhance oxidative stress and as a result overwhelm the antioxidant defense system capacity. Therefore, oxidative products such as ROS and nitric oxide (NO^−^), MDA and PCG are increased. The SOD/GSHr/CAT mechanisms are believed to be the first-line antioxidant defense and play fundamental role in enzymatic defense against free radicals [20]. Dysregulation of this mechanism in immune cells, particularly endothelial neutrophils and macrophages, is reported to be associated with micro- and macro-vascular complications of DM [21]. In the present report levels of SOD and GSHr were significantly increased with empagliflozin. CAT enzyme activity, however, had a non-significant increase in empagliflozin group. Enhancement of SOD/GSHr/CAT activity in the empagliflozin group is associated with reduced levels of ROS. In addition, a novel pathway has been introduced that empagliflozin attenuates ROS and cellular sodium through inhibition of Na^+^/H^+^ exchanger in endothelial cells [22]. Subsequent to the increase in TAC, the level of Hs-CRP as one of the clinical products of inflammation was also reduced. Although the production of the measured oxidative biomarkers were in line with the changes in ROS, based on our results; MDA and PCG had the smallest reductions [23].

Empagliflozin modestly increases circulatory free fatty acids and blood ketones (probably by inducing lipolysis) both of which are substrates for formation of MDA and PCG [24, 25]. Besides the formation of MDA through the degradation in polyunsaturated lipid triggered by ROS, MDA could also be a product of metabolized prostaglandin-H2 in the prostacyclin synthetase pathway [26]. Concerning the reno-protective effect of SGLT2 inhibitors which is comprised of an increase in renal prostaglandin synthesis and urinary secretion, we hypothesize that the production of MDA may have been increased slightly in light of the rise in prostaglandins, free fatty acids, and blood ketone bodies; furthermore the potential contributing role of MDA attachment to amino acids to higher PCG content is of note, and hence the impact of decreased ROS in lowering MDA and PCG was relatively neutralized [27, 28]. However, no human model RCT has been conducted to evaluate the latter markers. The available data are discrepant and are mostly from studies on animal models [29–31]. Lambadiari et al. reported similar results on the changes in MDA after 4 and 12 months of therapy with empagliflozin [32].

The molecular effects of empagliflozin on platelet activation and function have not yet been ascertained. In this trial, empagliflozin significantly reduced the expression of P-selectin on the activated platelet surface. It has been shown that in a hyperglycemic state, chronic inflammation can lead to platelet hyperactivity. Given the higher rate of anti-platelet therapy resistance in diabetic patients, this hyperactivity is associated with increased adverse outcomes in patients with CAD [33]. So far, no RCT study has been conducted to investigate the effect of empagliflozin on platelet function. Despite some findings that failed to highlight enhancement of platelet aggregation or prothrombic effects of empagliflozin, there are promising evidence from the results of an open label single arm EFFECT pilot study that revealed the attenuative effect of empagliflozin on platelet reactivity in patients with stable CAD and T2DM on dual antiplatelet therapy regimen [34, 35]. Empagliflozin is thought to alter platelets function through decreasing systematic inflammation. Moreover, it has been shown that the antiplatelet activity of gliflozins are strongly enhanced by prostacyclin and nitric oxide that emphasizes the effects of this drug class on cardiovascular protection that are subsequent to platelet inhibition (36).

## Limitations

Our study has some limitations. First, CD62P antibodies were the only ones used to assess platelet activation Since CD62P merely expresses platelet activation, its decline does not distinguish whether the decrease was in the number of activated platelets or further activation of formerly activated platelets. The differentiation could have been yielded by means of measuring CD42 antigens which we were not able to employ. Second, our small to moderate number of patients might have contributed to the non-significant increase in CAT levels, and inclusion of larger number of patients in future studies may lead to more significant changes. Third, we measured each variable in serum samples once at baseline and then at the endpoint; thus, the fluctuation of data throughout the study remained unclear, repeated measure designs avail us with the assessment of the changes over time. Finally, our intervention period was limited to 6 months, and longer duration of treatment could impact the obtained values.

## Conclusion

Previous evaluations of treatment with Empagliflozin have revealed that itcan confer major clinical cardiovascular benefits to patients with T2DM. The present study was designed to investigate the impact of Empagliflozin on the molecular changes that might contribute to the improvements in cardiovascular outcomes in patients with T2DM. Our results highlight the anti-oxidative and anti-inflammatory properties of Empagliflozin added to the standard anti-diabetic therapy in patients with both T2DM and CAD. Hence, use of Empagliflozin can be considered as a reasonable approach for prevention or treatment of diabetic cardiomyopathy.

## Supplementary Information

**Additional file 1:** The Flow Cytometric assay results of per-patient levels of ROS at baseline (**B**) and week 26 (**A**).

**Additional file 2:** The Flow Cytometric assay results of per-patient levels of platelet CD62P Ag expression at baseline (**B**) and week 26 (**A**).

## Data Availability

The data/information supporting this study is available from the corresponding author upon reasonable request.

## Abbreviations

T2DM: Type 2 diabetes mellitus
ROS: Reactive oxygen species
IL-6: Interleukin 6
SGLT2-i: Sodium glucose co-transporter-2 inhibitor
CAD: Coronary artery disease
RCT: Randomized controlled trial
CAG: Coronary angiography
NYHA class: New York Heart Association class
LVEF: Left ventricular ejection fraction
BMI: Body mass index
TIA: Transient ischemic attack
ACS: Acute coronary syndrome
CVA: Cerebrovascular accident
CABG: Coronary artery bypass graft
PCI: Percutaneous coronary intervention
DKA: Diabetic ketoacidosis
FFQ: Food frequency questionnaire
IPAQ: International physical activity questionnaire
2D-TTE: 2-Dimentional transthoracic echocardiography
Hs-CRP: High-sensitivity C-reactive protein
IL-1*β*: Interleukin 1-beta
SOD: Superoxide dismutase
GSHr: Reduced glutathione
CAT: Catalase
TAC: Total antioxidant capacity
MDA: Malondialdehyde
PCG: Protein carbonyl groups
FBS: Fasting blood sugar
HOMA-IR: Homeostatic model assessment for insulin resistance
TRAP: Thrombin receptor activator peptide
PBMC: Peripheral blood mononuclear cells
PBS: Phosphate-buffered saline
H2DCFDA: 2'-7'Dichlorofluorescin diacetate
MFI: Mean fluorescent intensity
HEPES: N-2-Hydroxyethylpiperazine-N'-2-Ethanesulfonic Acid
MID: Minimal important difference
HTN: Hypertension
HLP: Hyperlipidemia
ACEi: Angiotensin enzyme convertase inhibitor
ARB: Angiotensin receptor blocker
NF-*κ*B: Nuclear factor kappa B
AMPK: AMP-activated protein-kinase
JAK/STAT: Janus tyrosine kinase/signal transducer and activator of transcription
TNF-*α*: Tumor necrosis factor-alpha
JNK: C-Jun N-terminal kinase
MCP-1: Monocyte chemoattractant protein-1
NO^−^: Nitric oxide
