# Supplementary figures and images for: The effect of EMPAgliflozin on markers of inflammation in patients with concomitant type 2 diabetes mellitus and Coronary ARtery Disease: the EMPA-CARD randomized controlled trial (part 2 of 2)
Source: Diabetol Metab Syndr. 2022 Nov 17;14:170. doi: 10.1186/s13098-022-00951-5 (PMC9669535; doi:10.1186/s13098-022-00951-5)

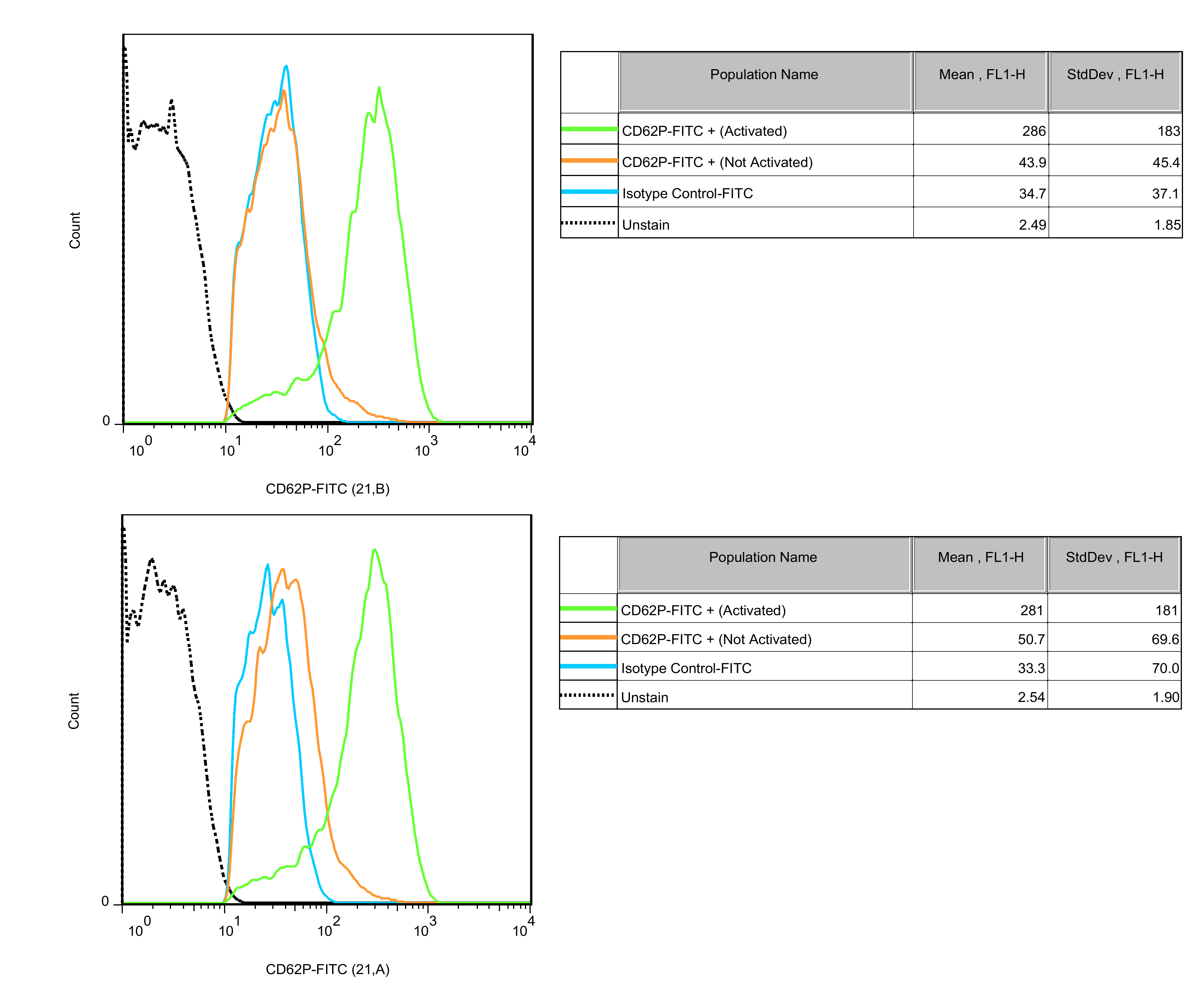

Supplement: Supplementary file 2 — Additional file 2: The Flow Cytometric assay results of per-patient levels of platelet CD62P Ag expression at baseline (B) and week 26 (A). [file 13098_2022_951_MOESM2_ESM.zip › 21.png]

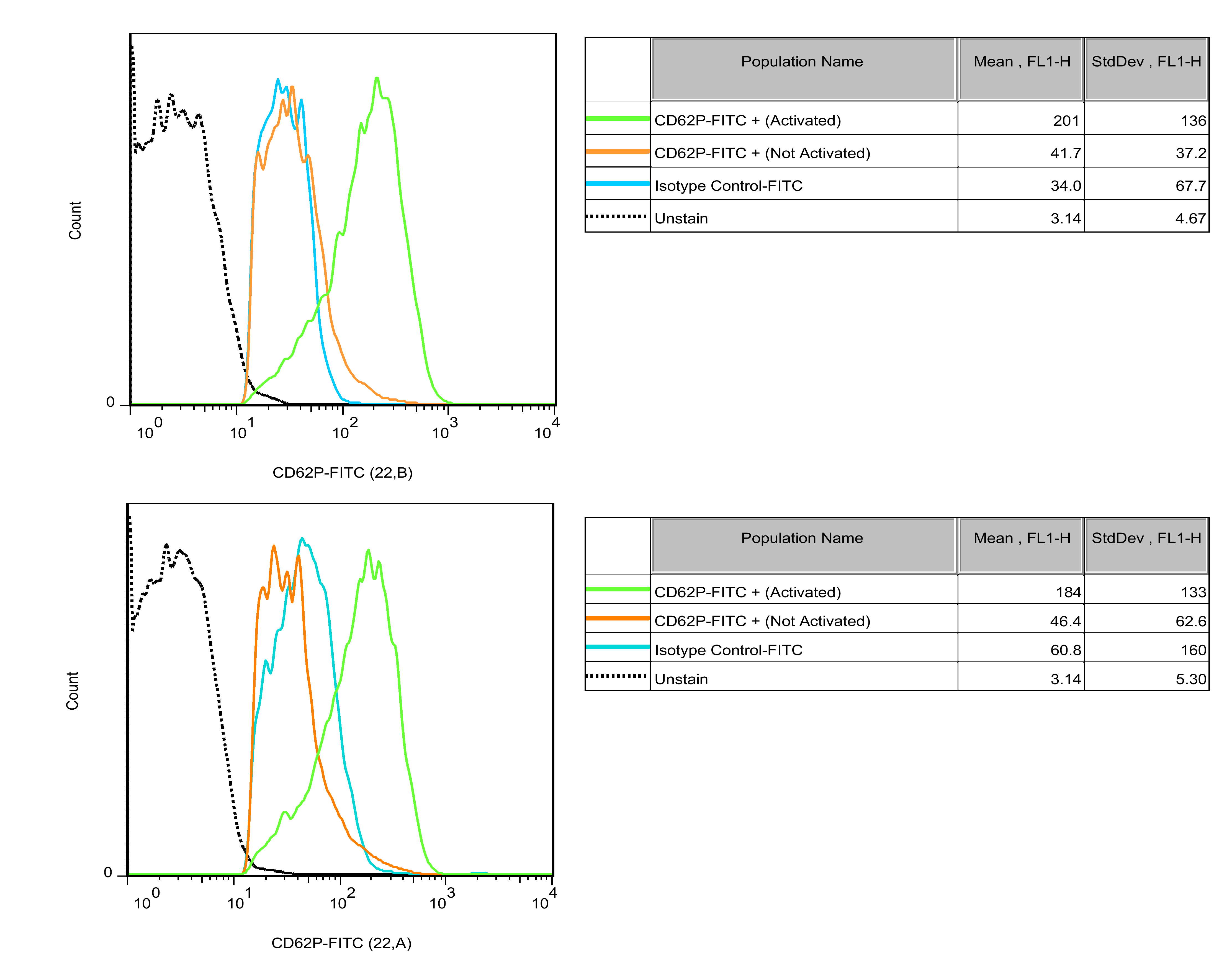

Supplement: Supplementary file 2 — Additional file 2: The Flow Cytometric assay results of per-patient levels of platelet CD62P Ag expression at baseline (B) and week 26 (A). [file 13098_2022_951_MOESM2_ESM.zip › 22.png]

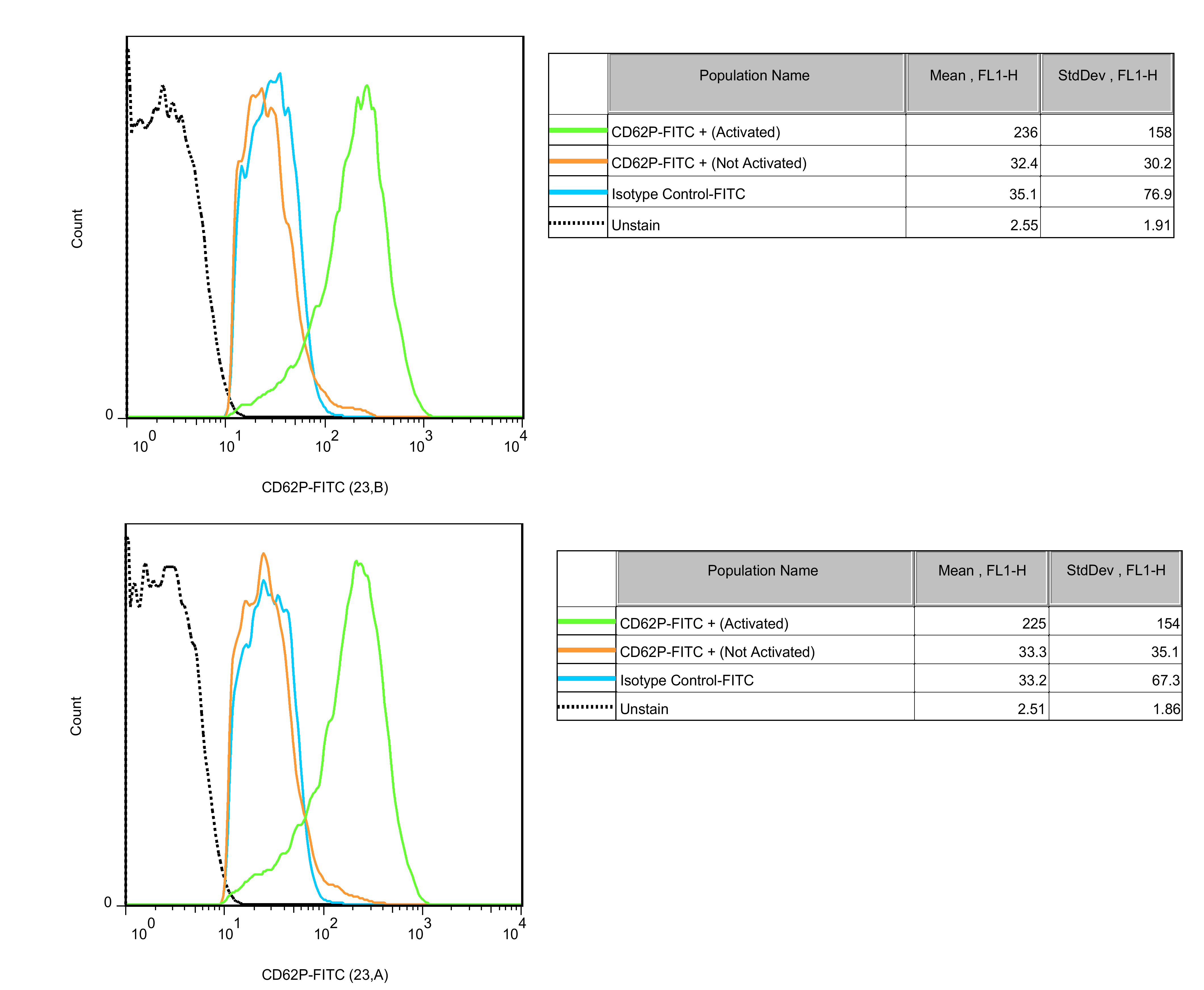

Supplement: Supplementary file 2 — Additional file 2: The Flow Cytometric assay results of per-patient levels of platelet CD62P Ag expression at baseline (B) and week 26 (A). [file 13098_2022_951_MOESM2_ESM.zip › 23.png]

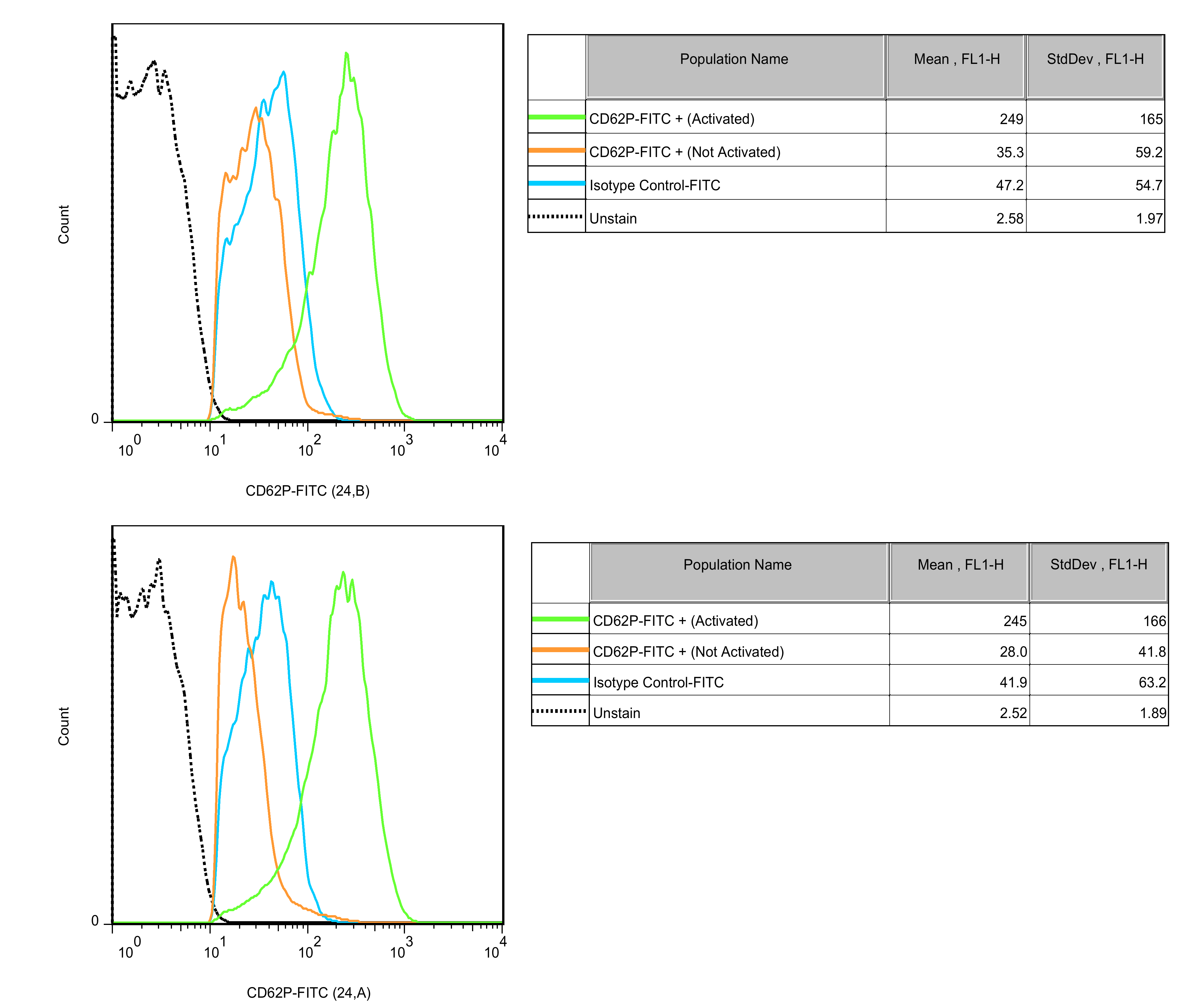

Supplement: Supplementary file 2 — Additional file 2: The Flow Cytometric assay results of per-patient levels of platelet CD62P Ag expression at baseline (B) and week 26 (A). [file 13098_2022_951_MOESM2_ESM.zip › 24.png]

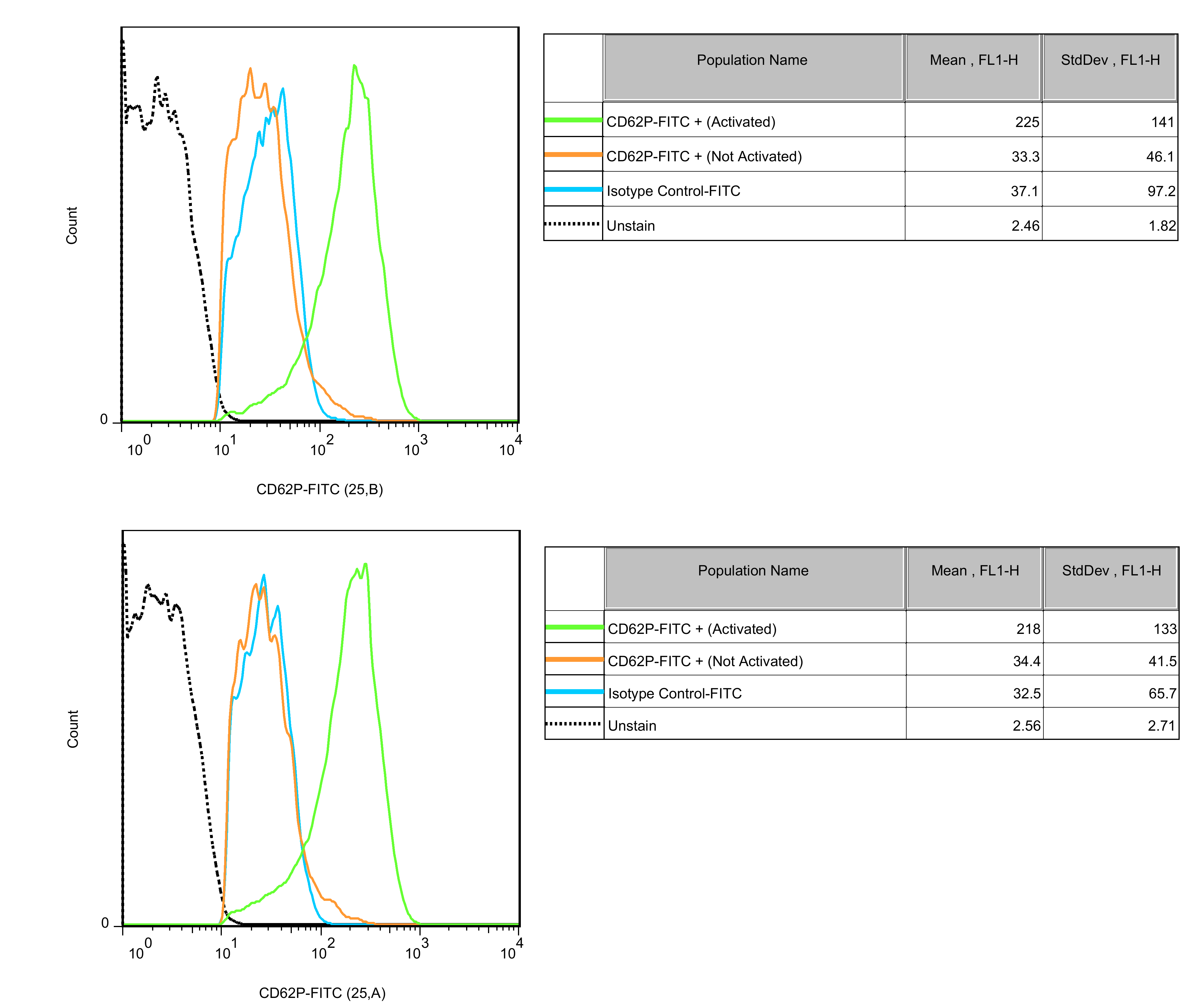

Supplement: Supplementary file 2 — Additional file 2: The Flow Cytometric assay results of per-patient levels of platelet CD62P Ag expression at baseline (B) and week 26 (A). [file 13098_2022_951_MOESM2_ESM.zip › 25.png]

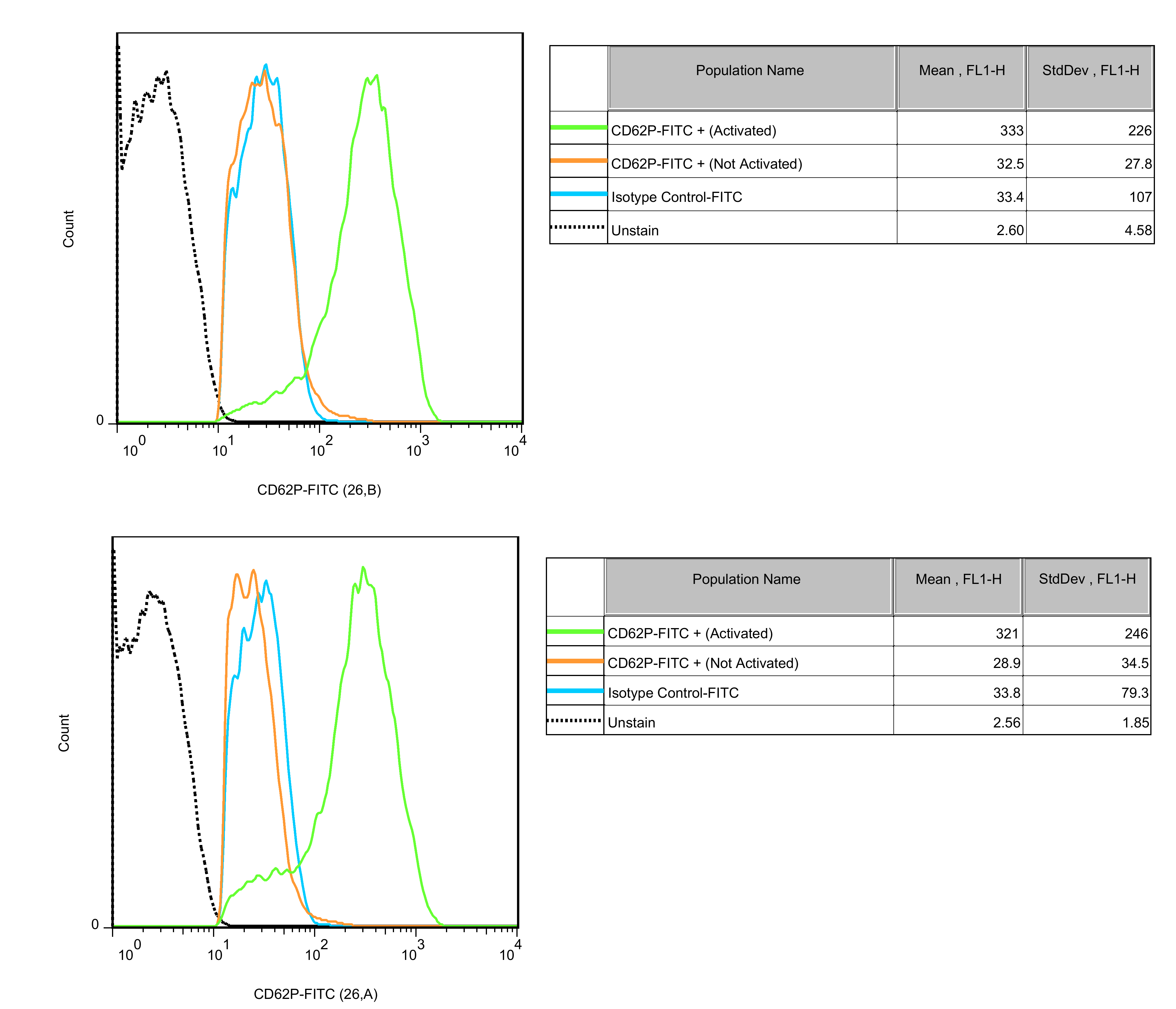

Supplement: Supplementary file 2 — Additional file 2: The Flow Cytometric assay results of per-patient levels of platelet CD62P Ag expression at baseline (B) and week 26 (A). [file 13098_2022_951_MOESM2_ESM.zip › 26.png]

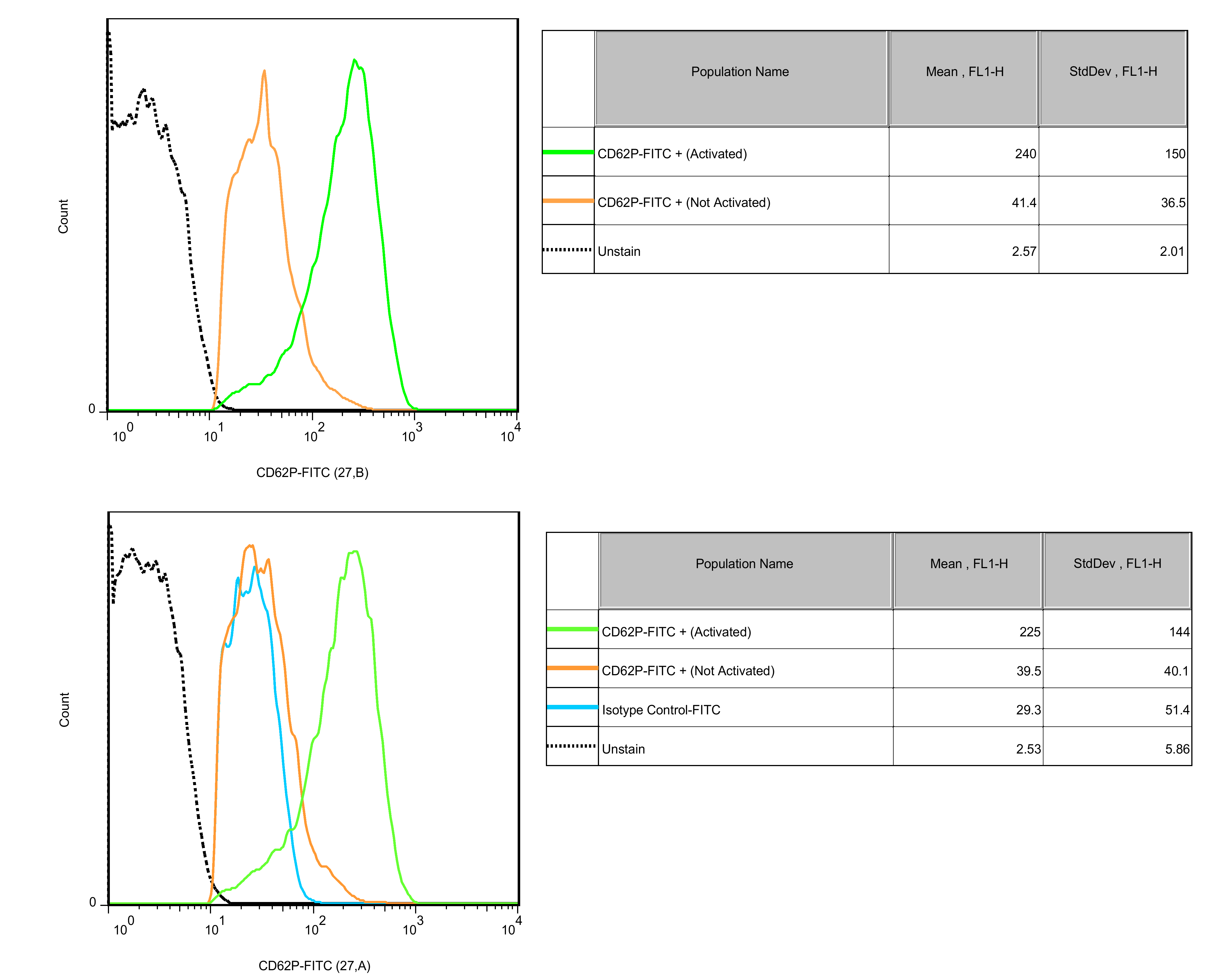

Supplement: Supplementary file 2 — Additional file 2: The Flow Cytometric assay results of per-patient levels of platelet CD62P Ag expression at baseline (B) and week 26 (A). [file 13098_2022_951_MOESM2_ESM.zip › 27.png]

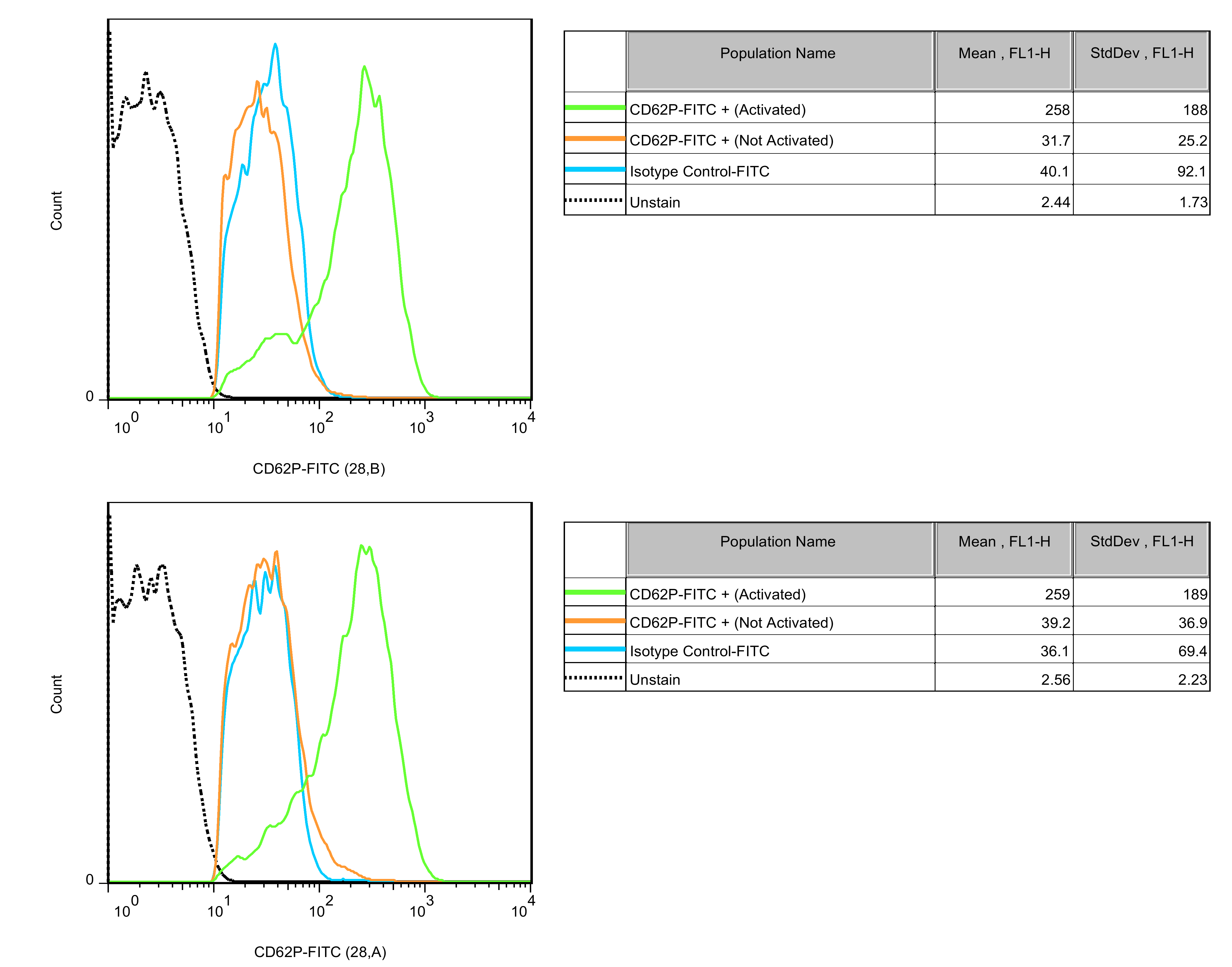

Supplement: Supplementary file 2 — Additional file 2: The Flow Cytometric assay results of per-patient levels of platelet CD62P Ag expression at baseline (B) and week 26 (A). [file 13098_2022_951_MOESM2_ESM.zip › 28.png]

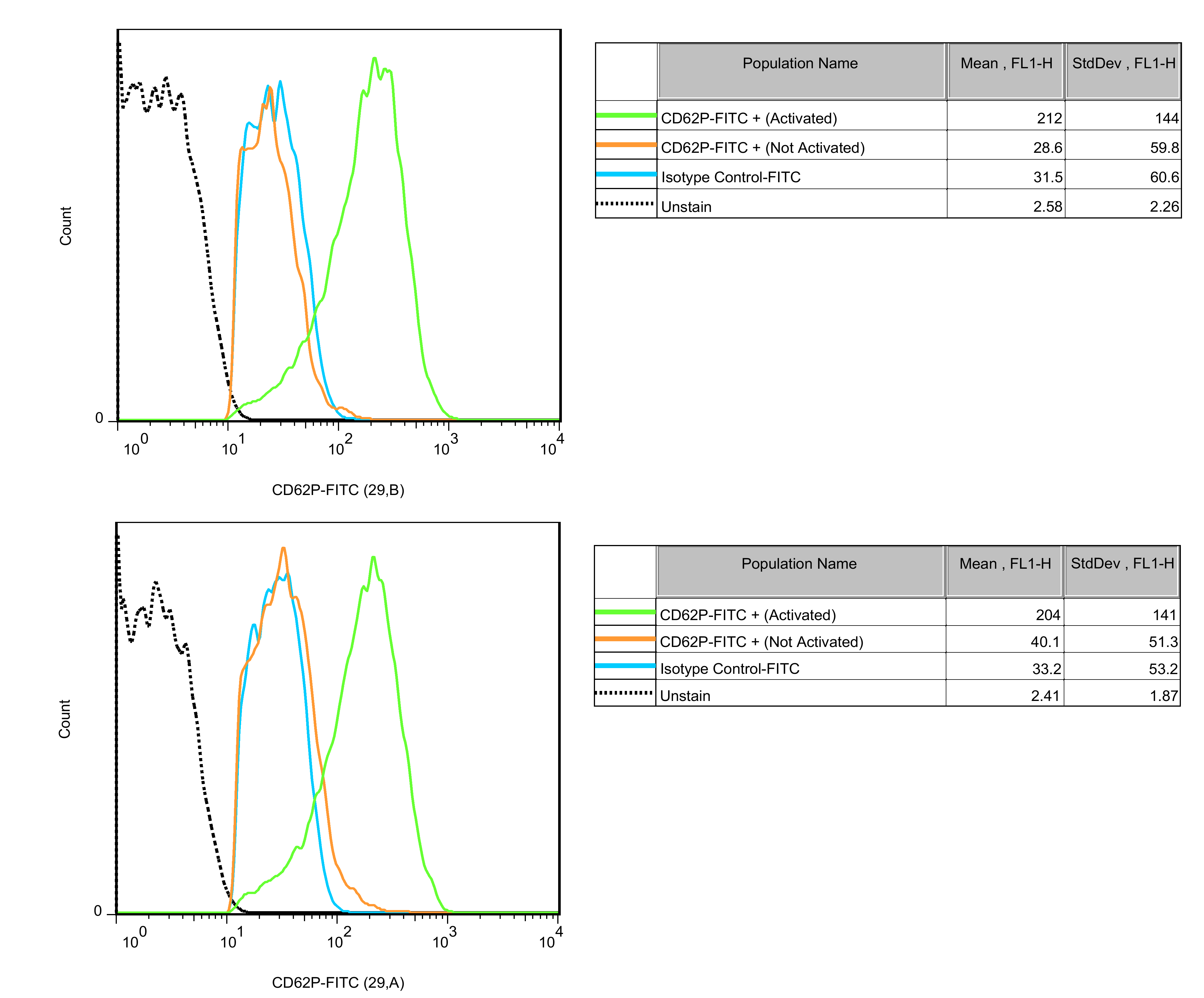

Supplement: Supplementary file 2 — Additional file 2: The Flow Cytometric assay results of per-patient levels of platelet CD62P Ag expression at baseline (B) and week 26 (A). [file 13098_2022_951_MOESM2_ESM.zip › 29.png]

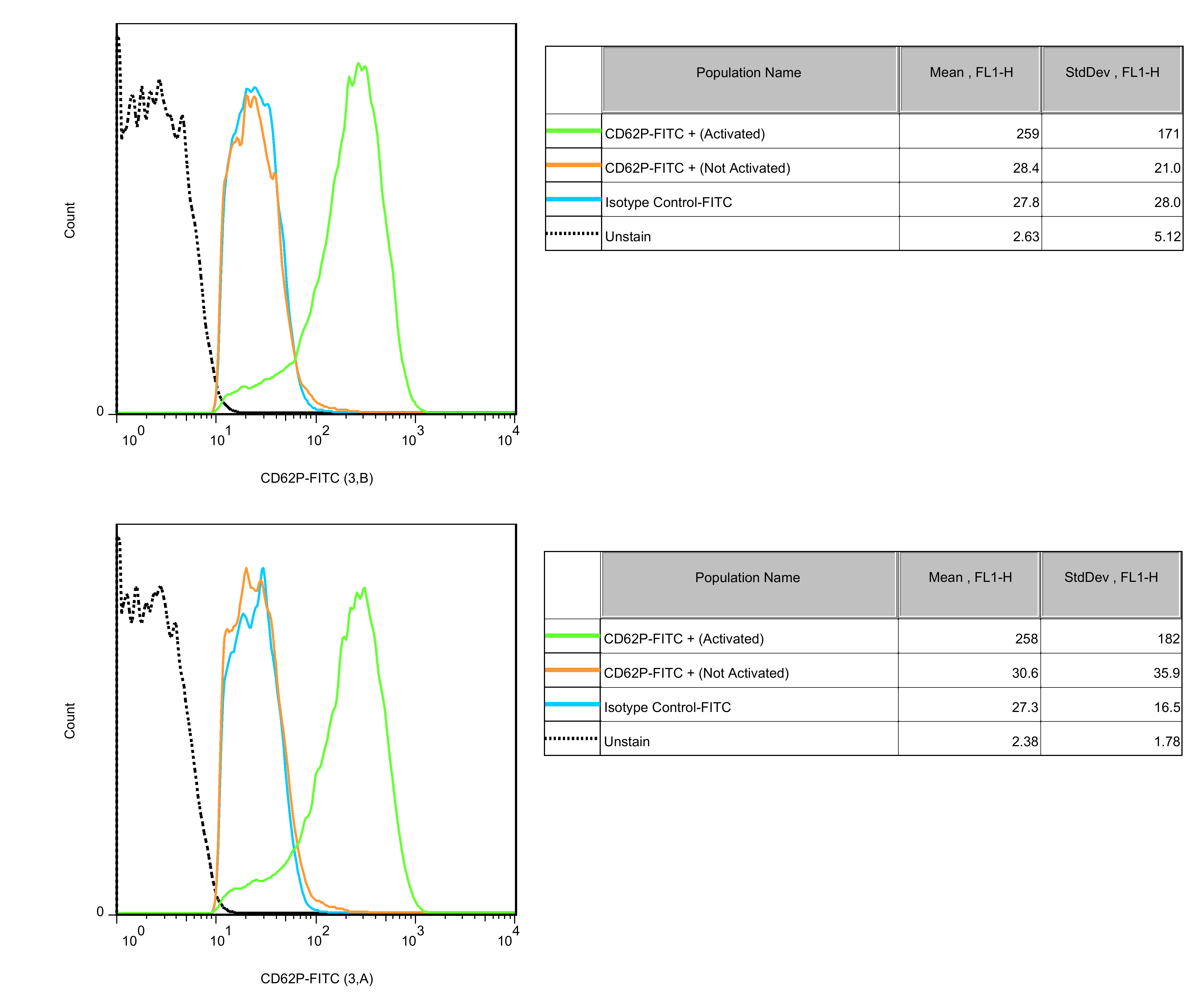

Supplement: Supplementary file 2 — Additional file 2: The Flow Cytometric assay results of per-patient levels of platelet CD62P Ag expression at baseline (B) and week 26 (A). [file 13098_2022_951_MOESM2_ESM.zip › 3.png]

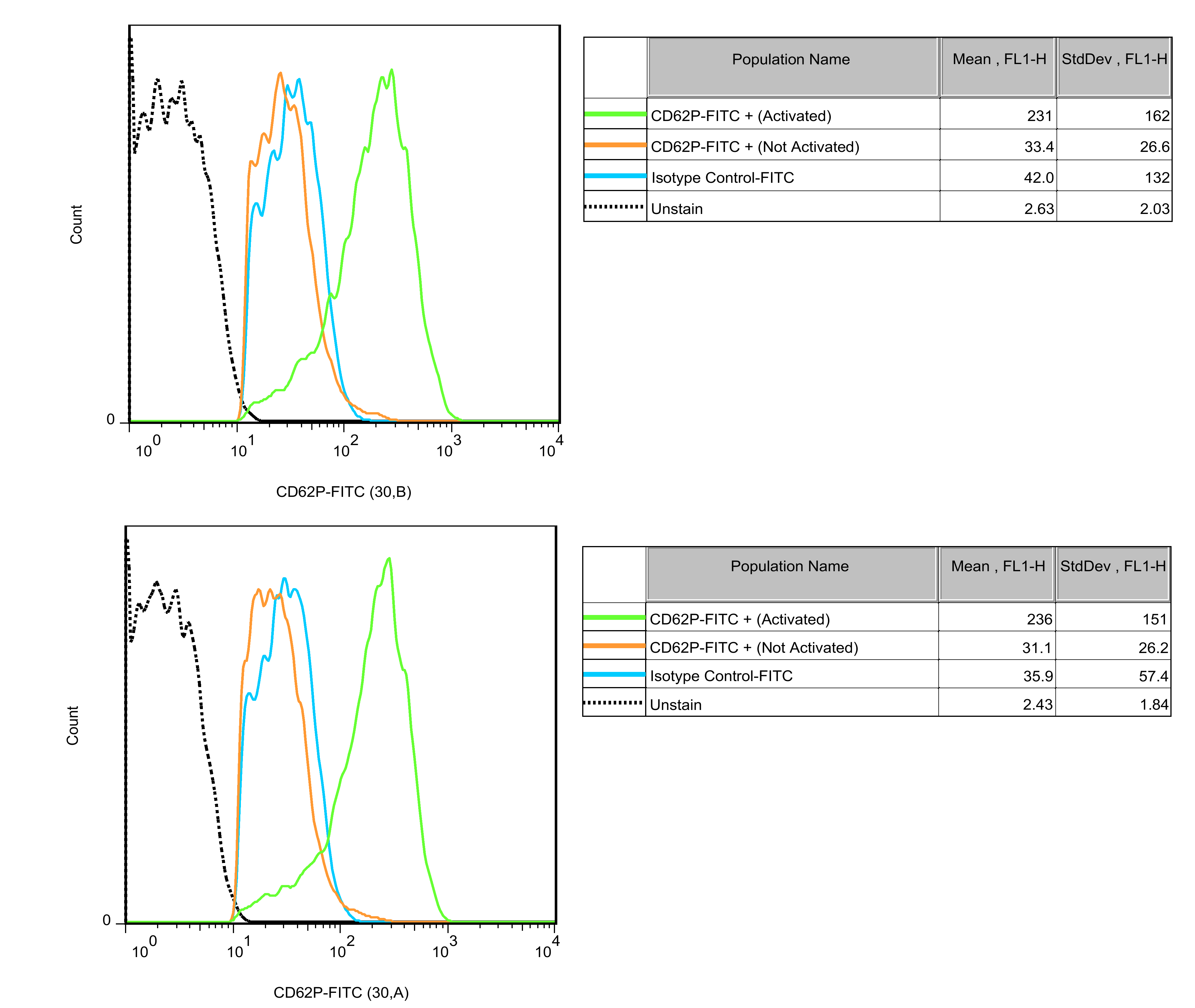

Supplement: Supplementary file 2 — Additional file 2: The Flow Cytometric assay results of per-patient levels of platelet CD62P Ag expression at baseline (B) and week 26 (A). [file 13098_2022_951_MOESM2_ESM.zip › 30.png]

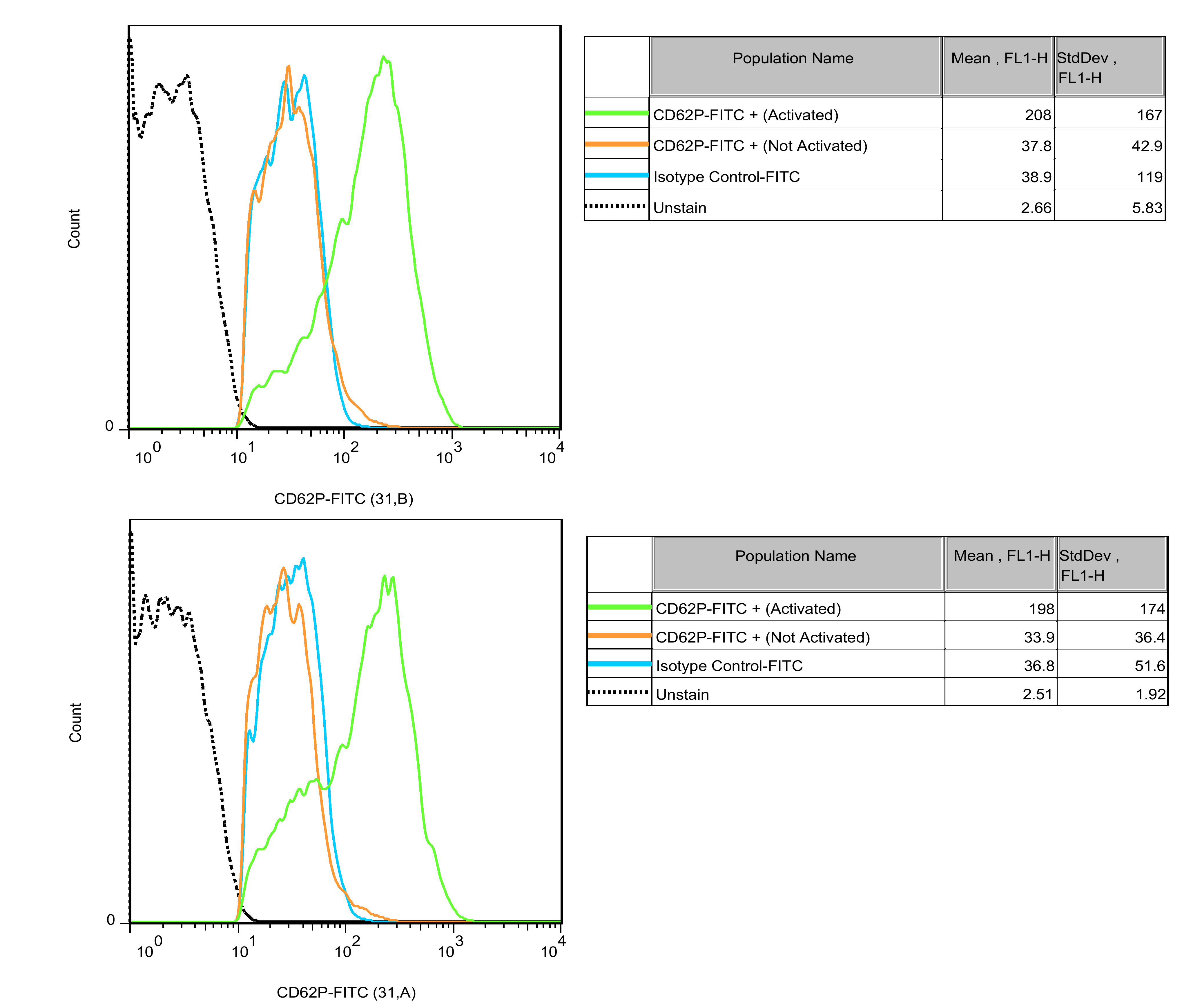

Supplement: Supplementary file 2 — Additional file 2: The Flow Cytometric assay results of per-patient levels of platelet CD62P Ag expression at baseline (B) and week 26 (A). [file 13098_2022_951_MOESM2_ESM.zip › 31.png]

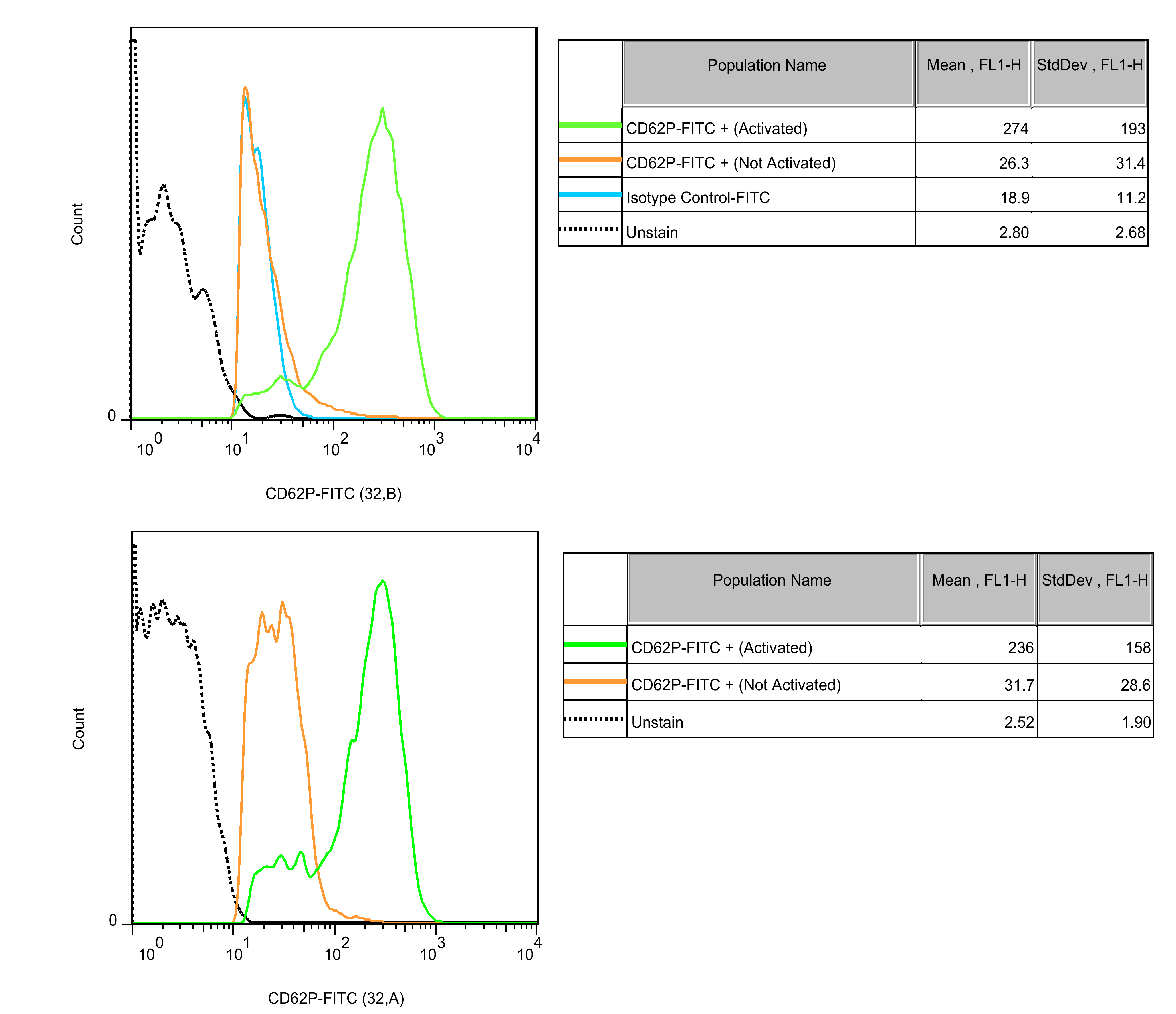

Supplement: Supplementary file 2 — Additional file 2: The Flow Cytometric assay results of per-patient levels of platelet CD62P Ag expression at baseline (B) and week 26 (A). [file 13098_2022_951_MOESM2_ESM.zip › 32.png]

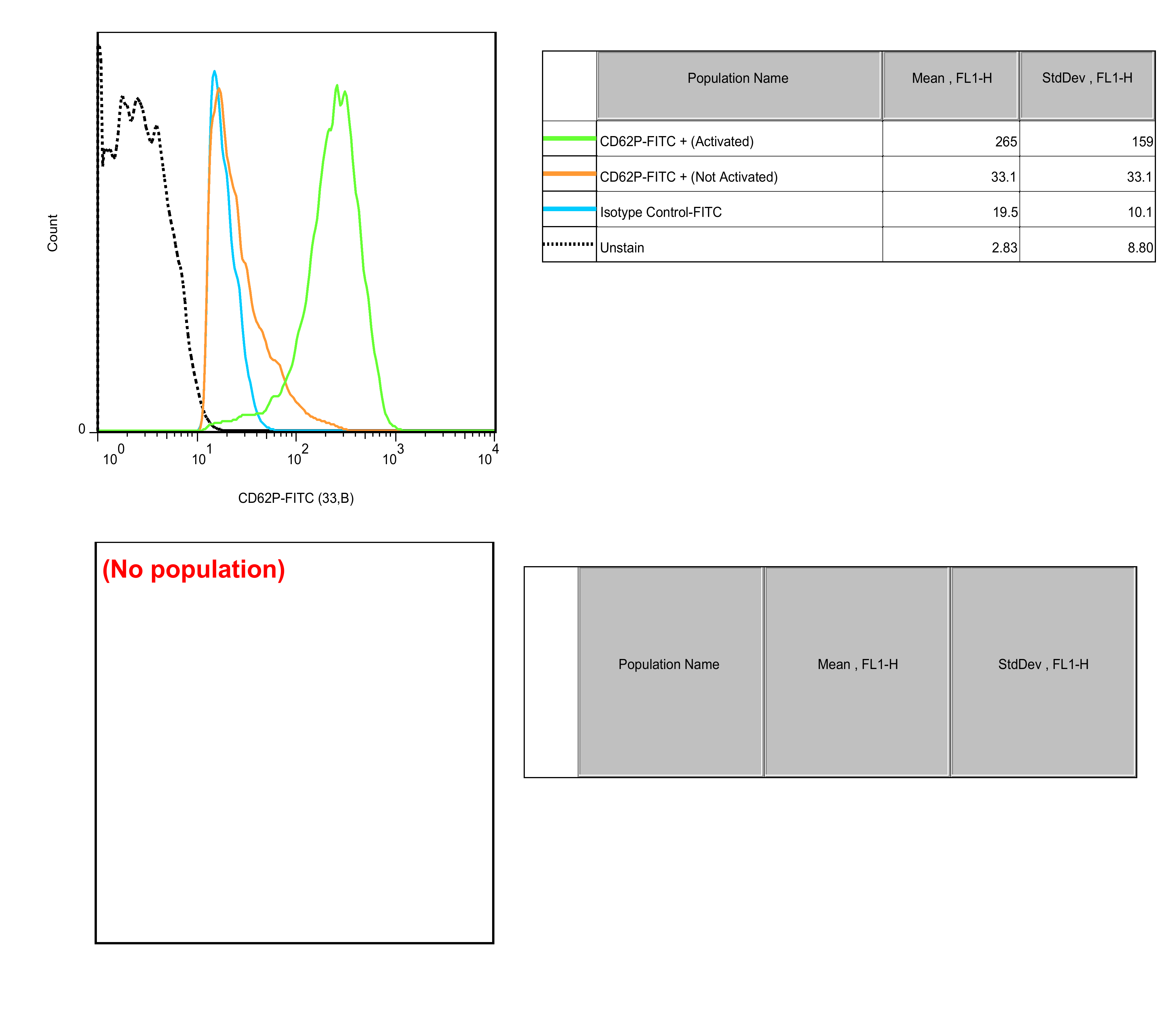

Supplement: Supplementary file 2 — Additional file 2: The Flow Cytometric assay results of per-patient levels of platelet CD62P Ag expression at baseline (B) and week 26 (A). [file 13098_2022_951_MOESM2_ESM.zip › 33.png]

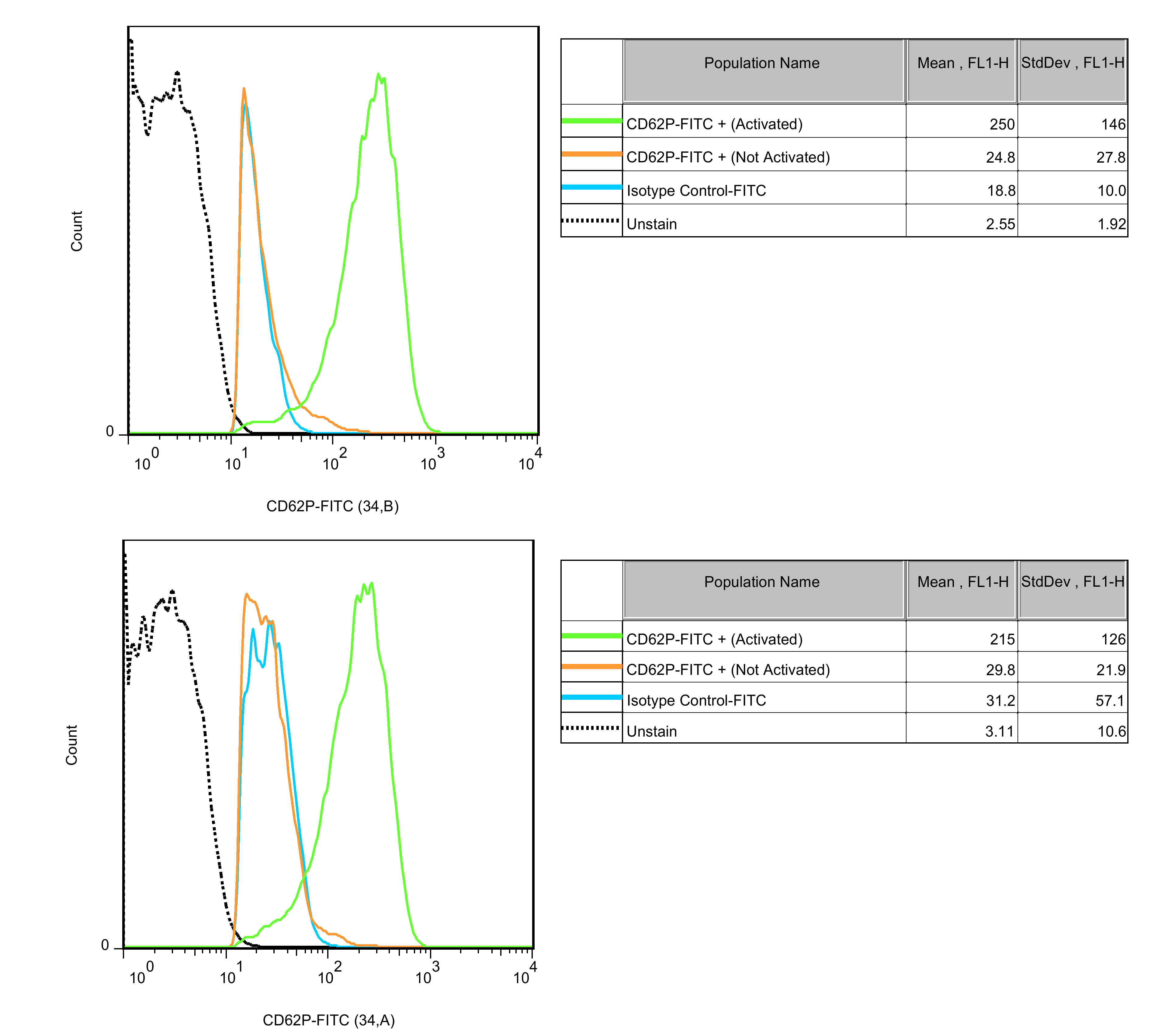

Supplement: Supplementary file 2 — Additional file 2: The Flow Cytometric assay results of per-patient levels of platelet CD62P Ag expression at baseline (B) and week 26 (A). [file 13098_2022_951_MOESM2_ESM.zip › 34.png]

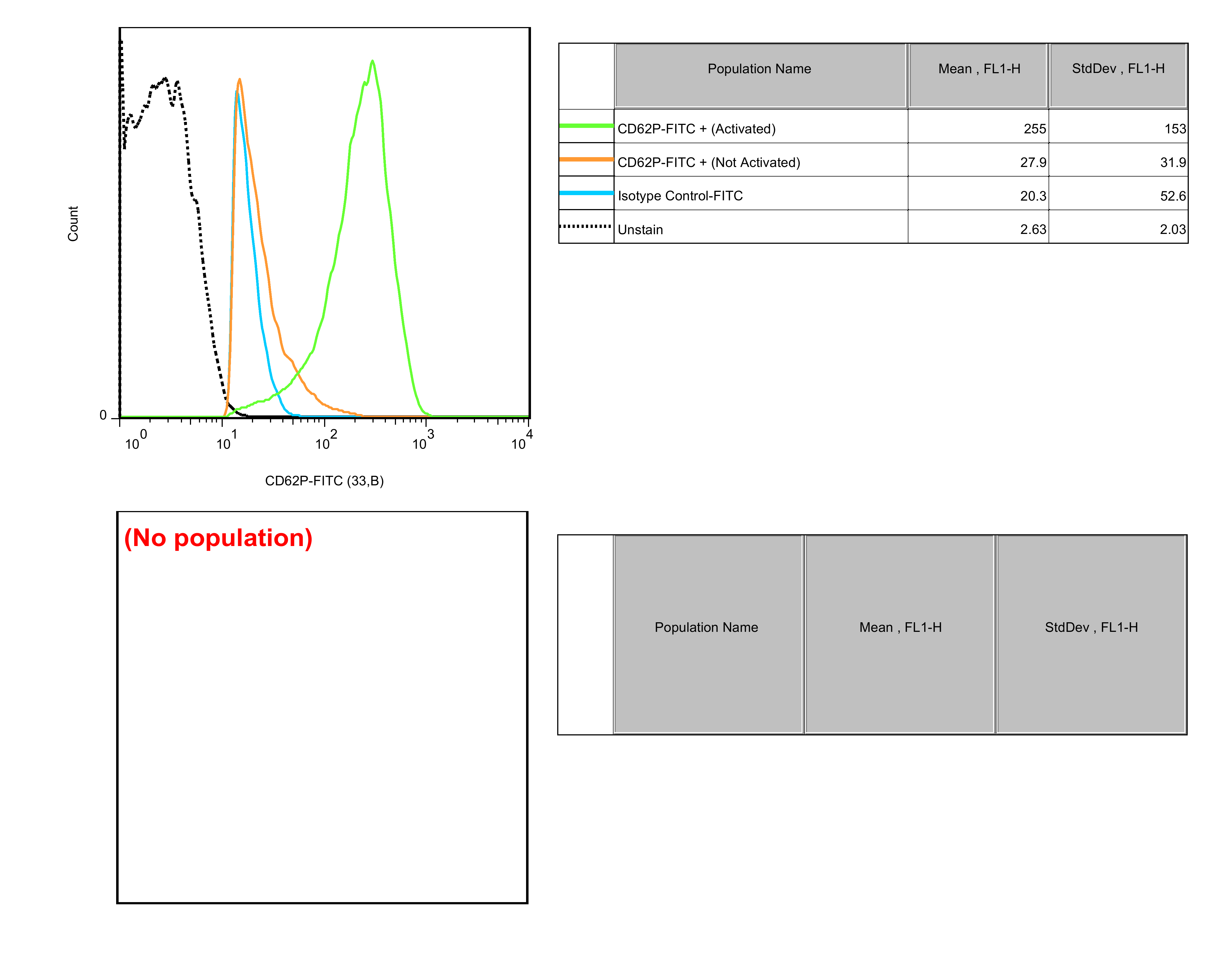

Supplement: Supplementary file 2 — Additional file 2: The Flow Cytometric assay results of per-patient levels of platelet CD62P Ag expression at baseline (B) and week 26 (A). [file 13098_2022_951_MOESM2_ESM.zip › 35.png]

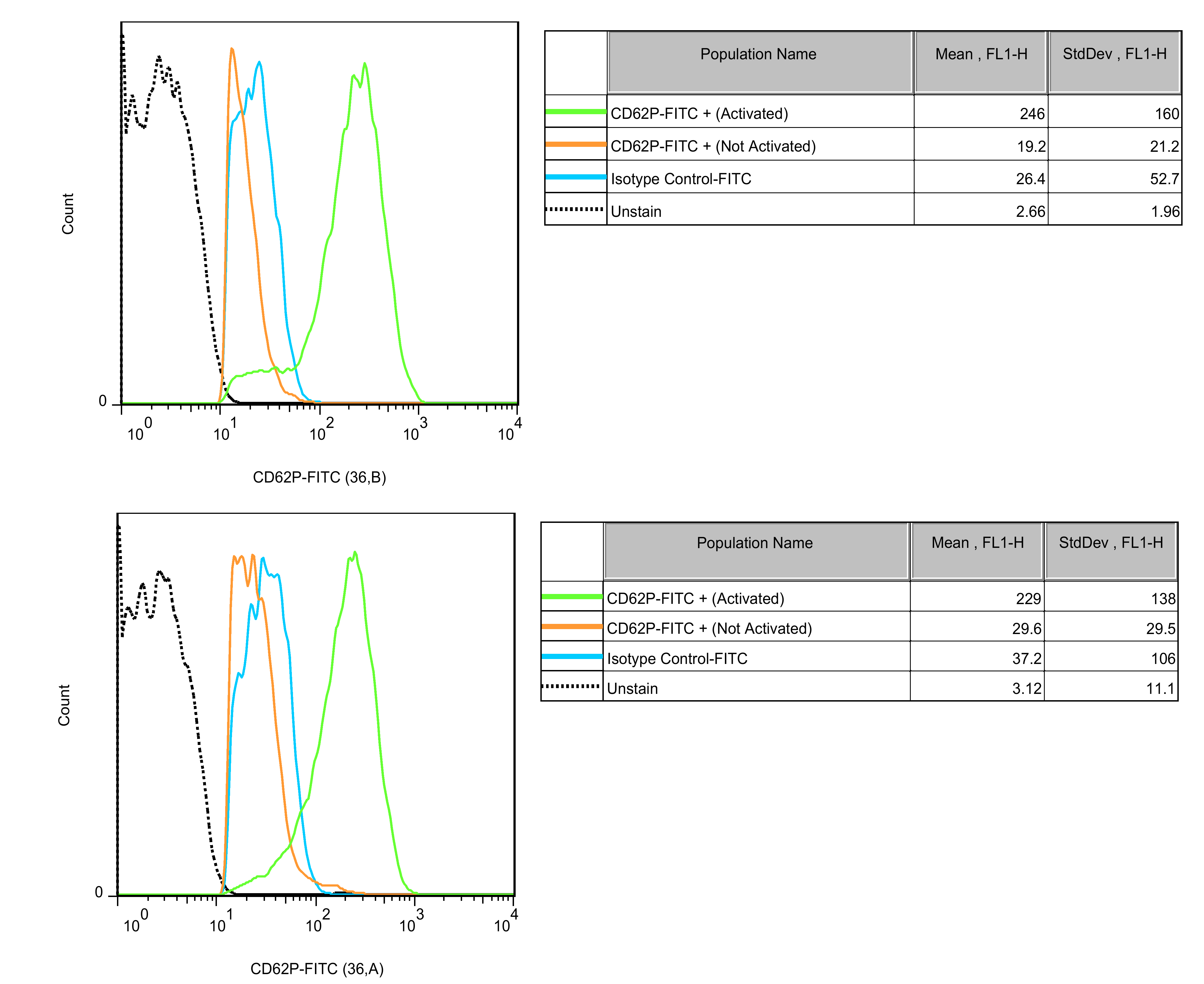

Supplement: Supplementary file 2 — Additional file 2: The Flow Cytometric assay results of per-patient levels of platelet CD62P Ag expression at baseline (B) and week 26 (A). [file 13098_2022_951_MOESM2_ESM.zip › 36.png]

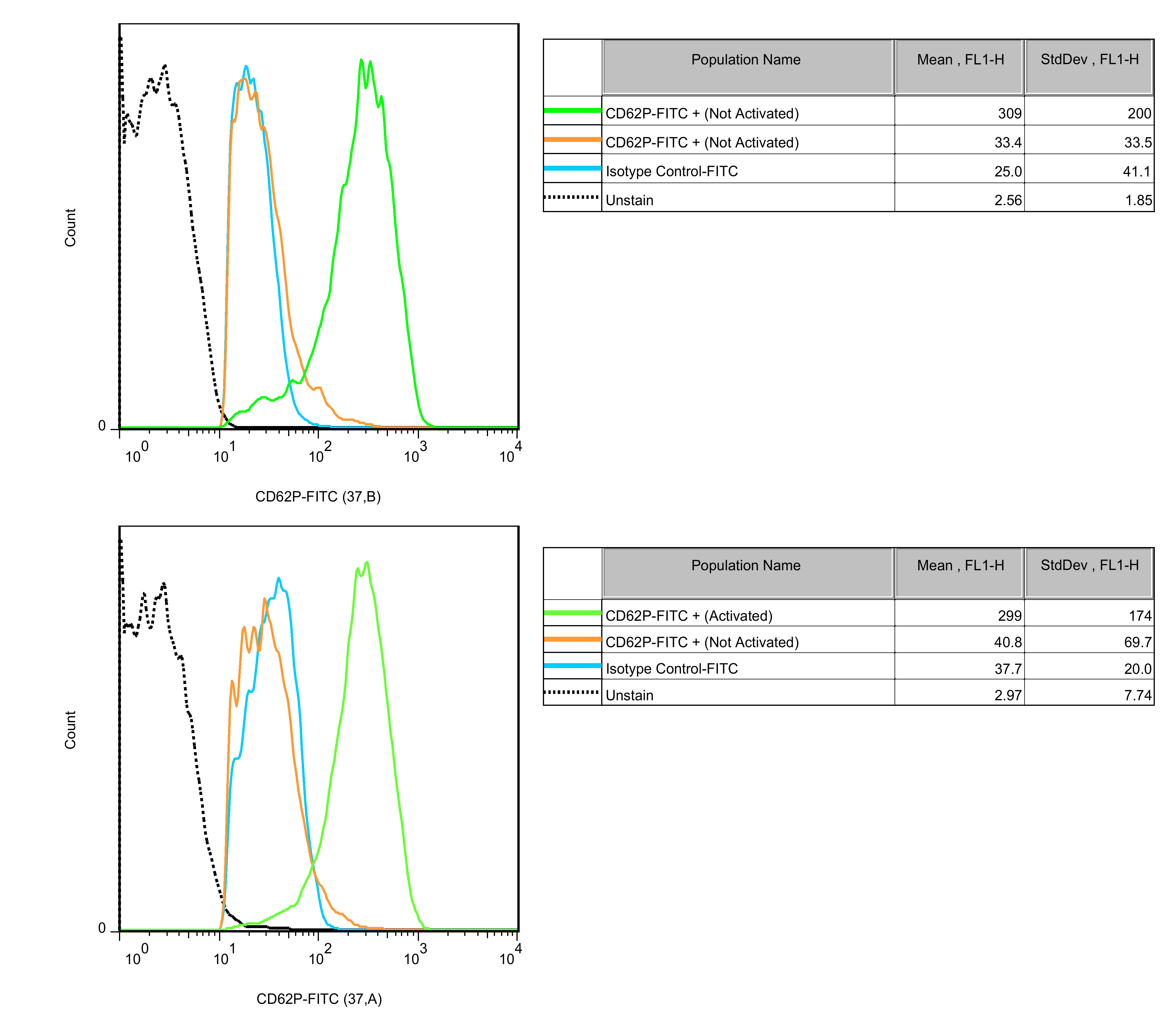

Supplement: Supplementary file 2 — Additional file 2: The Flow Cytometric assay results of per-patient levels of platelet CD62P Ag expression at baseline (B) and week 26 (A). [file 13098_2022_951_MOESM2_ESM.zip › 37.png]

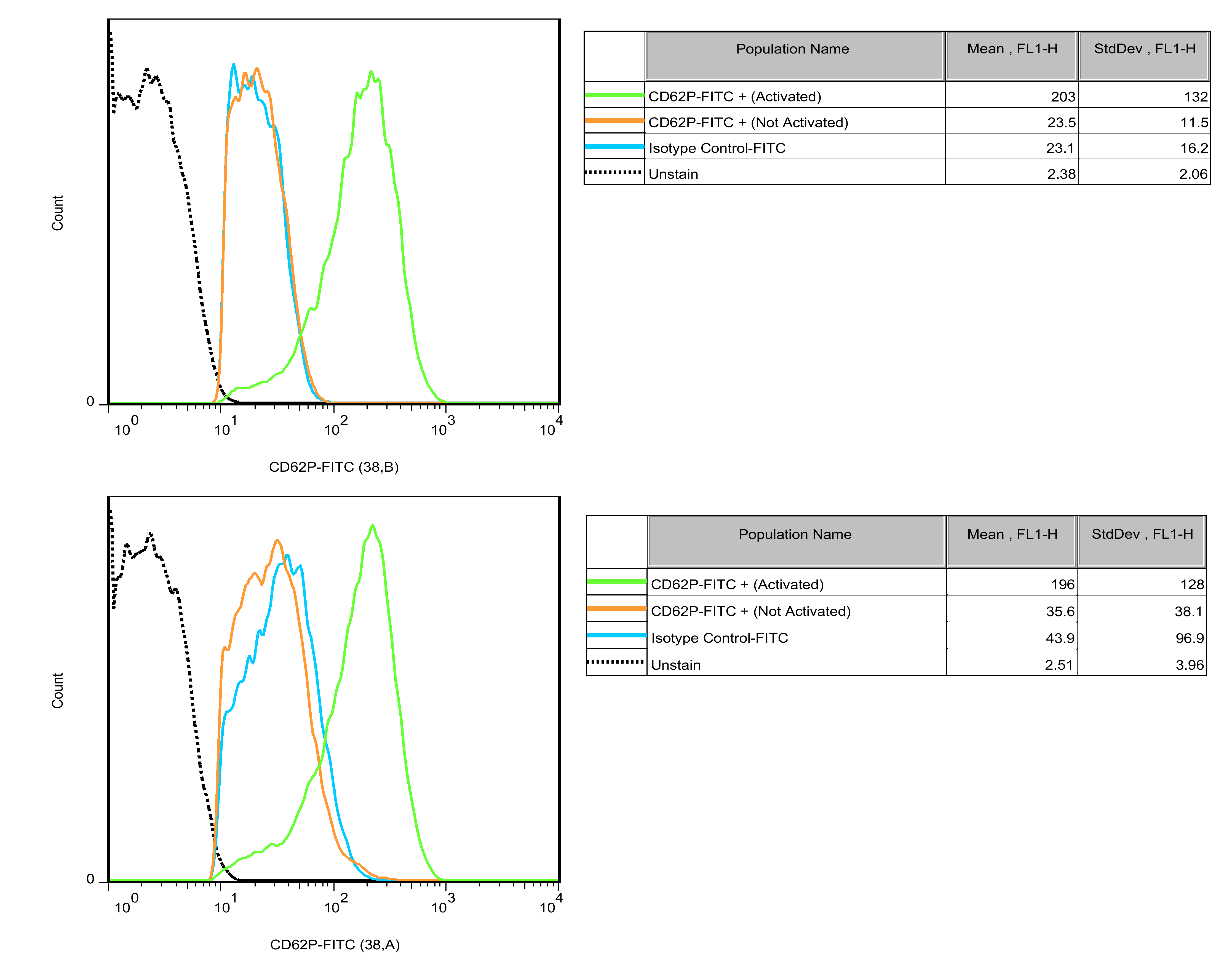

Supplement: Supplementary file 2 — Additional file 2: The Flow Cytometric assay results of per-patient levels of platelet CD62P Ag expression at baseline (B) and week 26 (A). [file 13098_2022_951_MOESM2_ESM.zip › 38.png]

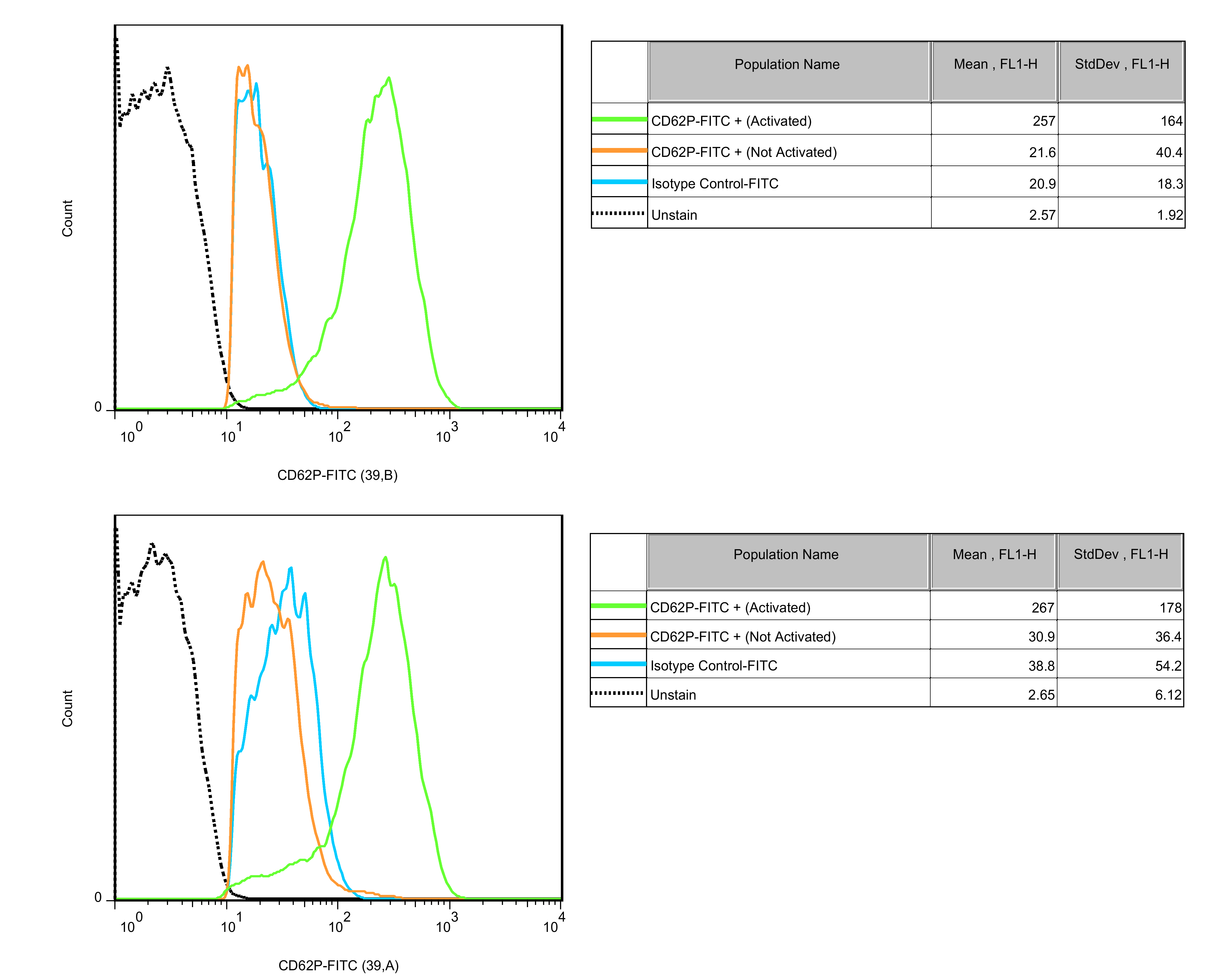

Supplement: Supplementary file 2 — Additional file 2: The Flow Cytometric assay results of per-patient levels of platelet CD62P Ag expression at baseline (B) and week 26 (A). [file 13098_2022_951_MOESM2_ESM.zip › 39.png]

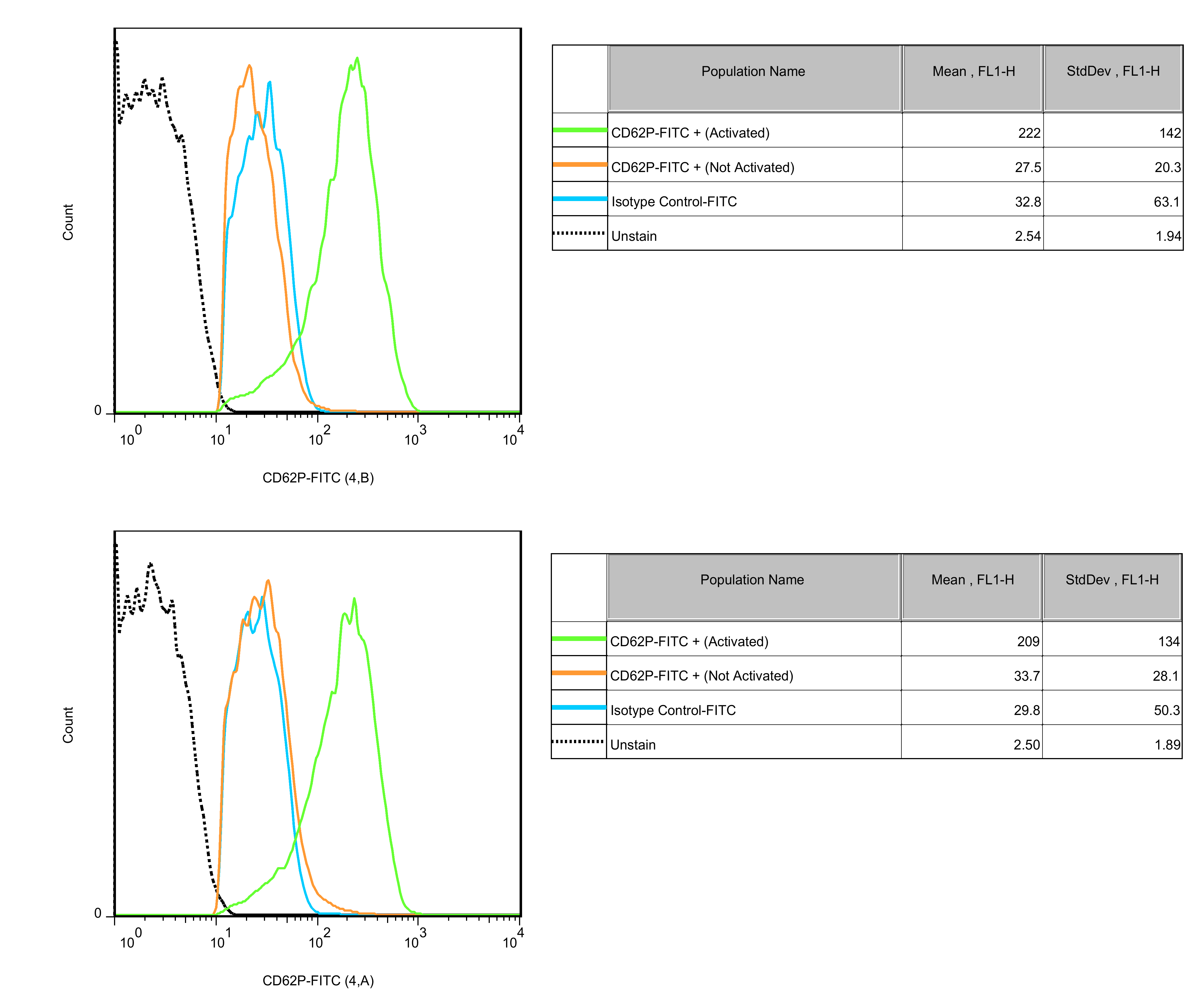

Supplement: Supplementary file 2 — Additional file 2: The Flow Cytometric assay results of per-patient levels of platelet CD62P Ag expression at baseline (B) and week 26 (A). [file 13098_2022_951_MOESM2_ESM.zip › 4.png]

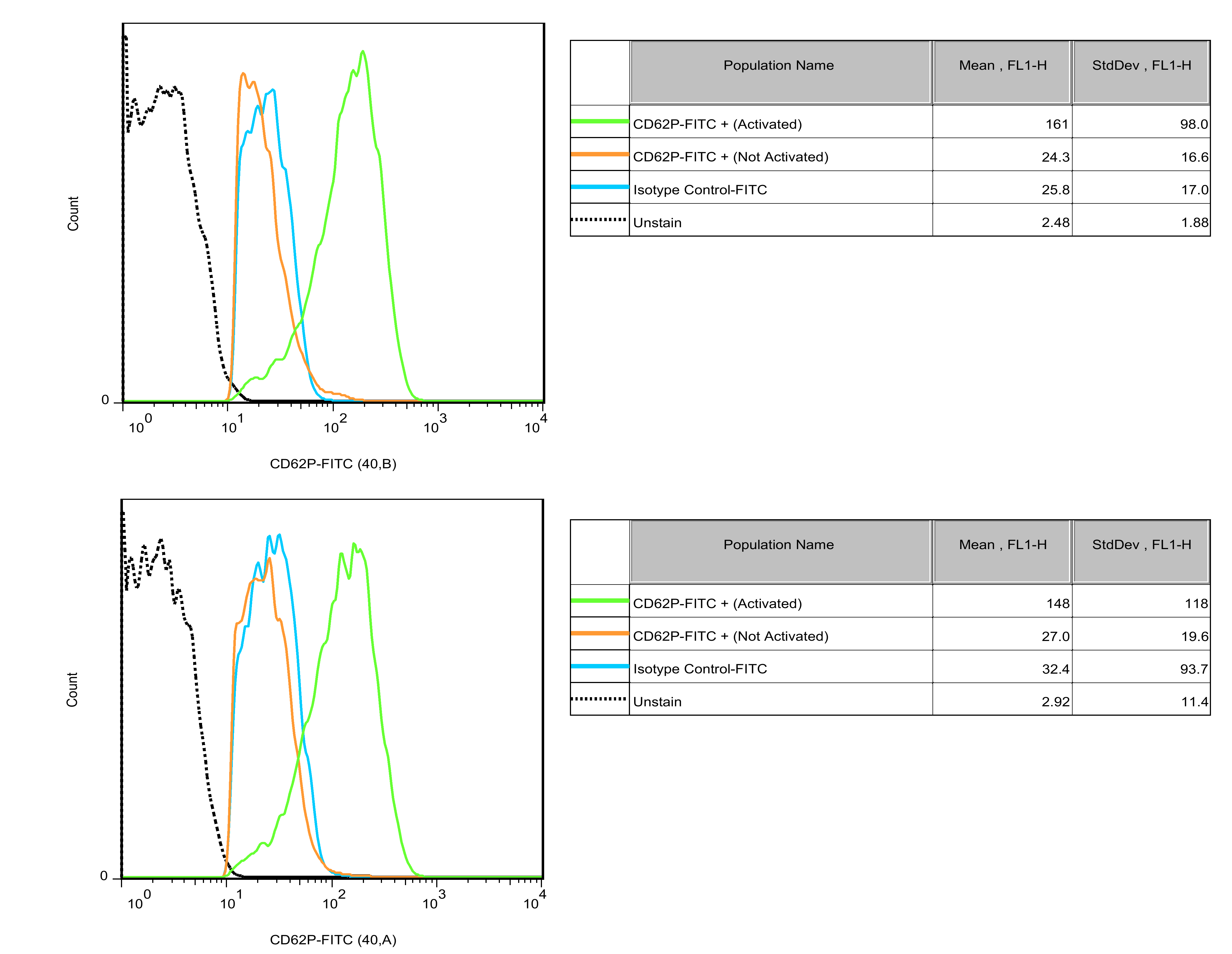

Supplement: Supplementary file 2 — Additional file 2: The Flow Cytometric assay results of per-patient levels of platelet CD62P Ag expression at baseline (B) and week 26 (A). [file 13098_2022_951_MOESM2_ESM.zip › 40.png]

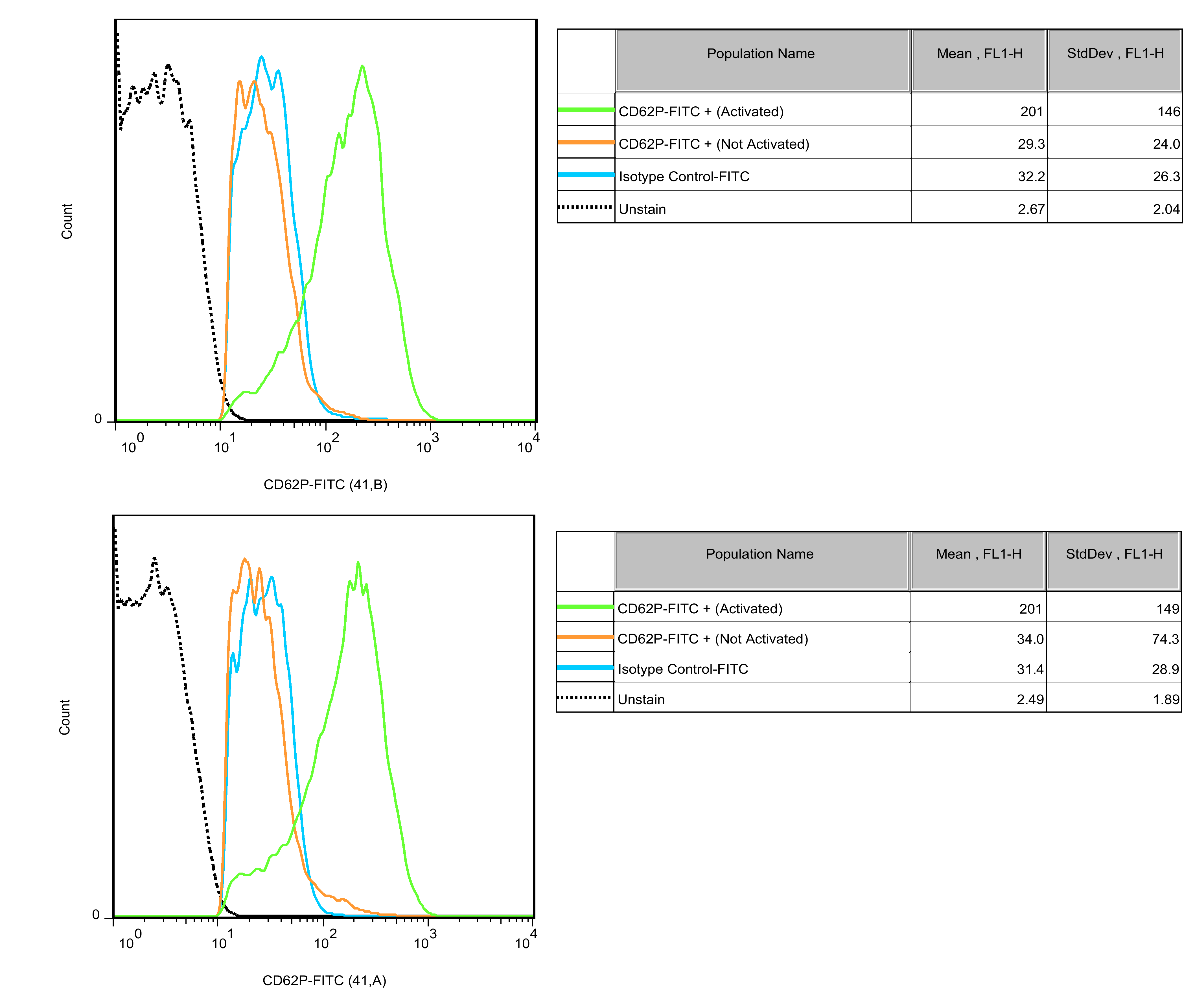

Supplement: Supplementary file 2 — Additional file 2: The Flow Cytometric assay results of per-patient levels of platelet CD62P Ag expression at baseline (B) and week 26 (A). [file 13098_2022_951_MOESM2_ESM.zip › 41.png]

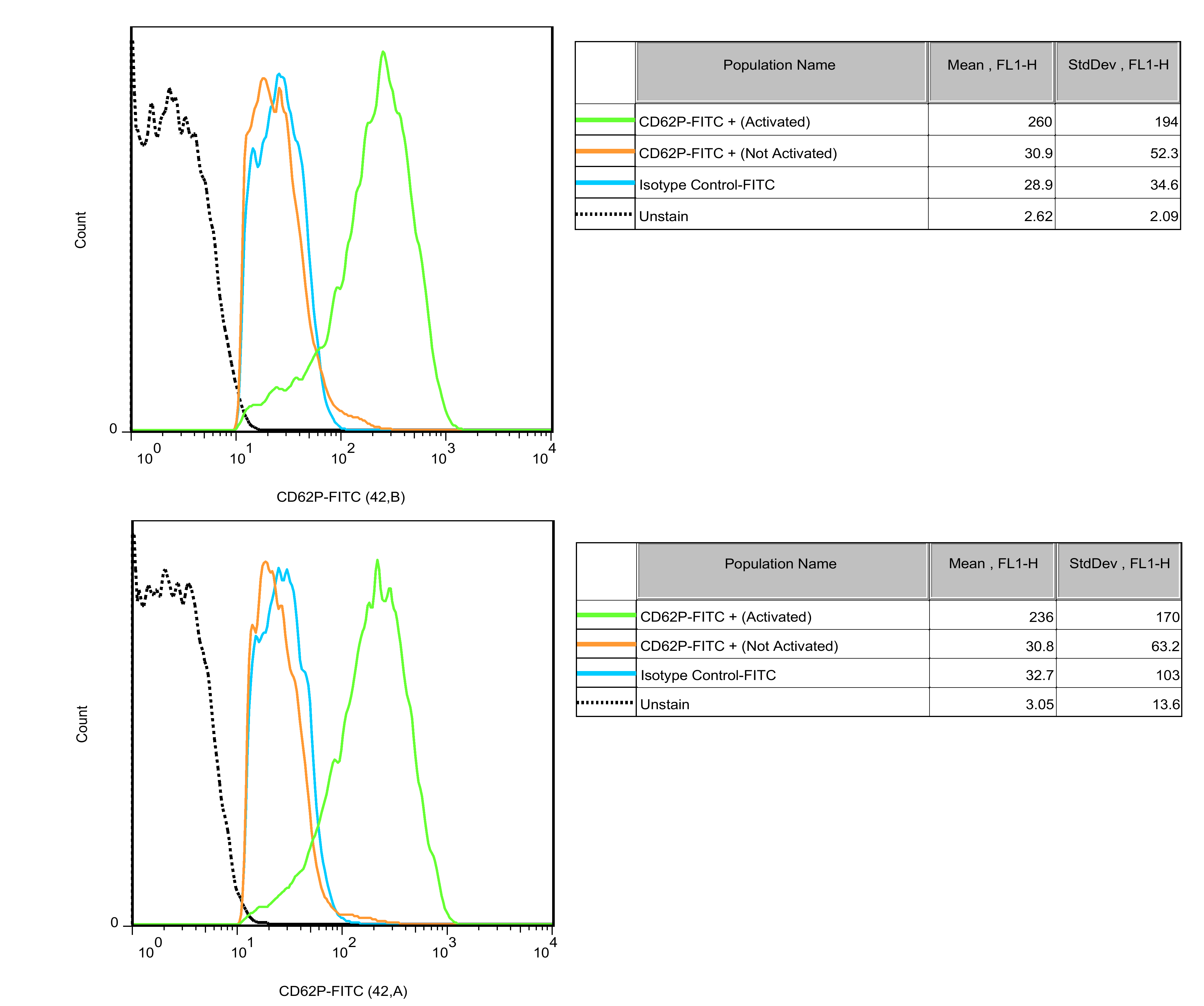

Supplement: Supplementary file 2 — Additional file 2: The Flow Cytometric assay results of per-patient levels of platelet CD62P Ag expression at baseline (B) and week 26 (A). [file 13098_2022_951_MOESM2_ESM.zip › 42.png]

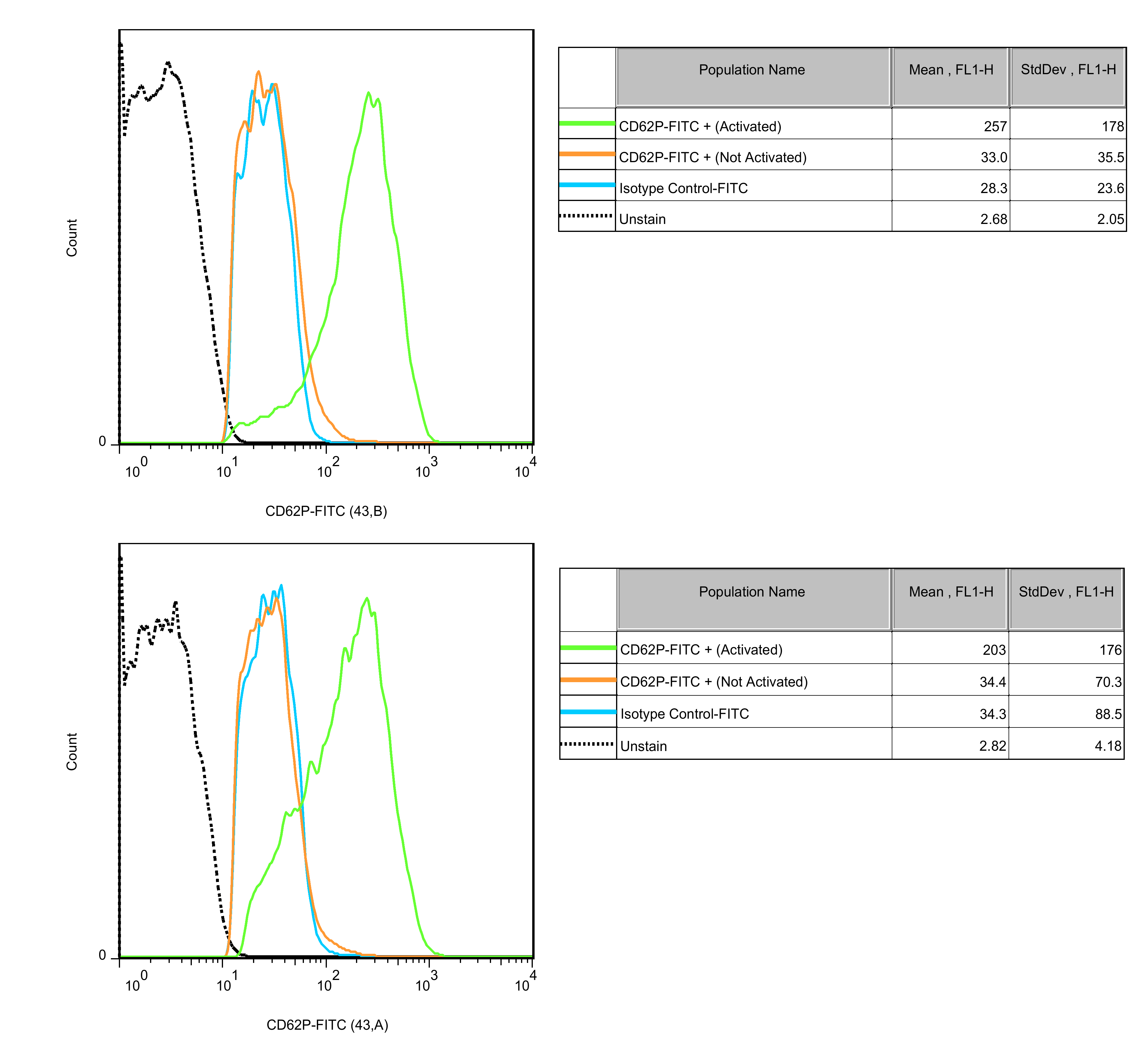

Supplement: Supplementary file 2 — Additional file 2: The Flow Cytometric assay results of per-patient levels of platelet CD62P Ag expression at baseline (B) and week 26 (A). [file 13098_2022_951_MOESM2_ESM.zip › 43.png]

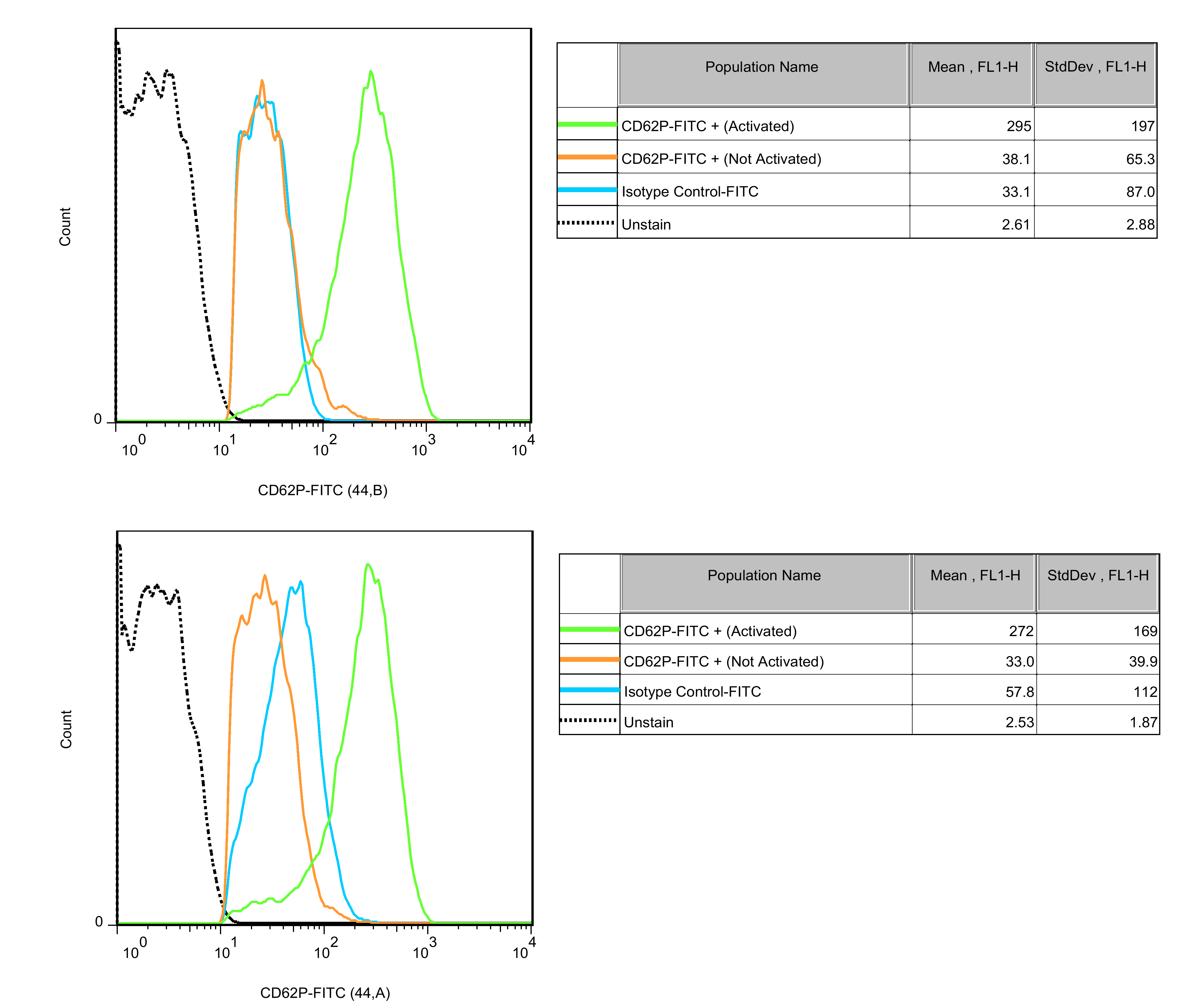

Supplement: Supplementary file 2 — Additional file 2: The Flow Cytometric assay results of per-patient levels of platelet CD62P Ag expression at baseline (B) and week 26 (A). [file 13098_2022_951_MOESM2_ESM.zip › 44.png]

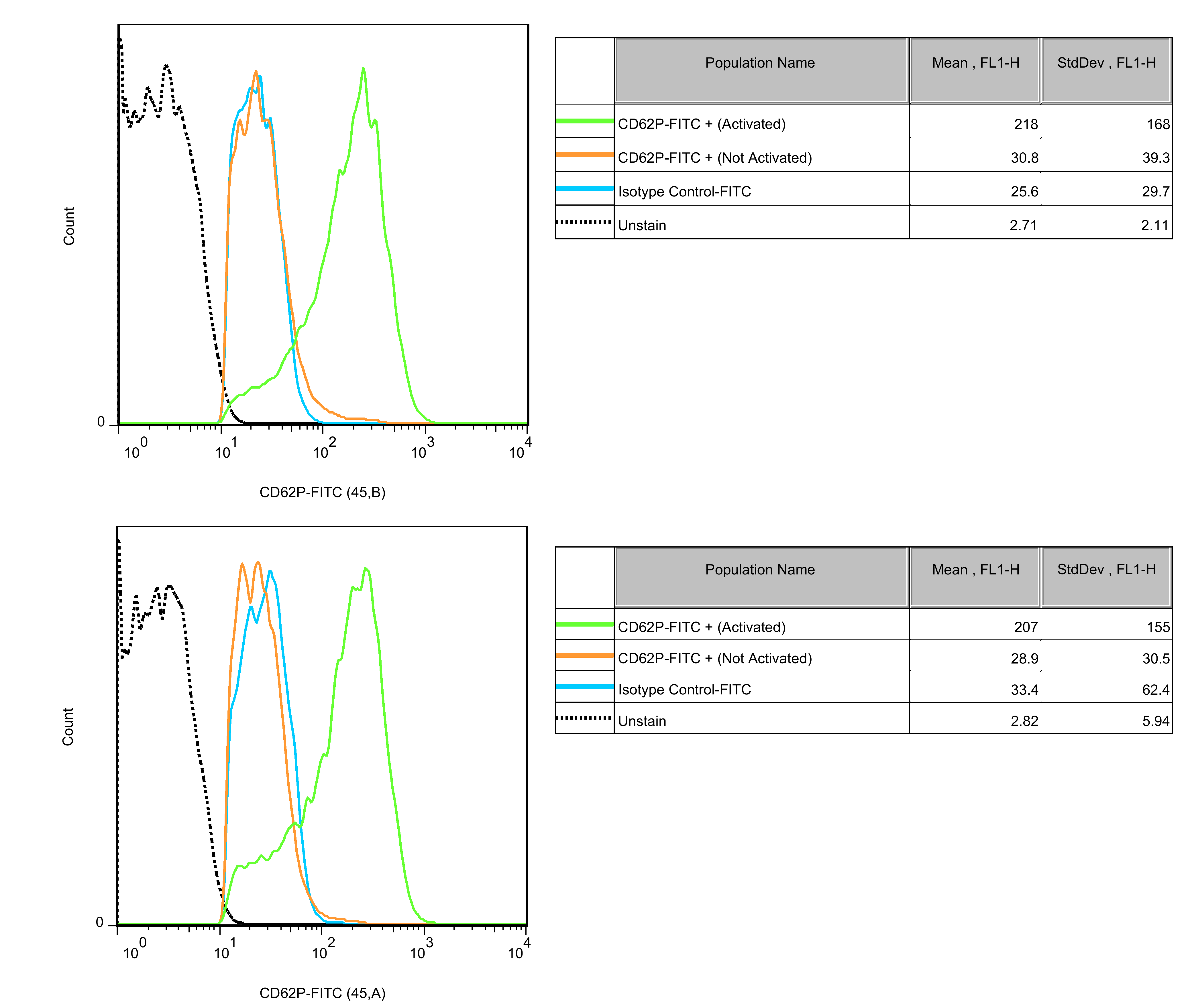

Supplement: Supplementary file 2 — Additional file 2: The Flow Cytometric assay results of per-patient levels of platelet CD62P Ag expression at baseline (B) and week 26 (A). [file 13098_2022_951_MOESM2_ESM.zip › 45.png]

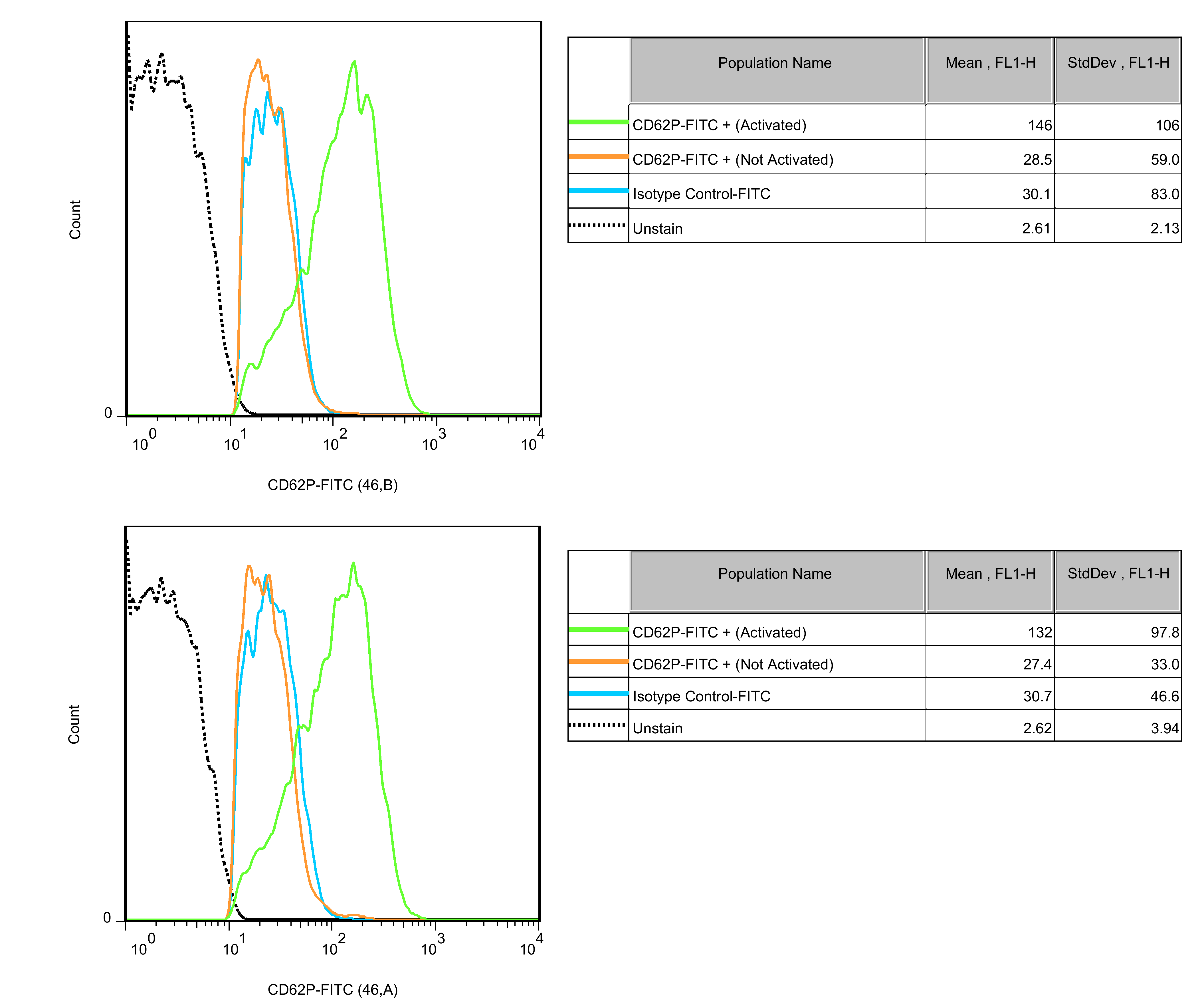

Supplement: Supplementary file 2 — Additional file 2: The Flow Cytometric assay results of per-patient levels of platelet CD62P Ag expression at baseline (B) and week 26 (A). [file 13098_2022_951_MOESM2_ESM.zip › 46.png]

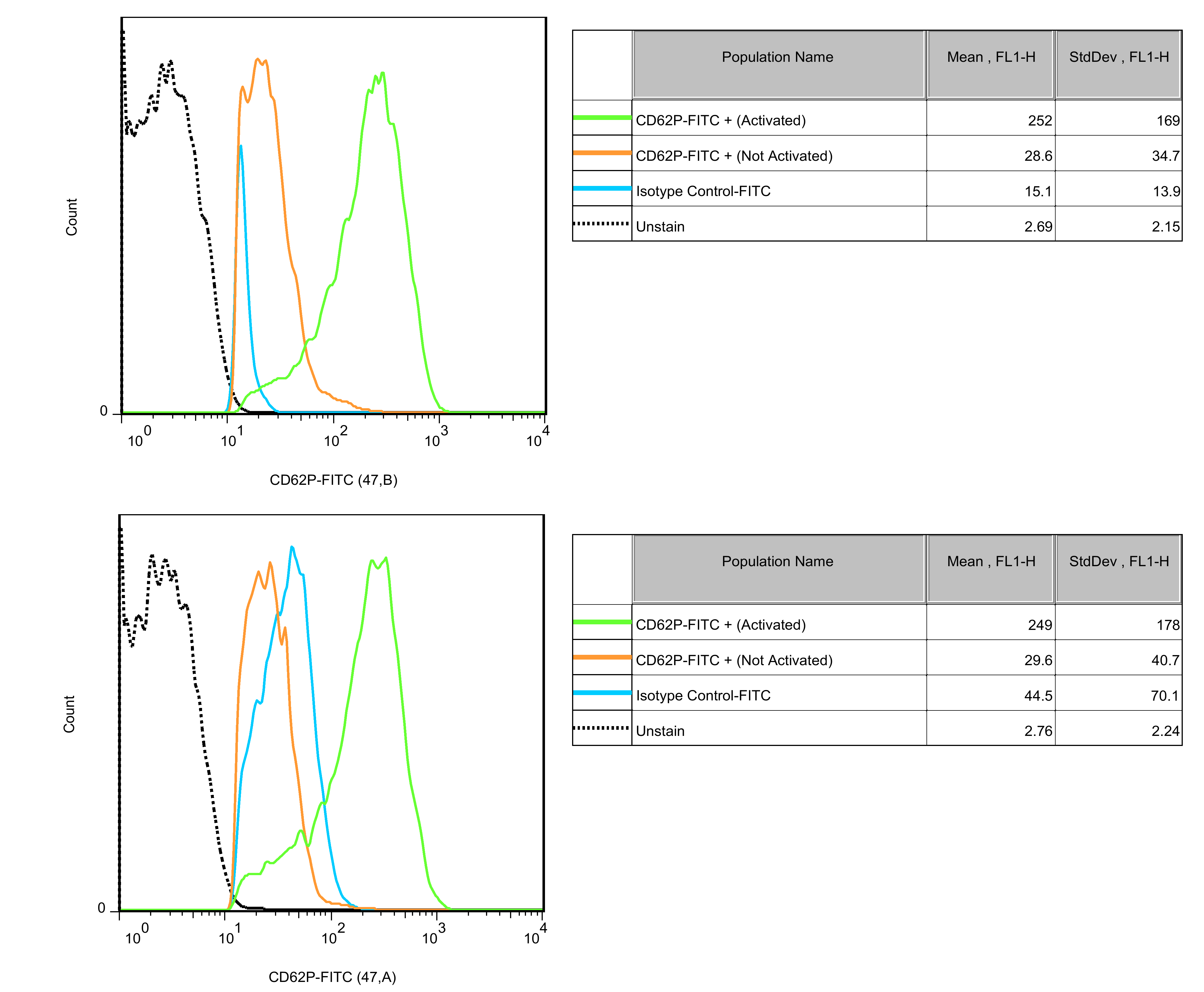

Supplement: Supplementary file 2 — Additional file 2: The Flow Cytometric assay results of per-patient levels of platelet CD62P Ag expression at baseline (B) and week 26 (A). [file 13098_2022_951_MOESM2_ESM.zip › 47.png]

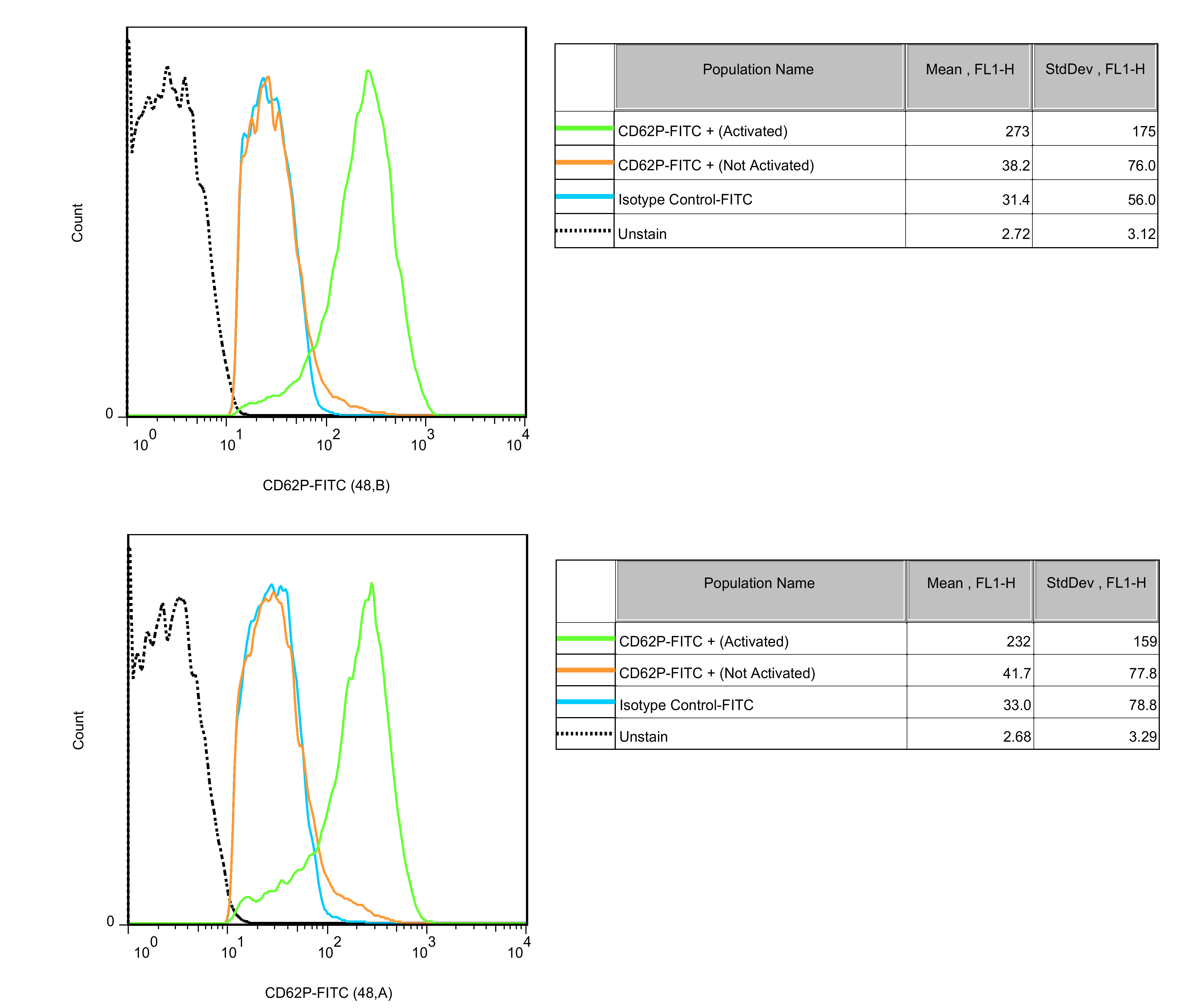

Supplement: Supplementary file 2 — Additional file 2: The Flow Cytometric assay results of per-patient levels of platelet CD62P Ag expression at baseline (B) and week 26 (A). [file 13098_2022_951_MOESM2_ESM.zip › 48.png]

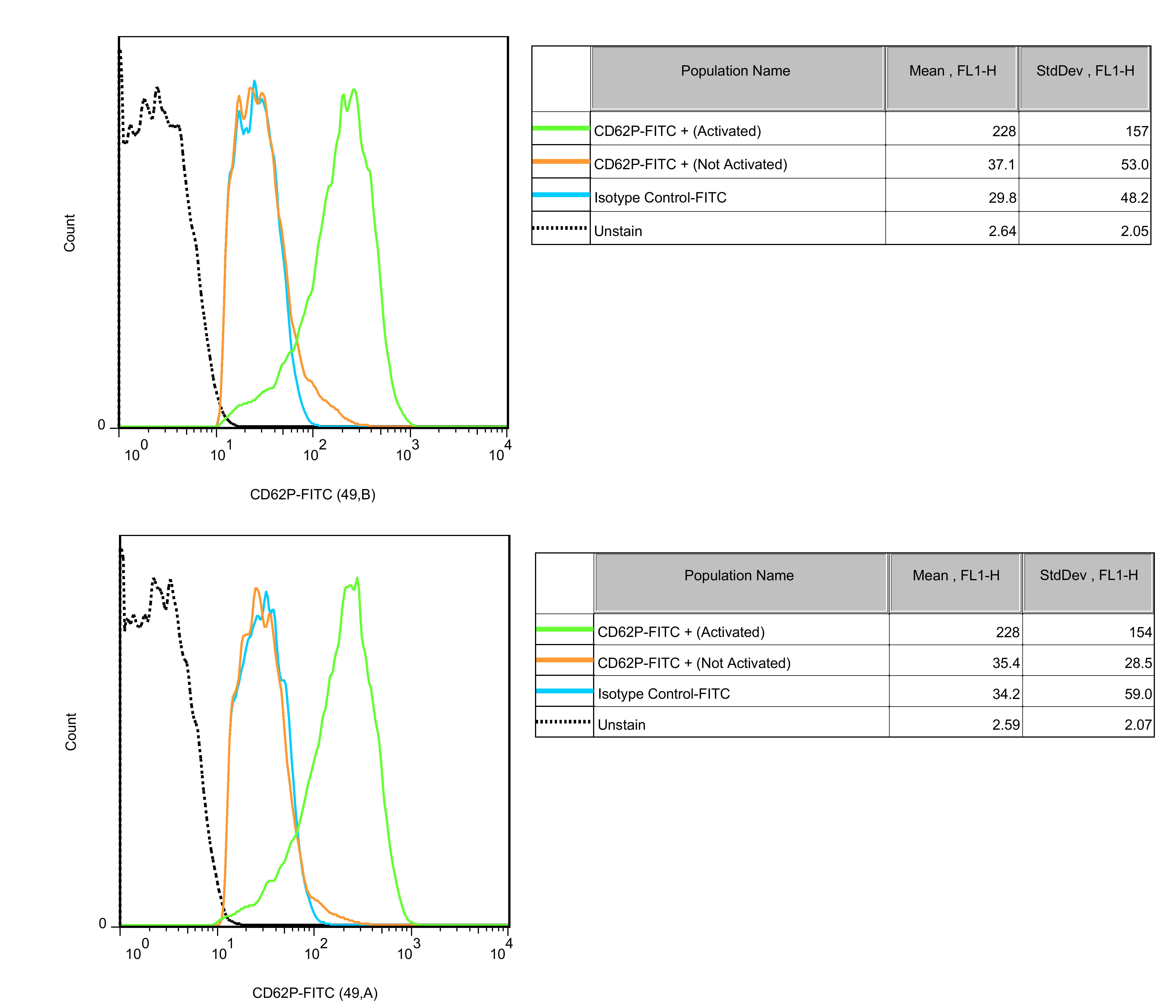

Supplement: Supplementary file 2 — Additional file 2: The Flow Cytometric assay results of per-patient levels of platelet CD62P Ag expression at baseline (B) and week 26 (A). [file 13098_2022_951_MOESM2_ESM.zip › 49.png]

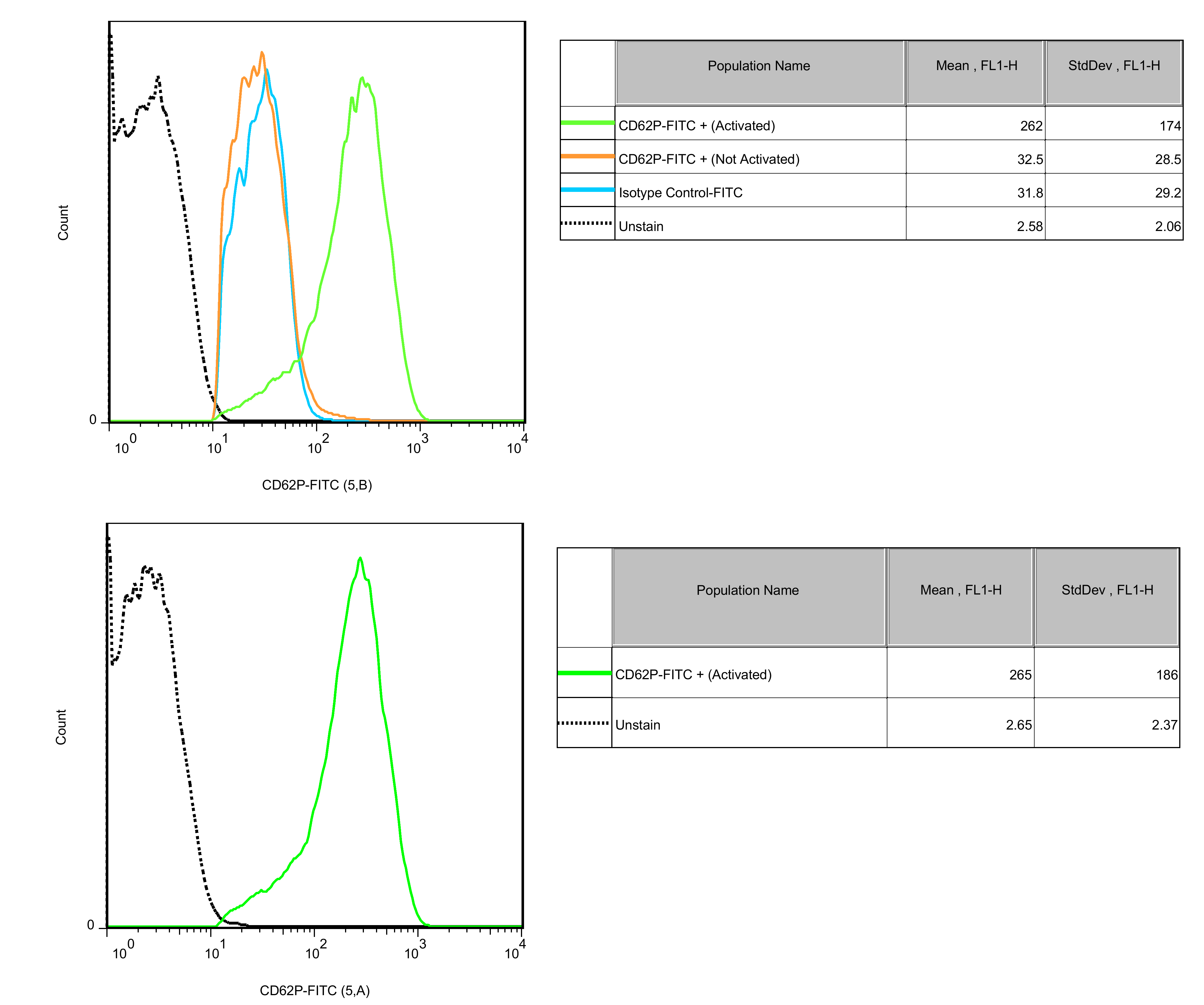

Supplement: Supplementary file 2 — Additional file 2: The Flow Cytometric assay results of per-patient levels of platelet CD62P Ag expression at baseline (B) and week 26 (A). [file 13098_2022_951_MOESM2_ESM.zip › 5.png]

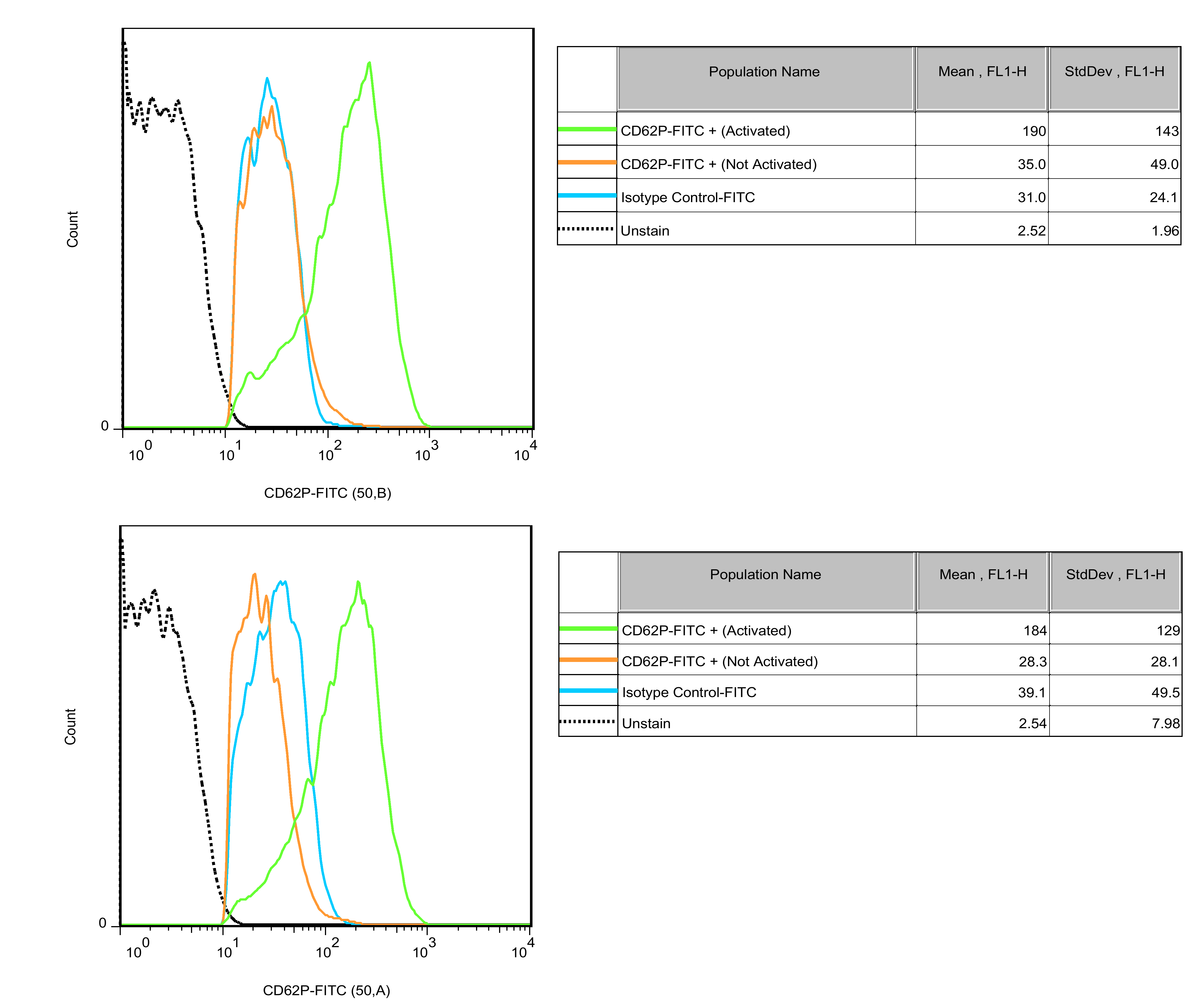

Supplement: Supplementary file 2 — Additional file 2: The Flow Cytometric assay results of per-patient levels of platelet CD62P Ag expression at baseline (B) and week 26 (A). [file 13098_2022_951_MOESM2_ESM.zip › 50.png]

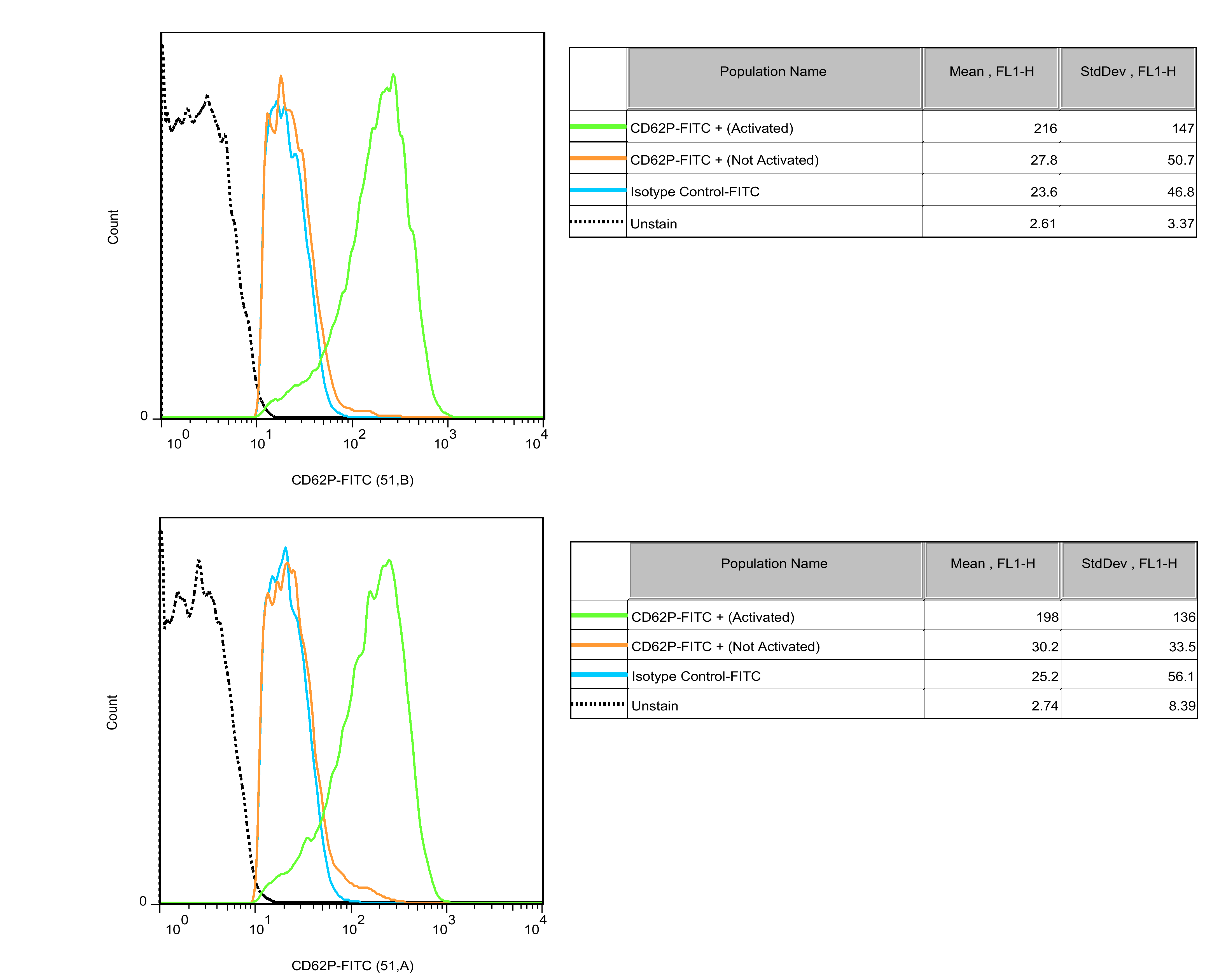

Supplement: Supplementary file 2 — Additional file 2: The Flow Cytometric assay results of per-patient levels of platelet CD62P Ag expression at baseline (B) and week 26 (A). [file 13098_2022_951_MOESM2_ESM.zip › 51.png]

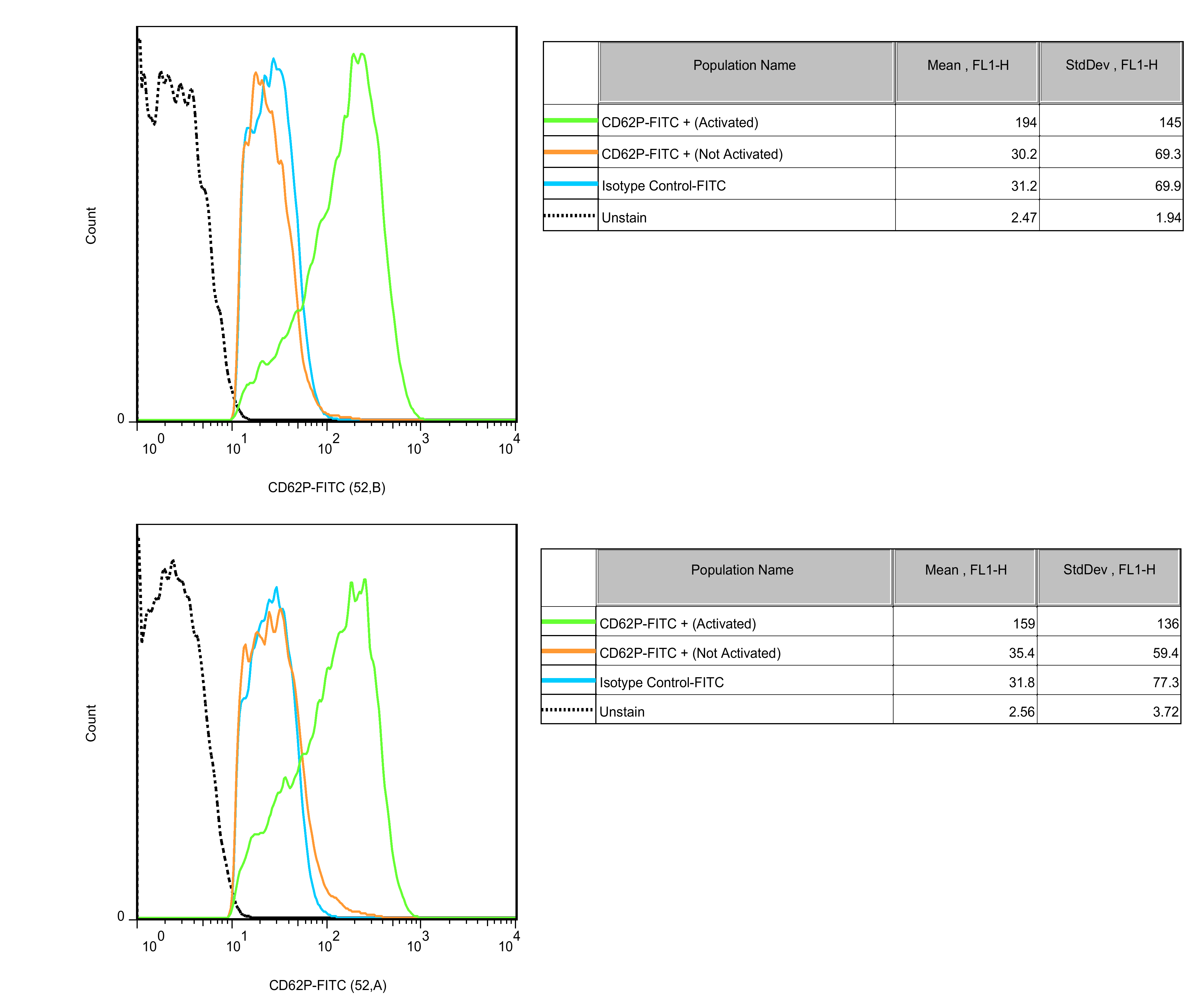

Supplement: Supplementary file 2 — Additional file 2: The Flow Cytometric assay results of per-patient levels of platelet CD62P Ag expression at baseline (B) and week 26 (A). [file 13098_2022_951_MOESM2_ESM.zip › 52.png]

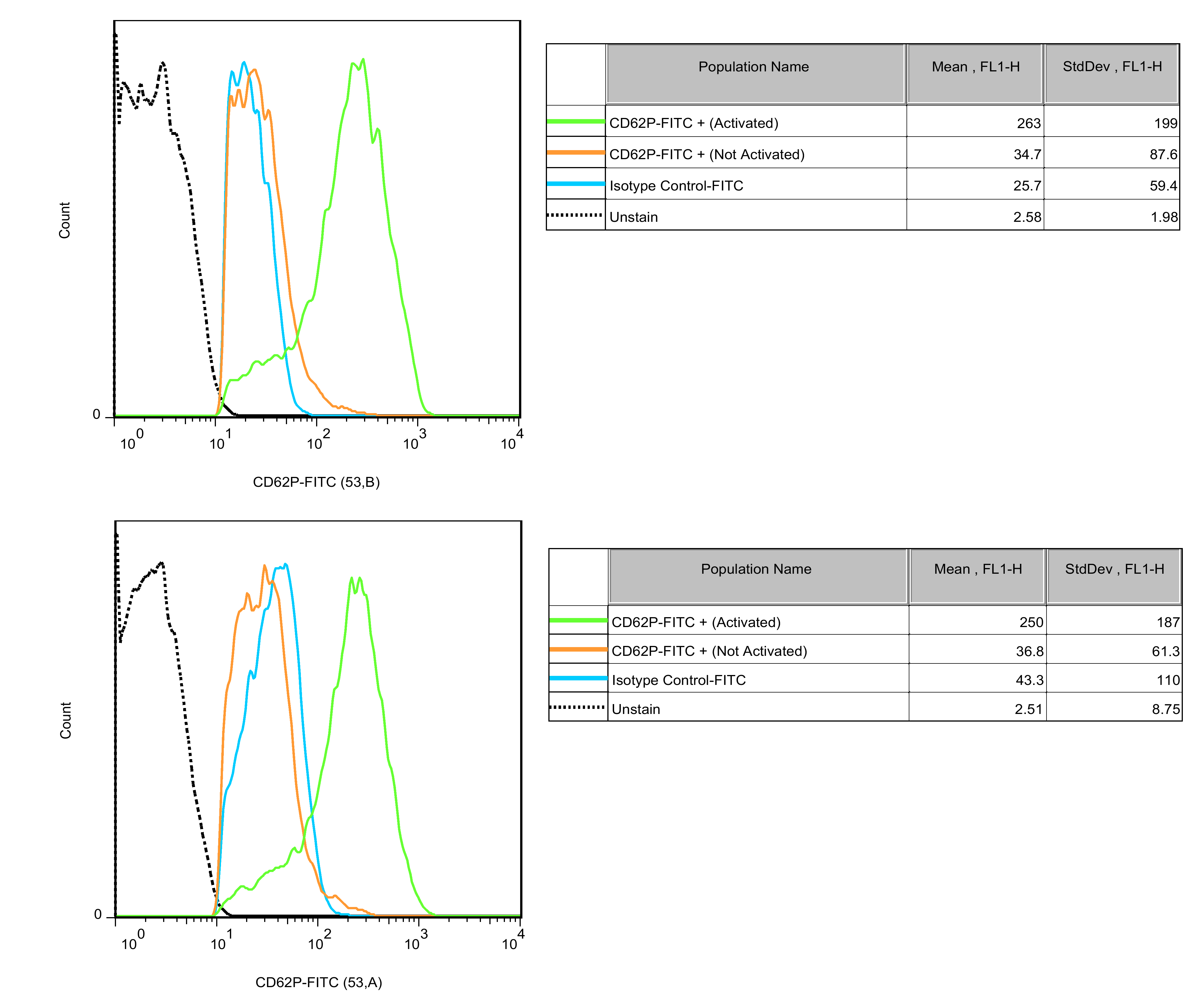

Supplement: Supplementary file 2 — Additional file 2: The Flow Cytometric assay results of per-patient levels of platelet CD62P Ag expression at baseline (B) and week 26 (A). [file 13098_2022_951_MOESM2_ESM.zip › 53.png]

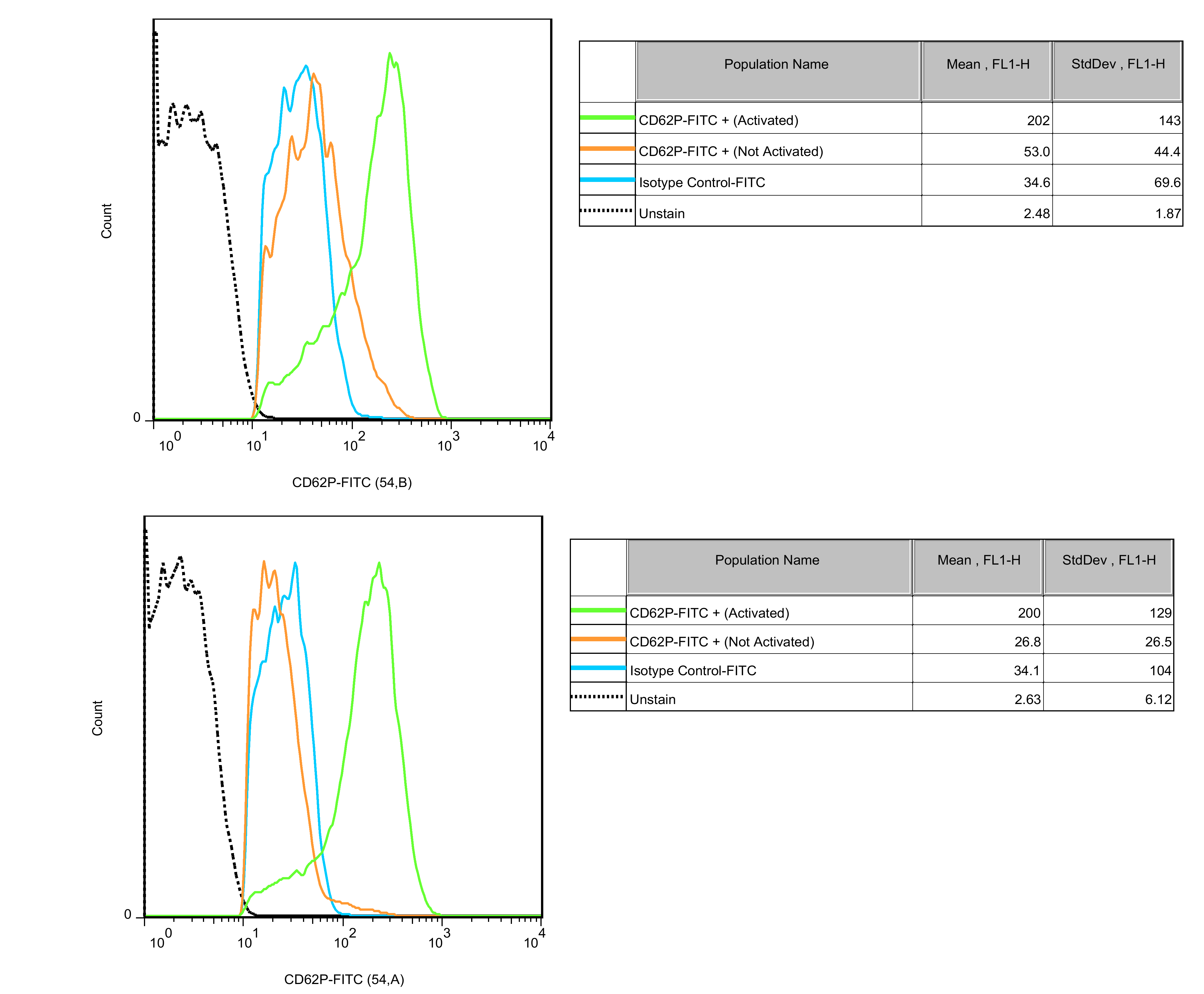

Supplement: Supplementary file 2 — Additional file 2: The Flow Cytometric assay results of per-patient levels of platelet CD62P Ag expression at baseline (B) and week 26 (A). [file 13098_2022_951_MOESM2_ESM.zip › 54.png]

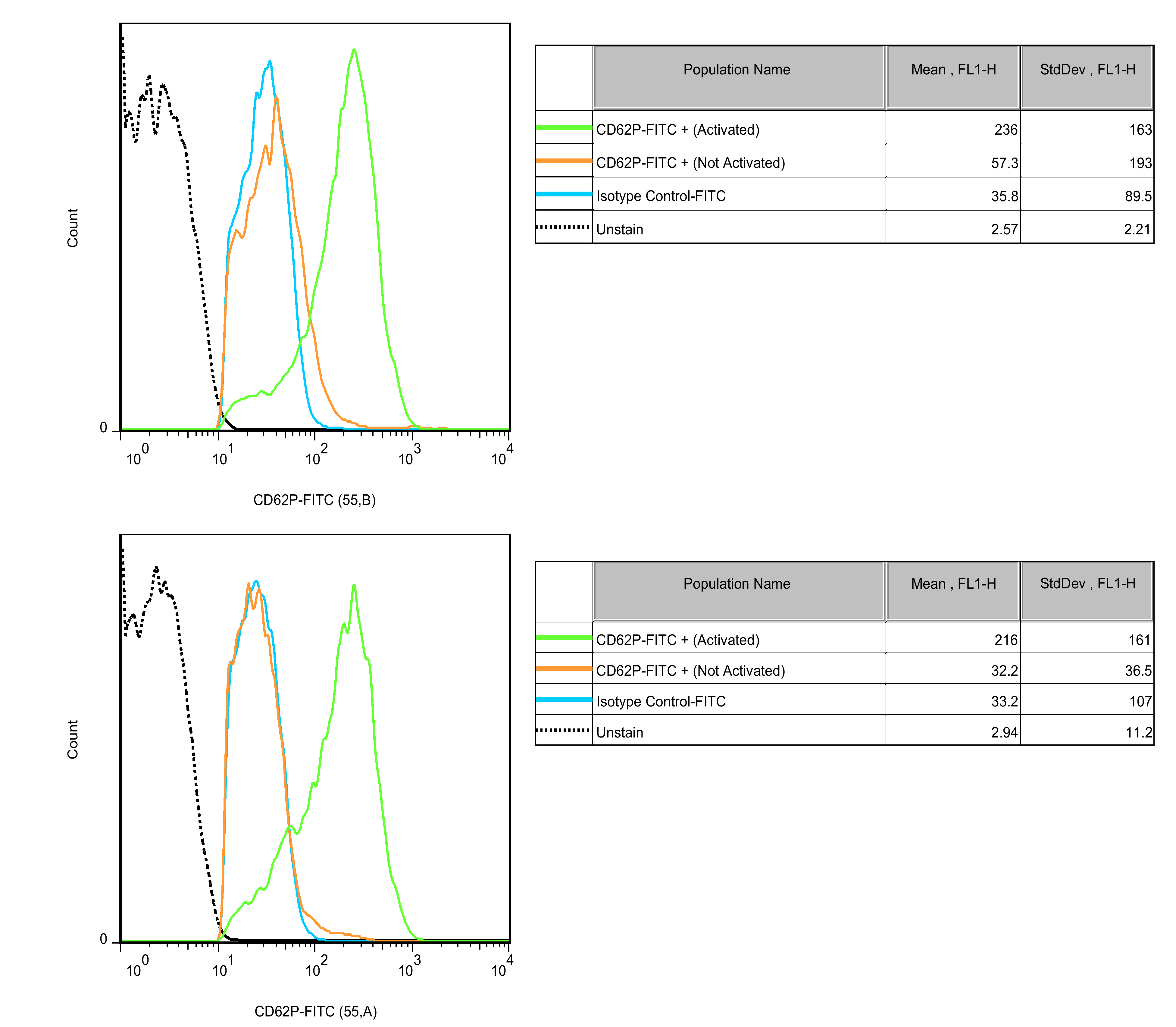

Supplement: Supplementary file 2 — Additional file 2: The Flow Cytometric assay results of per-patient levels of platelet CD62P Ag expression at baseline (B) and week 26 (A). [file 13098_2022_951_MOESM2_ESM.zip › 55.png]

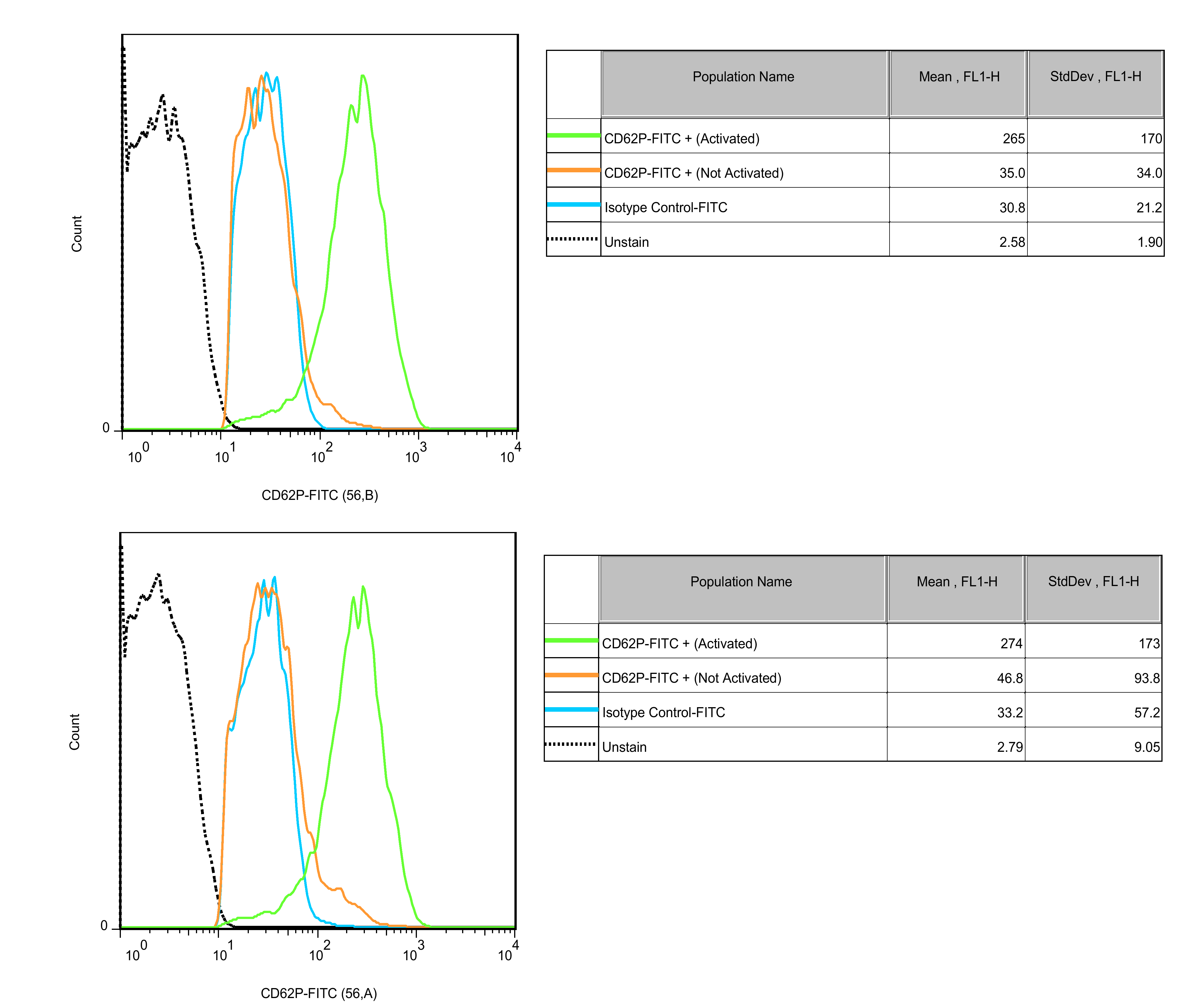

Supplement: Supplementary file 2 — Additional file 2: The Flow Cytometric assay results of per-patient levels of platelet CD62P Ag expression at baseline (B) and week 26 (A). [file 13098_2022_951_MOESM2_ESM.zip › 56.png]

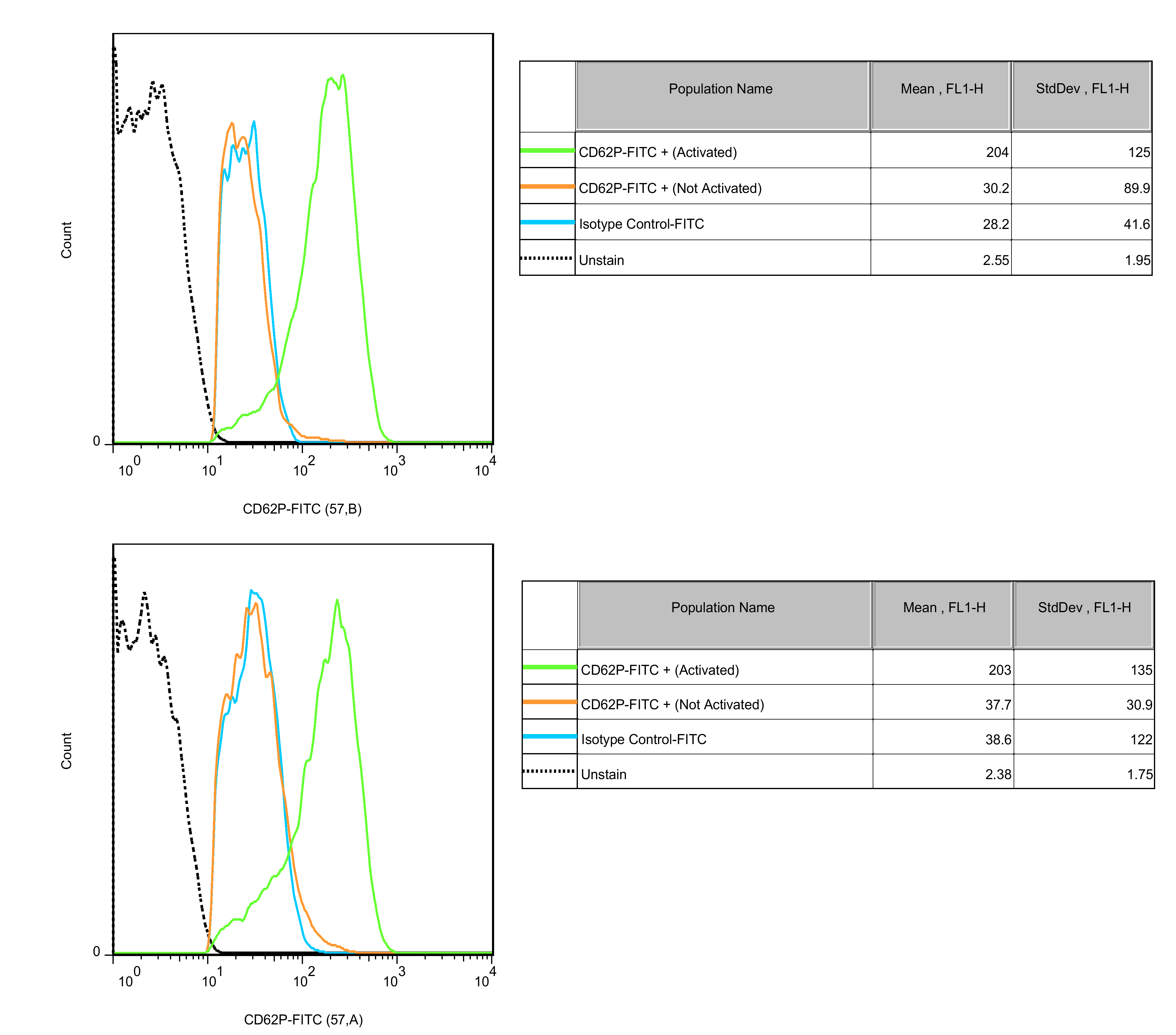

Supplement: Supplementary file 2 — Additional file 2: The Flow Cytometric assay results of per-patient levels of platelet CD62P Ag expression at baseline (B) and week 26 (A). [file 13098_2022_951_MOESM2_ESM.zip › 57.png]

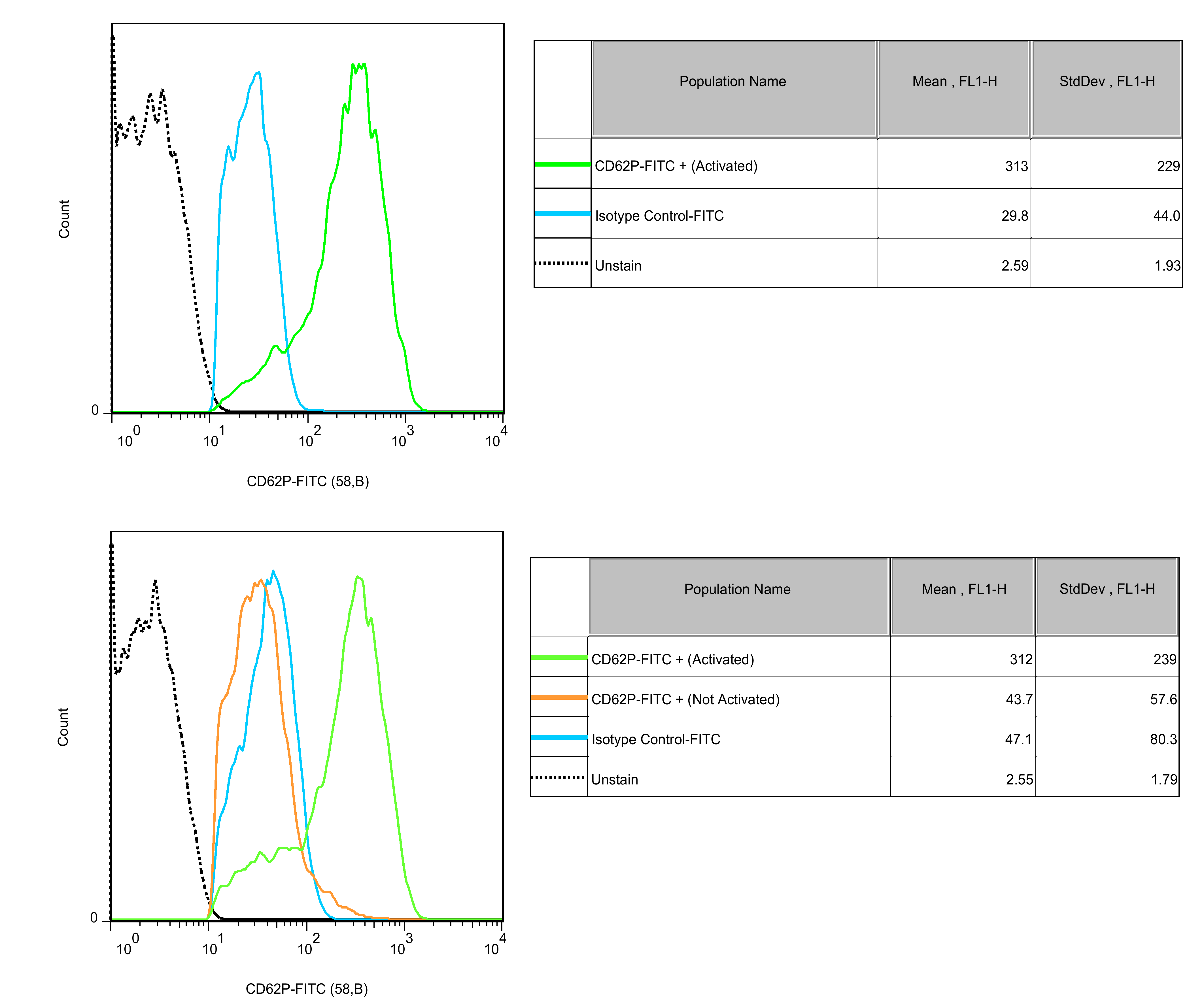

Supplement: Supplementary file 2 — Additional file 2: The Flow Cytometric assay results of per-patient levels of platelet CD62P Ag expression at baseline (B) and week 26 (A). [file 13098_2022_951_MOESM2_ESM.zip › 58.png]

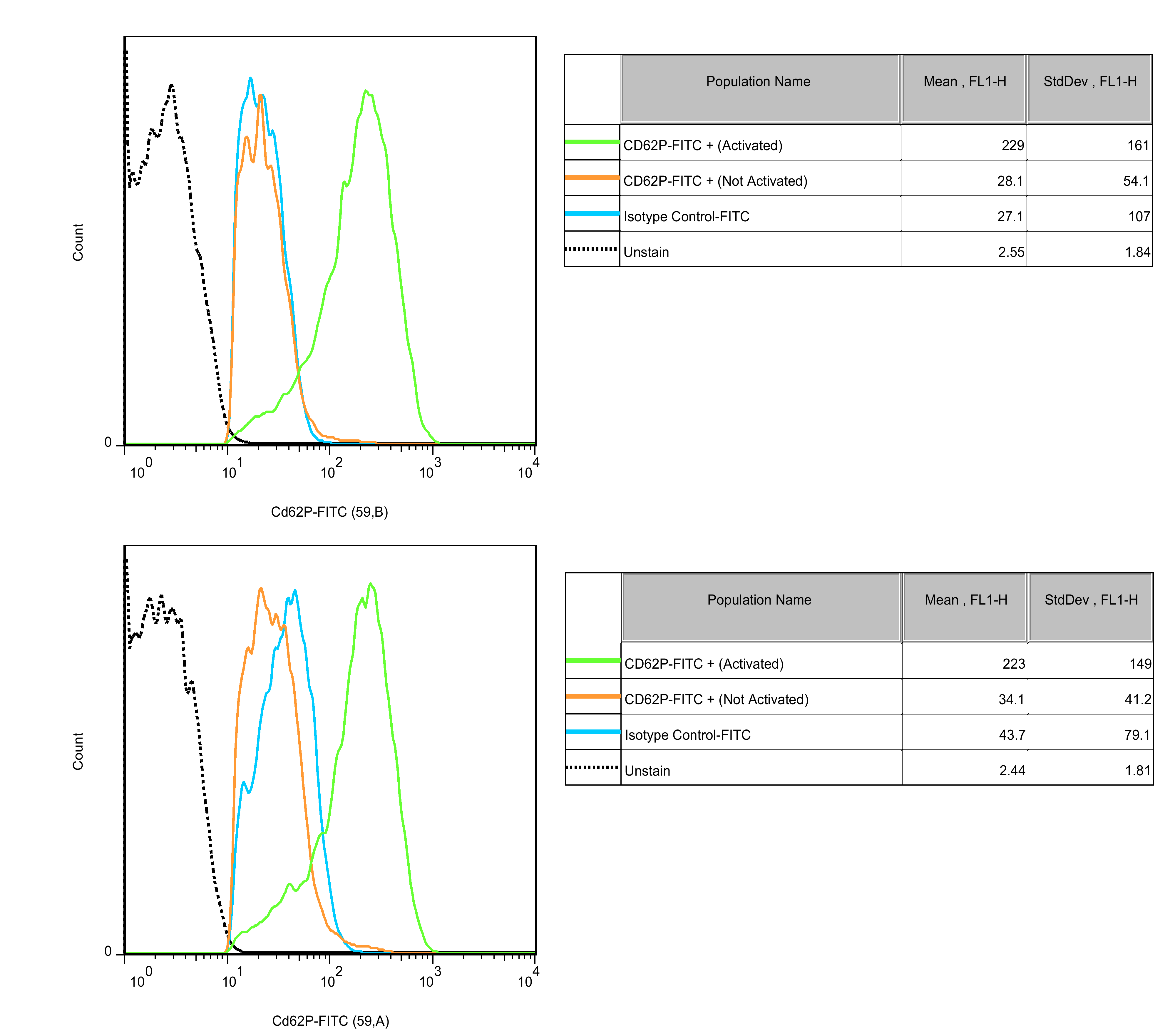

Supplement: Supplementary file 2 — Additional file 2: The Flow Cytometric assay results of per-patient levels of platelet CD62P Ag expression at baseline (B) and week 26 (A). [file 13098_2022_951_MOESM2_ESM.zip › 59.png]

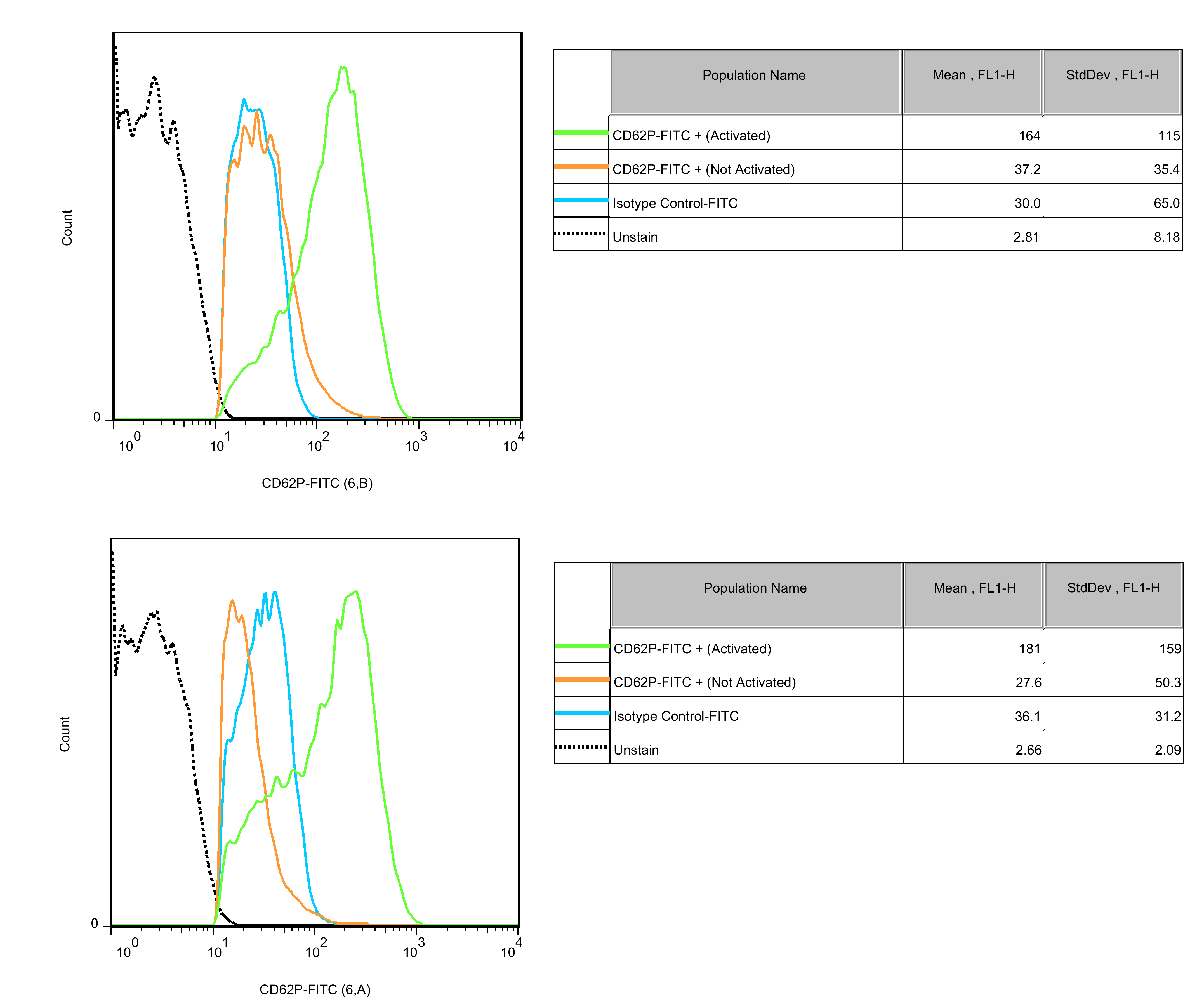

Supplement: Supplementary file 2 — Additional file 2: The Flow Cytometric assay results of per-patient levels of platelet CD62P Ag expression at baseline (B) and week 26 (A). [file 13098_2022_951_MOESM2_ESM.zip › 6.png]

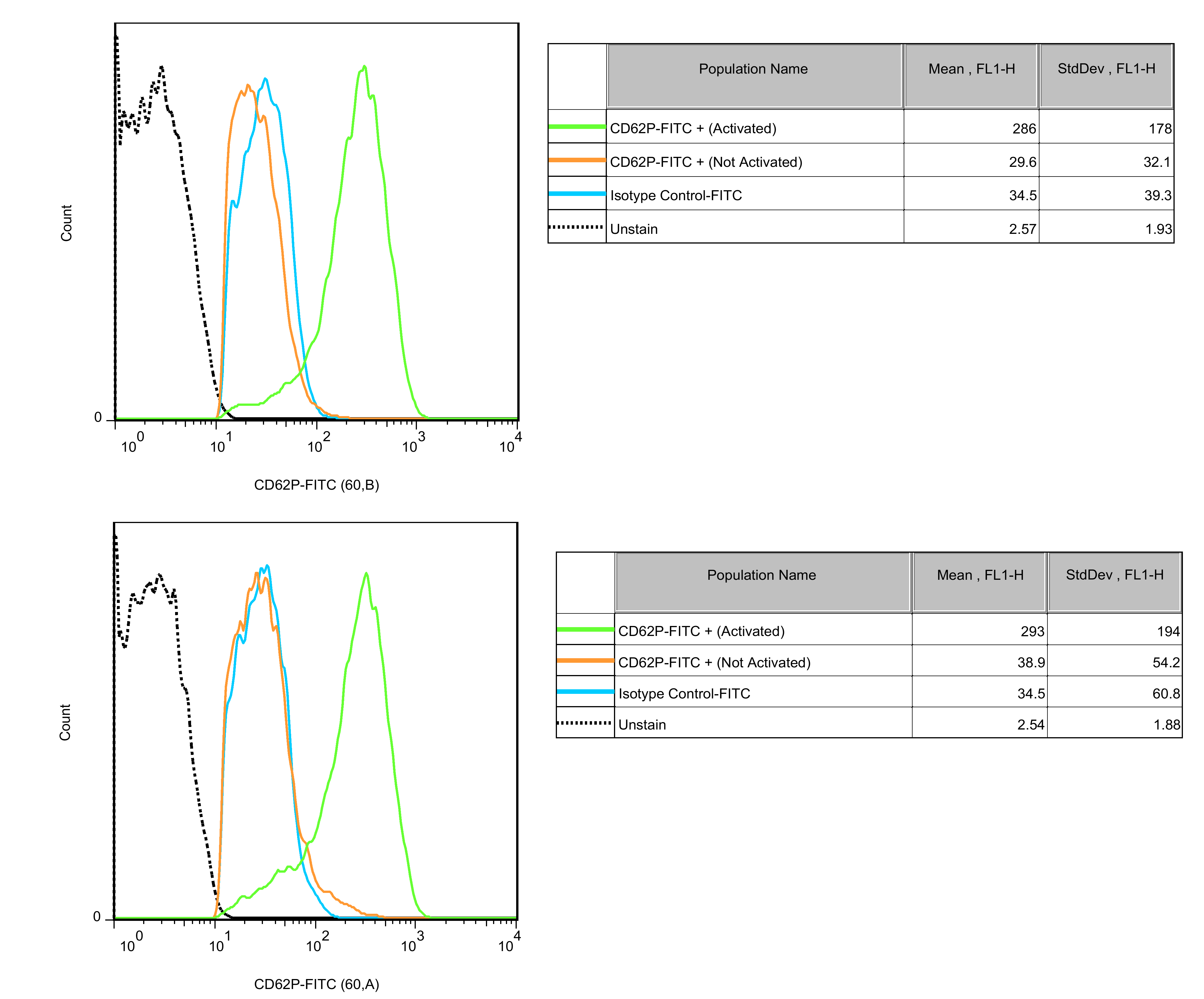

Supplement: Supplementary file 2 — Additional file 2: The Flow Cytometric assay results of per-patient levels of platelet CD62P Ag expression at baseline (B) and week 26 (A). [file 13098_2022_951_MOESM2_ESM.zip › 60.png]

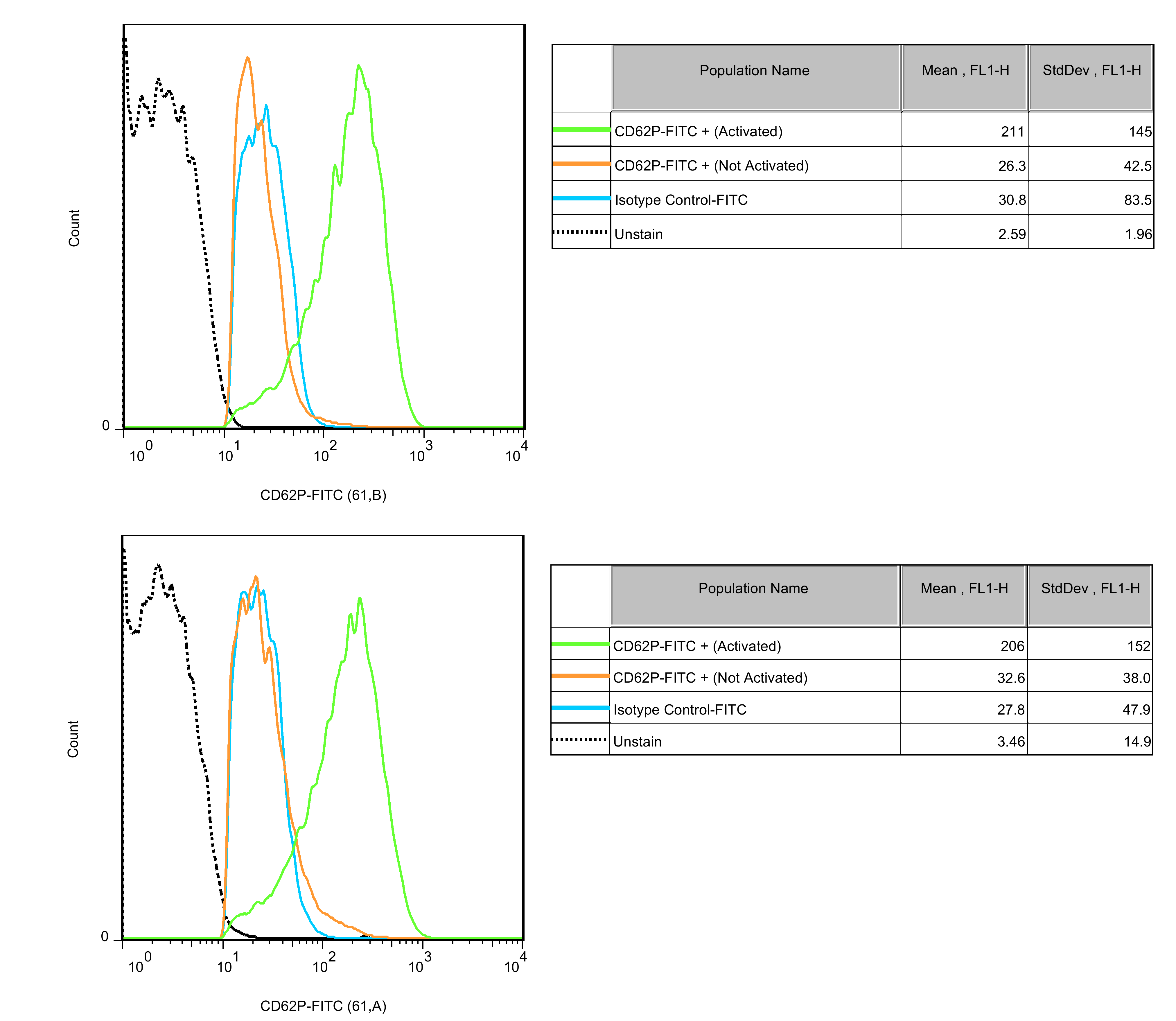

Supplement: Supplementary file 2 — Additional file 2: The Flow Cytometric assay results of per-patient levels of platelet CD62P Ag expression at baseline (B) and week 26 (A). [file 13098_2022_951_MOESM2_ESM.zip › 61.png]

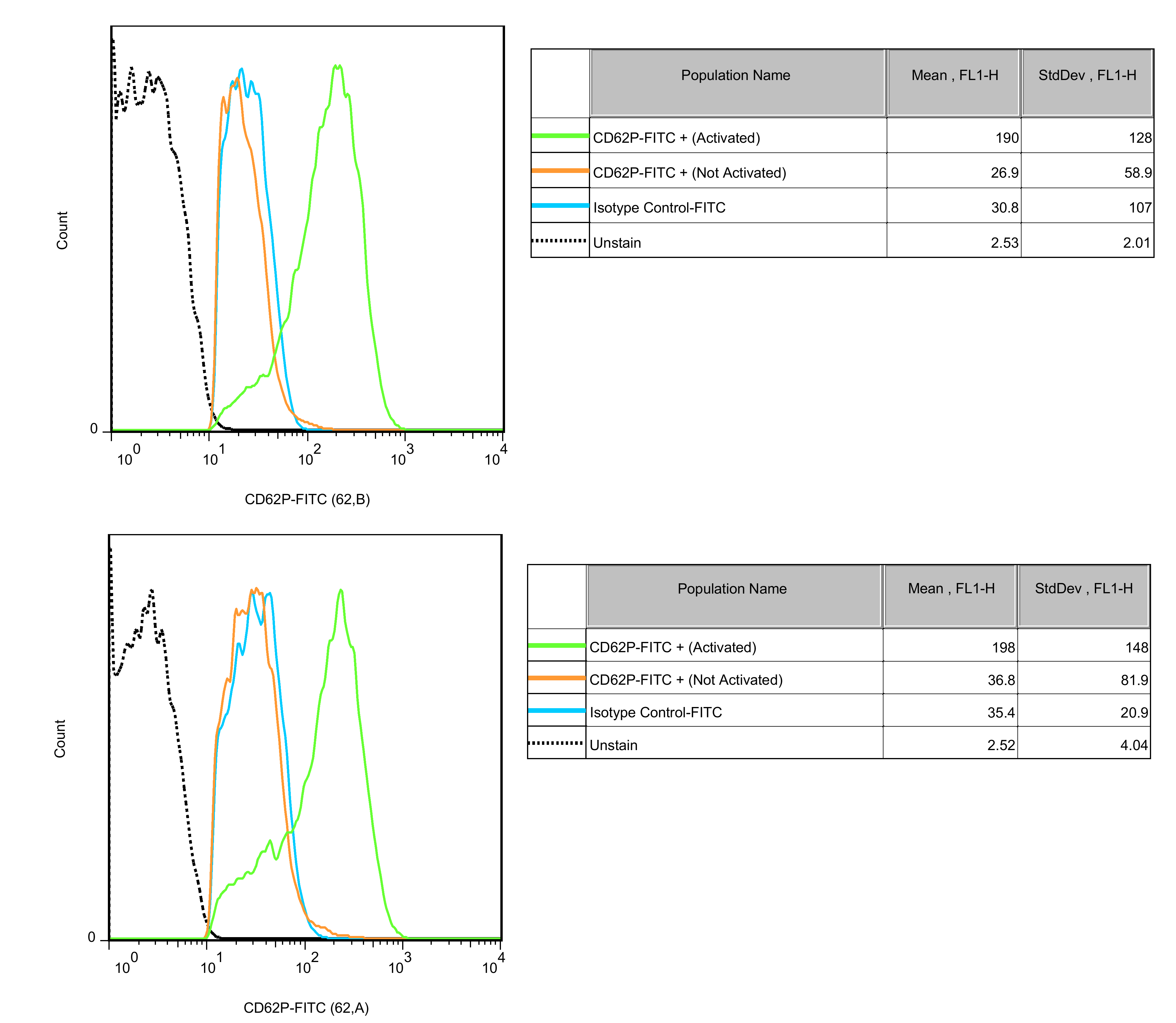

Supplement: Supplementary file 2 — Additional file 2: The Flow Cytometric assay results of per-patient levels of platelet CD62P Ag expression at baseline (B) and week 26 (A). [file 13098_2022_951_MOESM2_ESM.zip › 62.png]

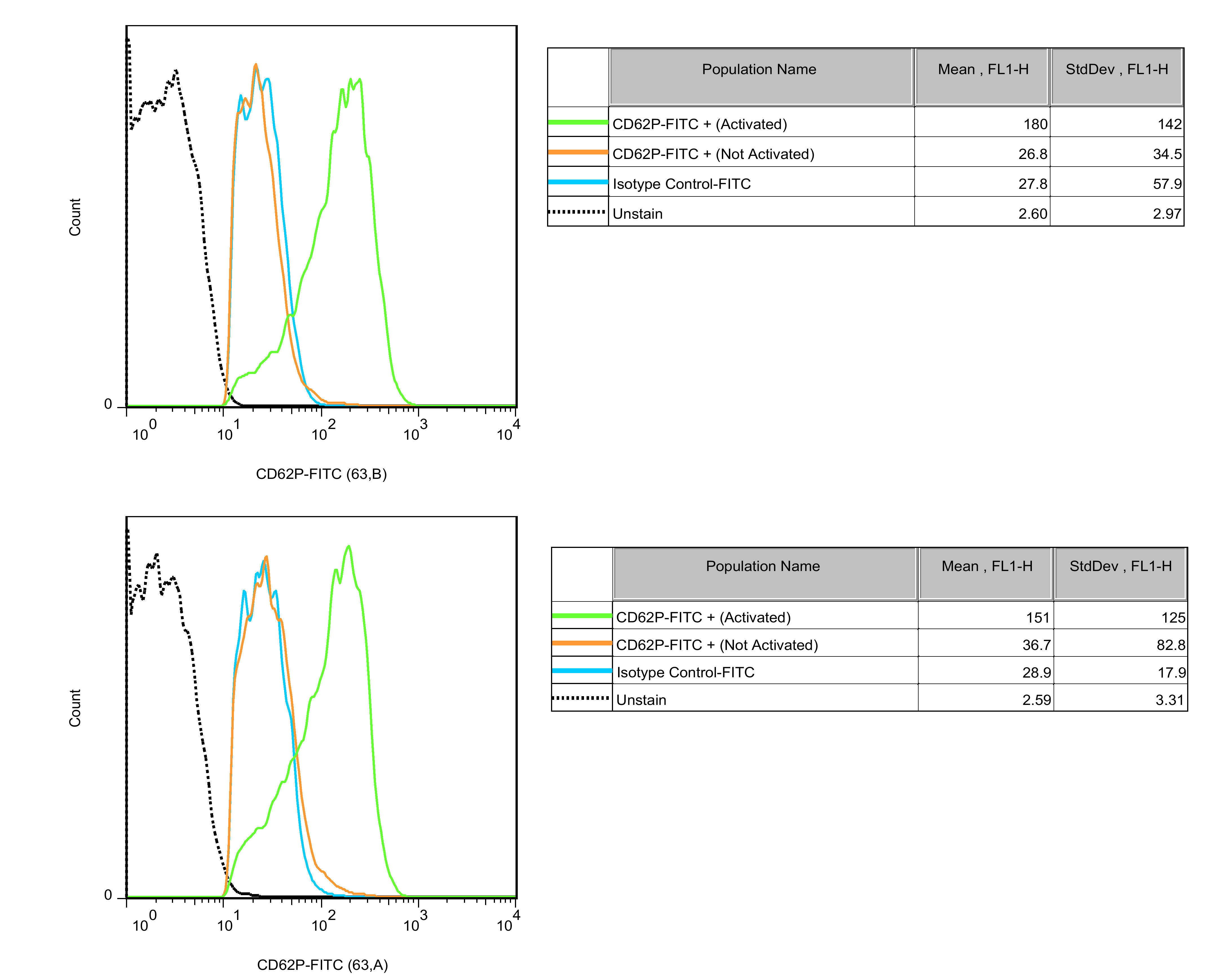

Supplement: Supplementary file 2 — Additional file 2: The Flow Cytometric assay results of per-patient levels of platelet CD62P Ag expression at baseline (B) and week 26 (A). [file 13098_2022_951_MOESM2_ESM.zip › 63.png]

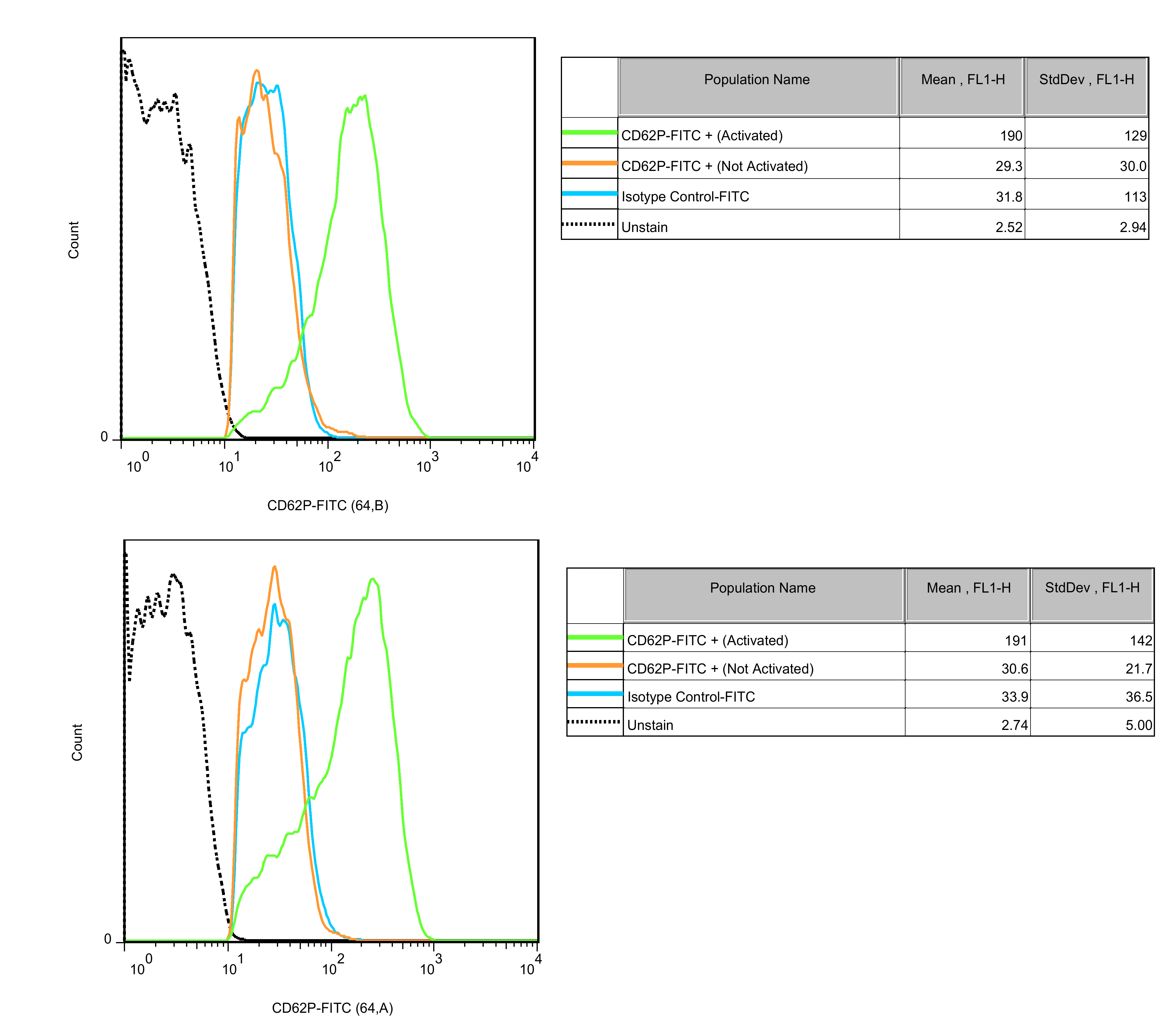

Supplement: Supplementary file 2 — Additional file 2: The Flow Cytometric assay results of per-patient levels of platelet CD62P Ag expression at baseline (B) and week 26 (A). [file 13098_2022_951_MOESM2_ESM.zip › 64.png]

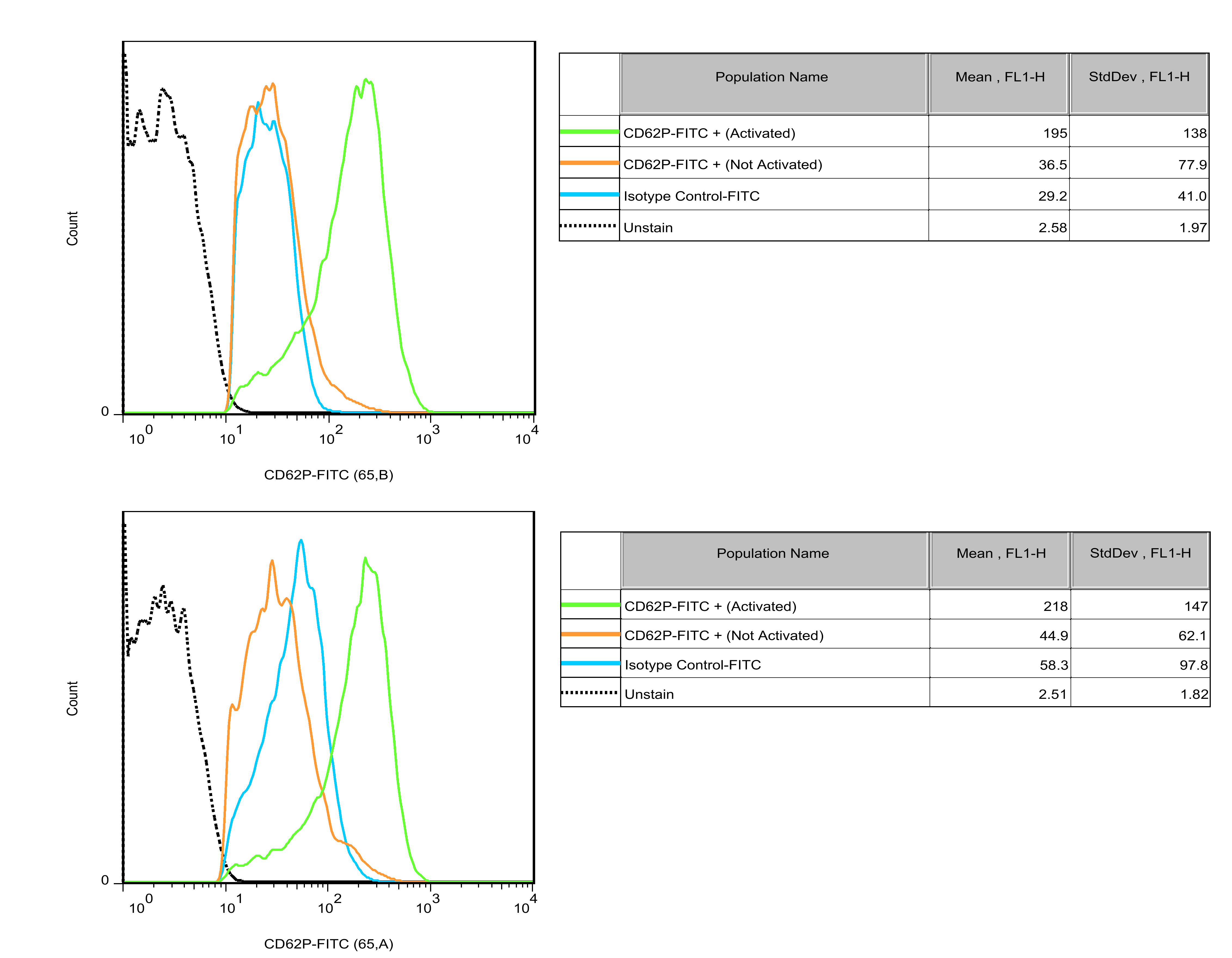

Supplement: Supplementary file 2 — Additional file 2: The Flow Cytometric assay results of per-patient levels of platelet CD62P Ag expression at baseline (B) and week 26 (A). [file 13098_2022_951_MOESM2_ESM.zip › 65.png]

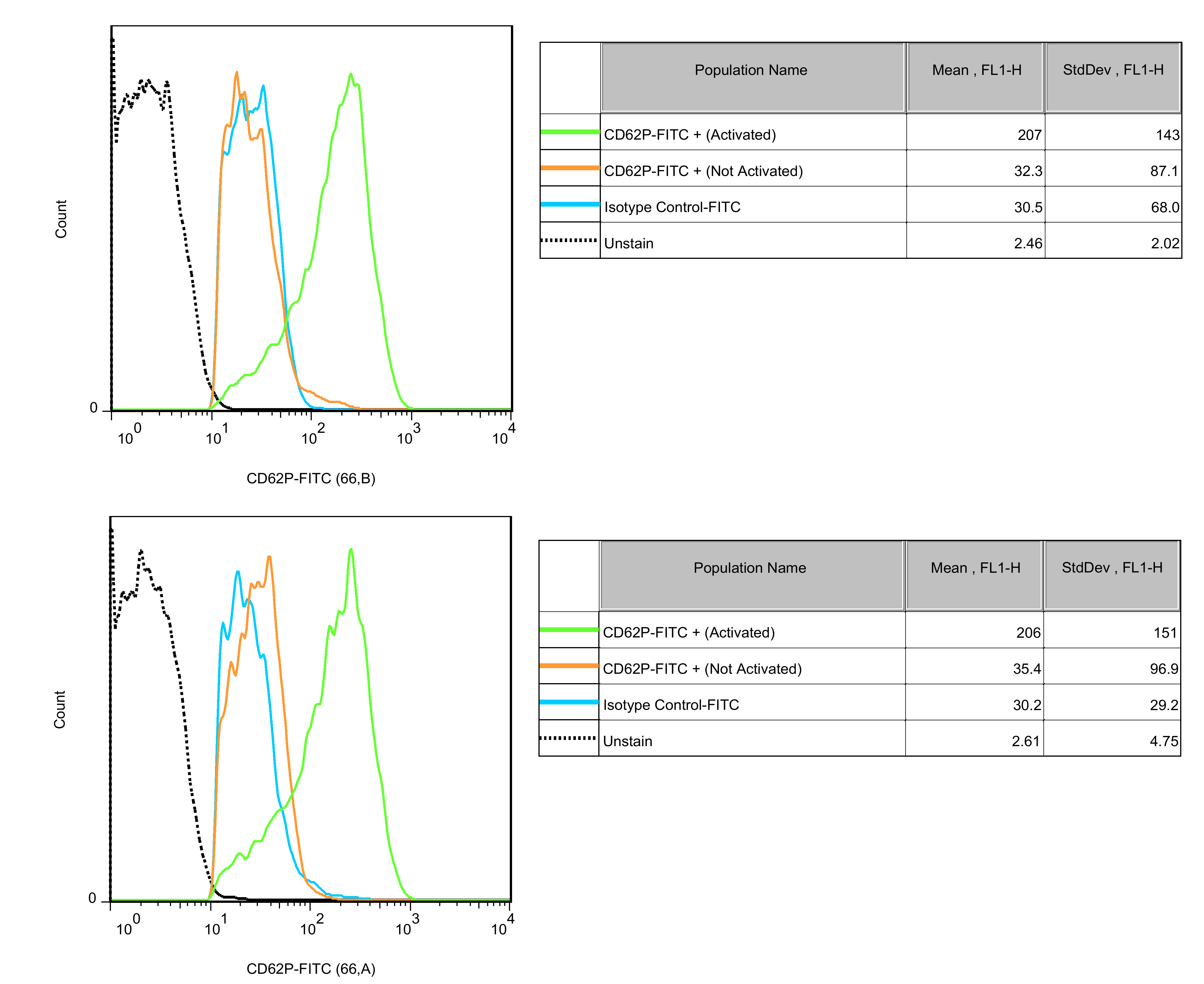

Supplement: Supplementary file 2 — Additional file 2: The Flow Cytometric assay results of per-patient levels of platelet CD62P Ag expression at baseline (B) and week 26 (A). [file 13098_2022_951_MOESM2_ESM.zip › 66.png]

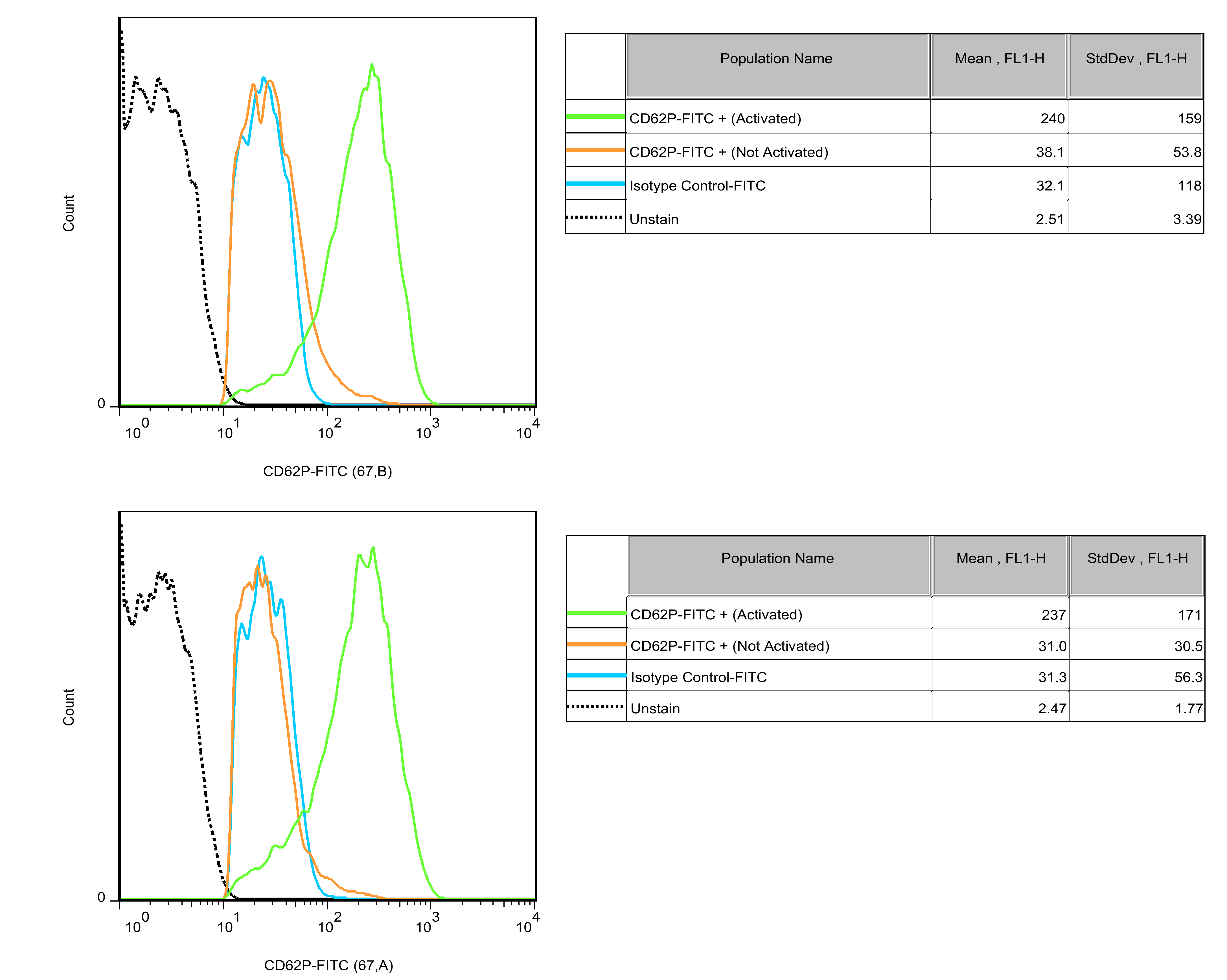

Supplement: Supplementary file 2 — Additional file 2: The Flow Cytometric assay results of per-patient levels of platelet CD62P Ag expression at baseline (B) and week 26 (A). [file 13098_2022_951_MOESM2_ESM.zip › 67.png]

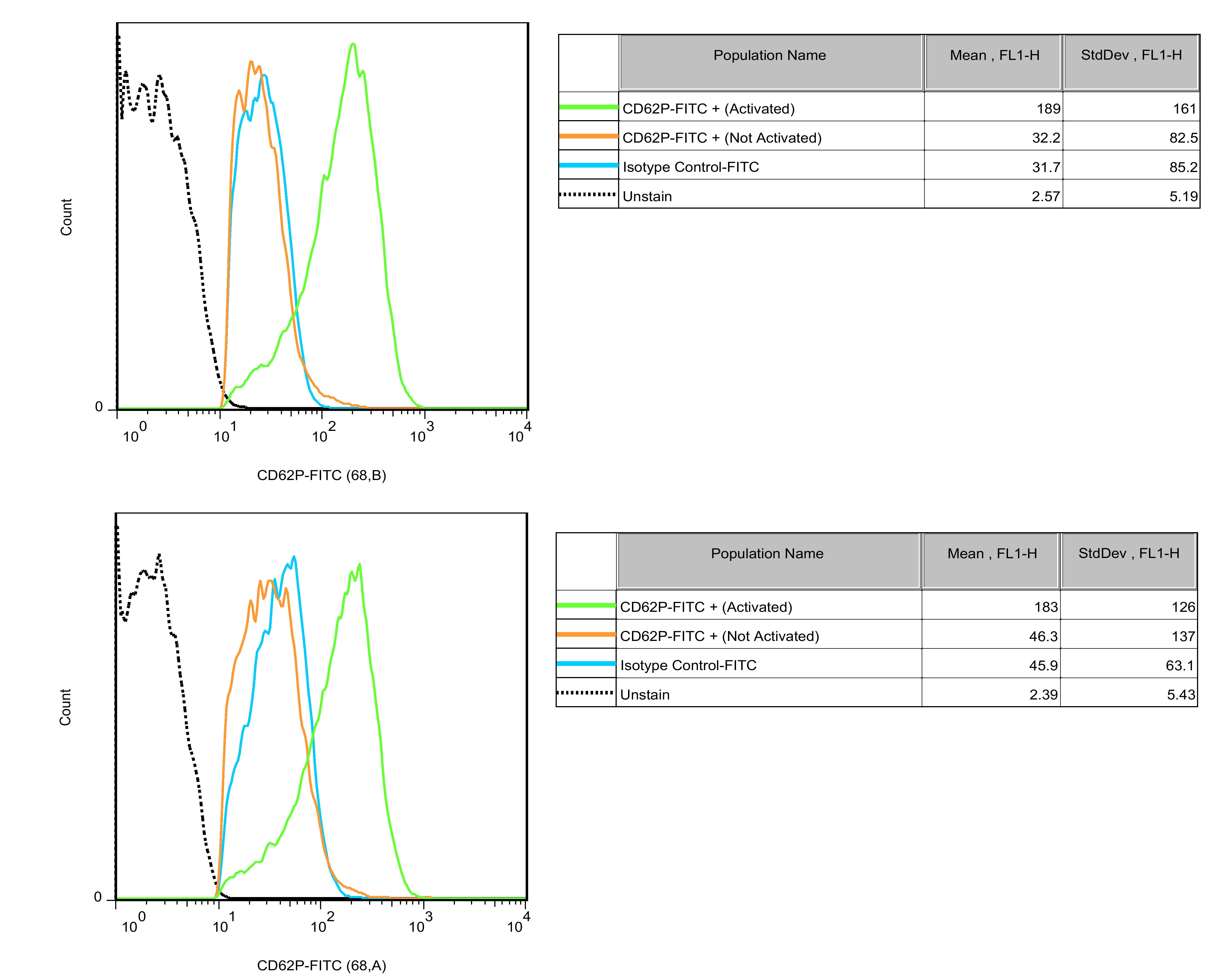

Supplement: Supplementary file 2 — Additional file 2: The Flow Cytometric assay results of per-patient levels of platelet CD62P Ag expression at baseline (B) and week 26 (A). [file 13098_2022_951_MOESM2_ESM.zip › 68.png]

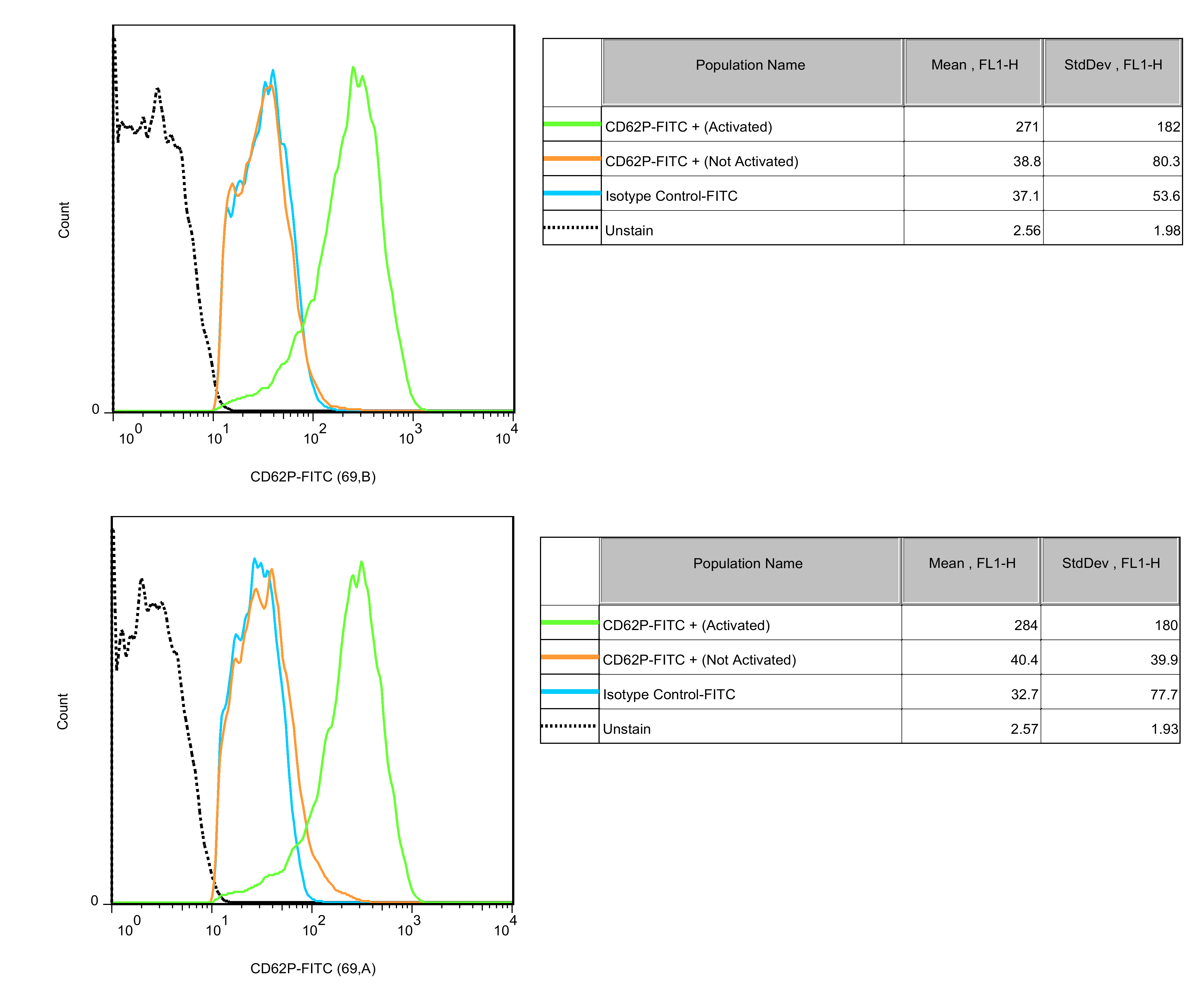

Supplement: Supplementary file 2 — Additional file 2: The Flow Cytometric assay results of per-patient levels of platelet CD62P Ag expression at baseline (B) and week 26 (A). [file 13098_2022_951_MOESM2_ESM.zip › 69.png]

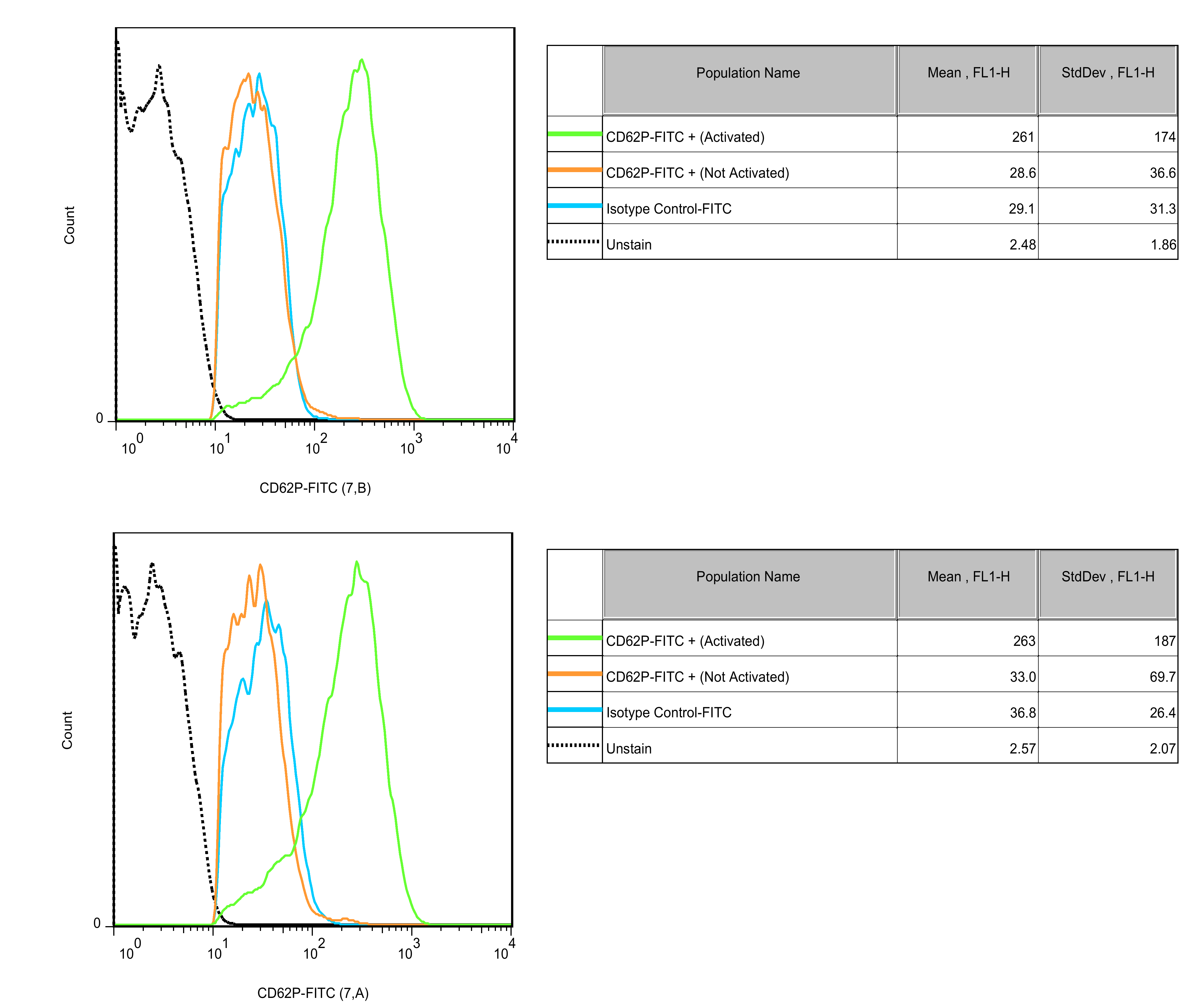

Supplement: Supplementary file 2 — Additional file 2: The Flow Cytometric assay results of per-patient levels of platelet CD62P Ag expression at baseline (B) and week 26 (A). [file 13098_2022_951_MOESM2_ESM.zip › 7.png]

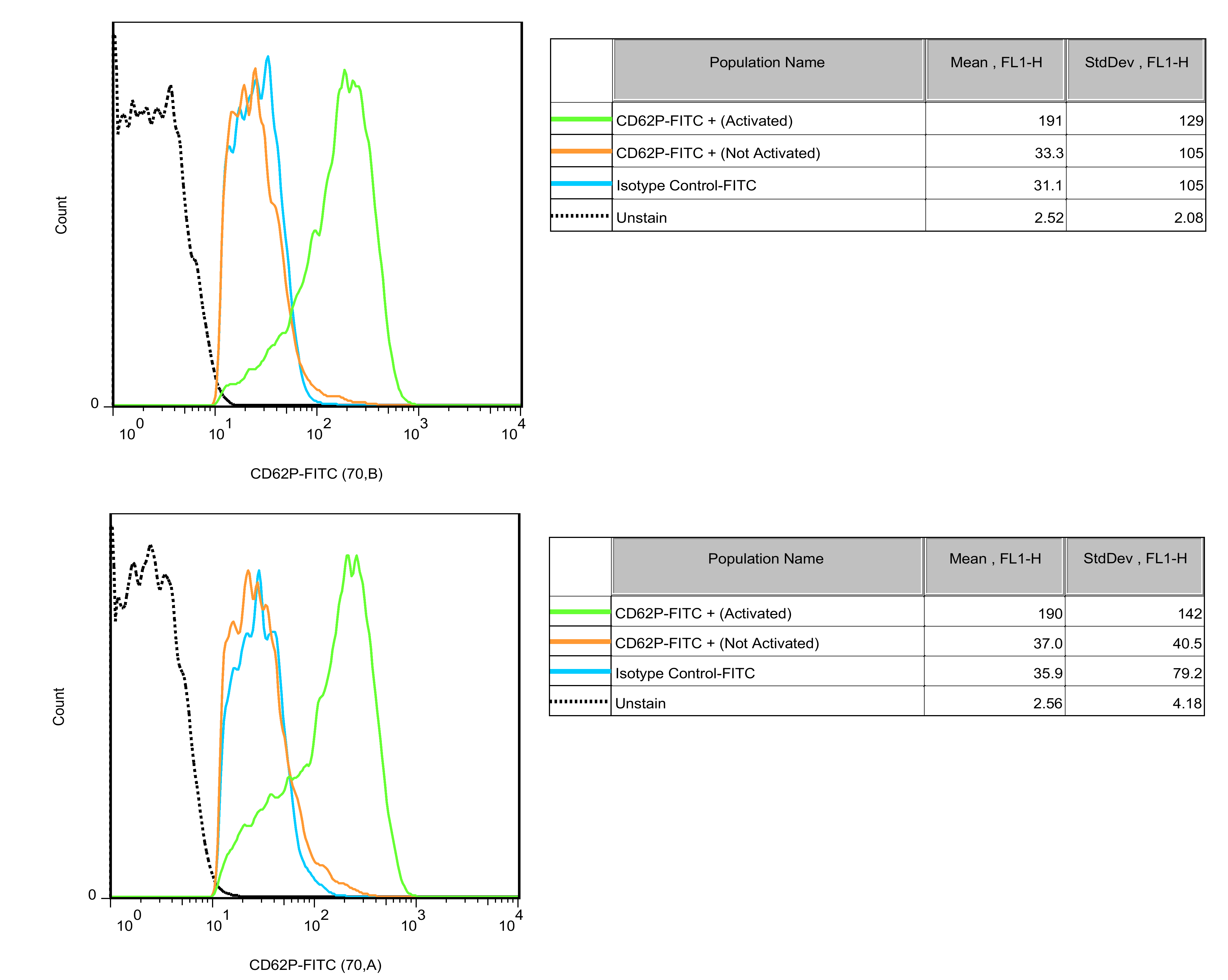

Supplement: Supplementary file 2 — Additional file 2: The Flow Cytometric assay results of per-patient levels of platelet CD62P Ag expression at baseline (B) and week 26 (A). [file 13098_2022_951_MOESM2_ESM.zip › 70.png]

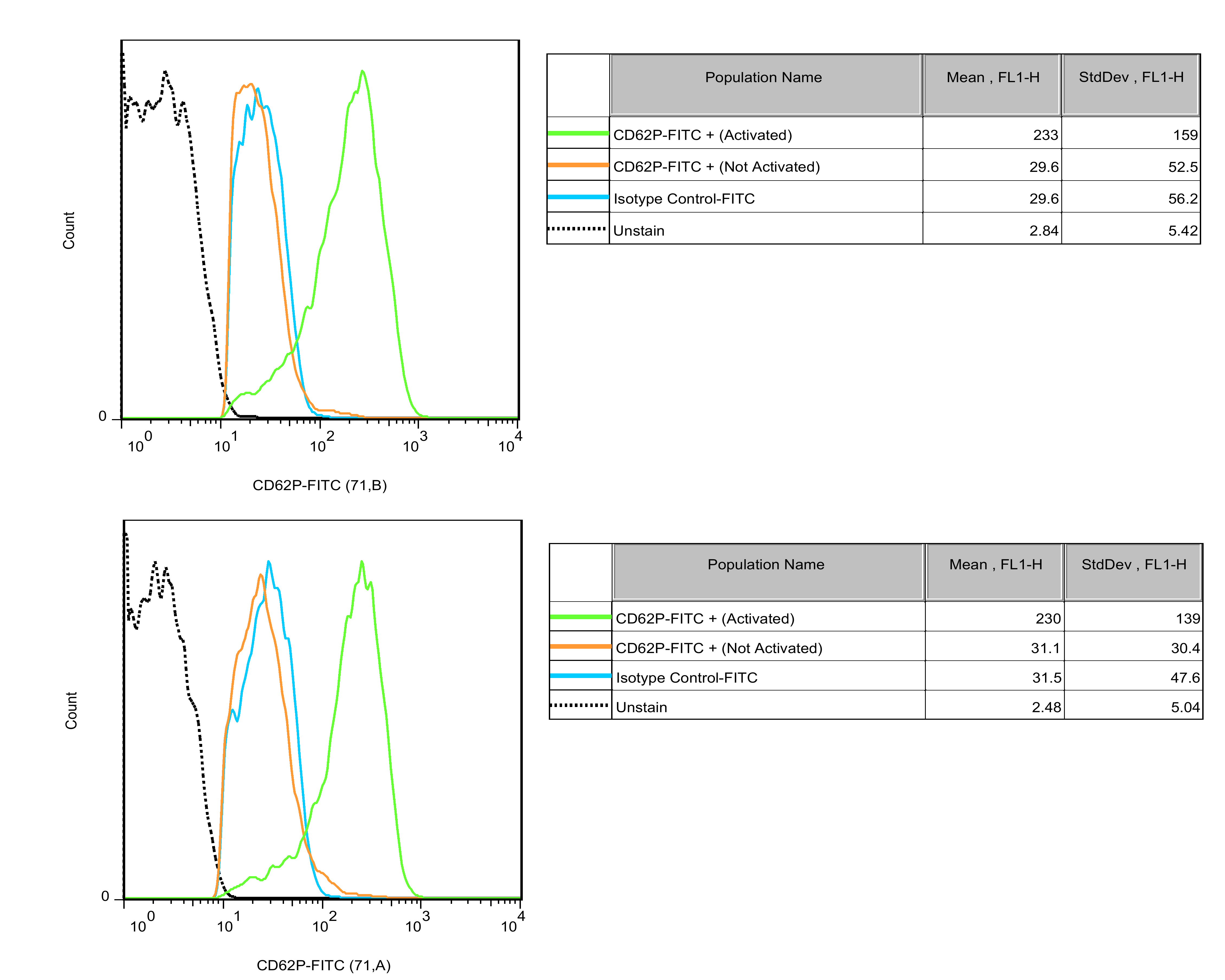

Supplement: Supplementary file 2 — Additional file 2: The Flow Cytometric assay results of per-patient levels of platelet CD62P Ag expression at baseline (B) and week 26 (A). [file 13098_2022_951_MOESM2_ESM.zip › 71.png]

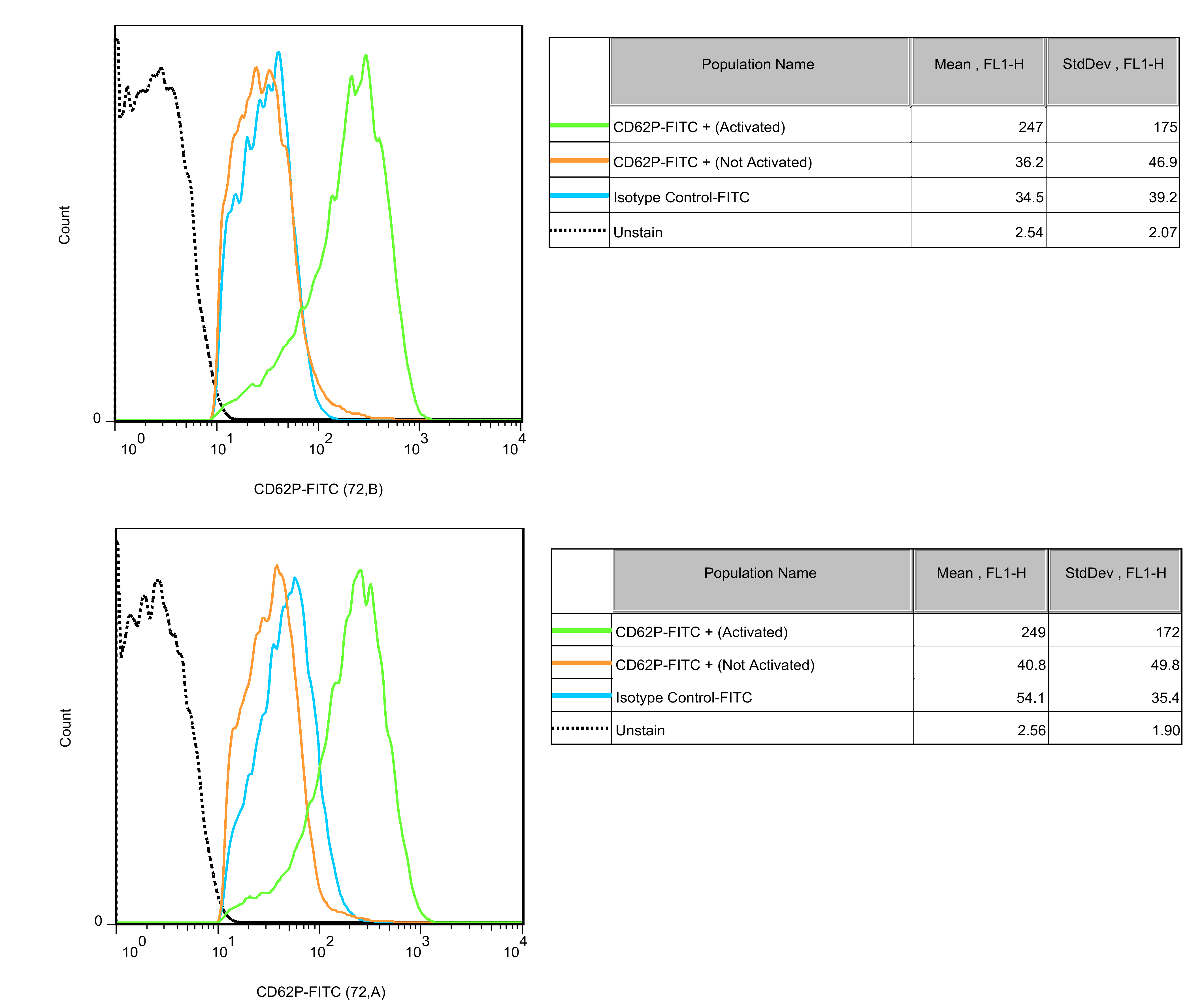

Supplement: Supplementary file 2 — Additional file 2: The Flow Cytometric assay results of per-patient levels of platelet CD62P Ag expression at baseline (B) and week 26 (A). [file 13098_2022_951_MOESM2_ESM.zip › 72.png]

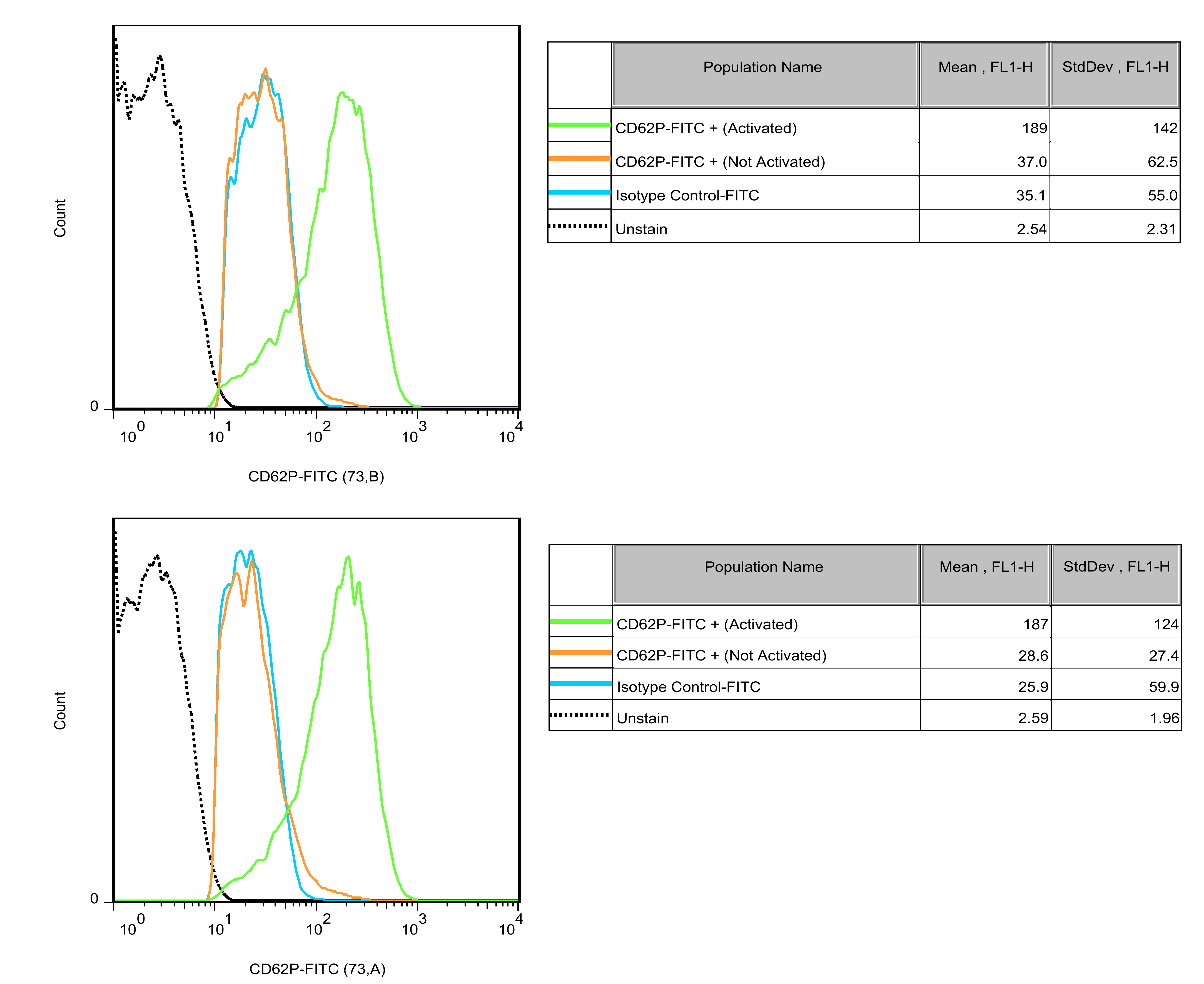

Supplement: Supplementary file 2 — Additional file 2: The Flow Cytometric assay results of per-patient levels of platelet CD62P Ag expression at baseline (B) and week 26 (A). [file 13098_2022_951_MOESM2_ESM.zip › 73.png]

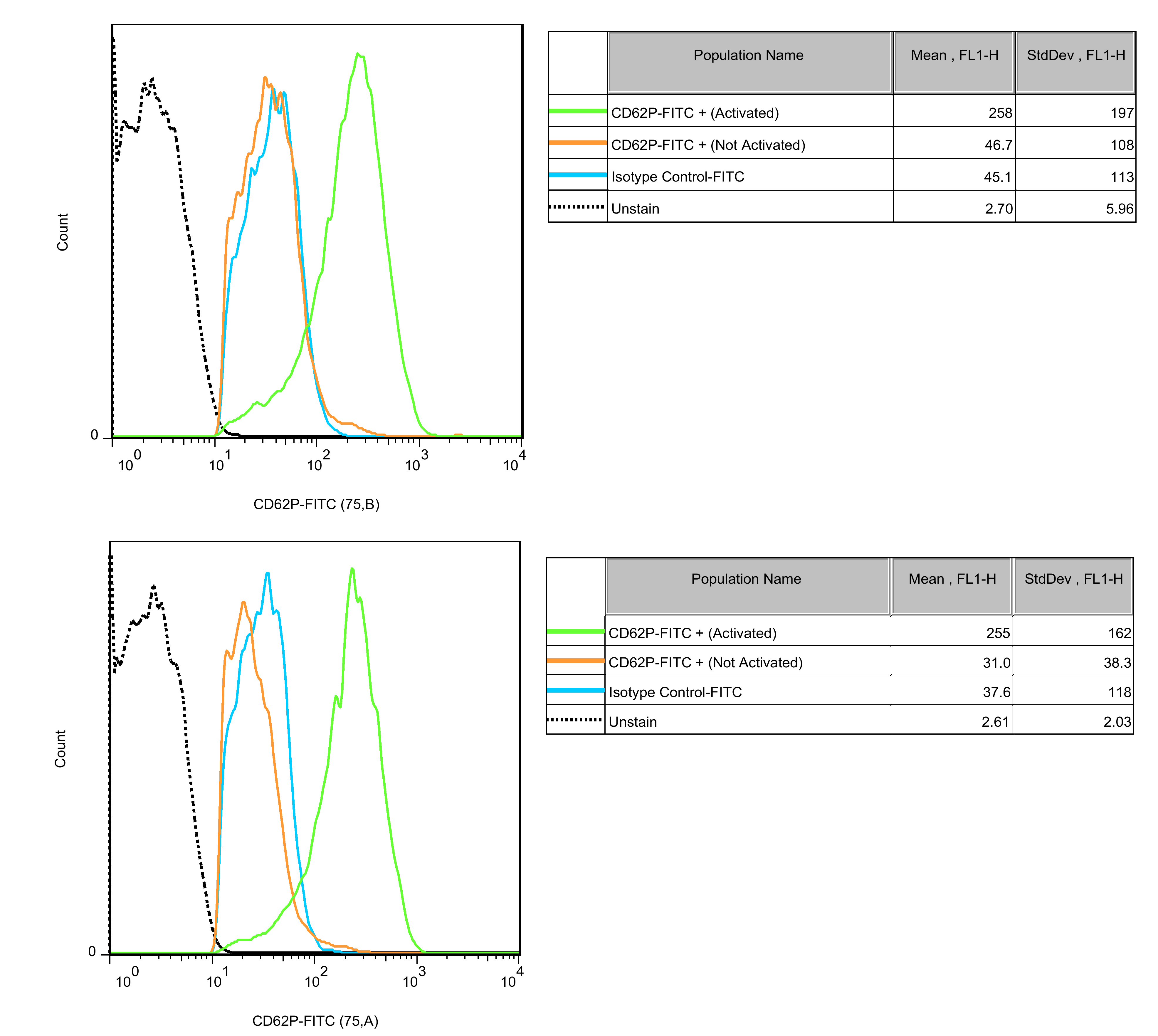

Supplement: Supplementary file 2 — Additional file 2: The Flow Cytometric assay results of per-patient levels of platelet CD62P Ag expression at baseline (B) and week 26 (A). [file 13098_2022_951_MOESM2_ESM.zip › 75.png]

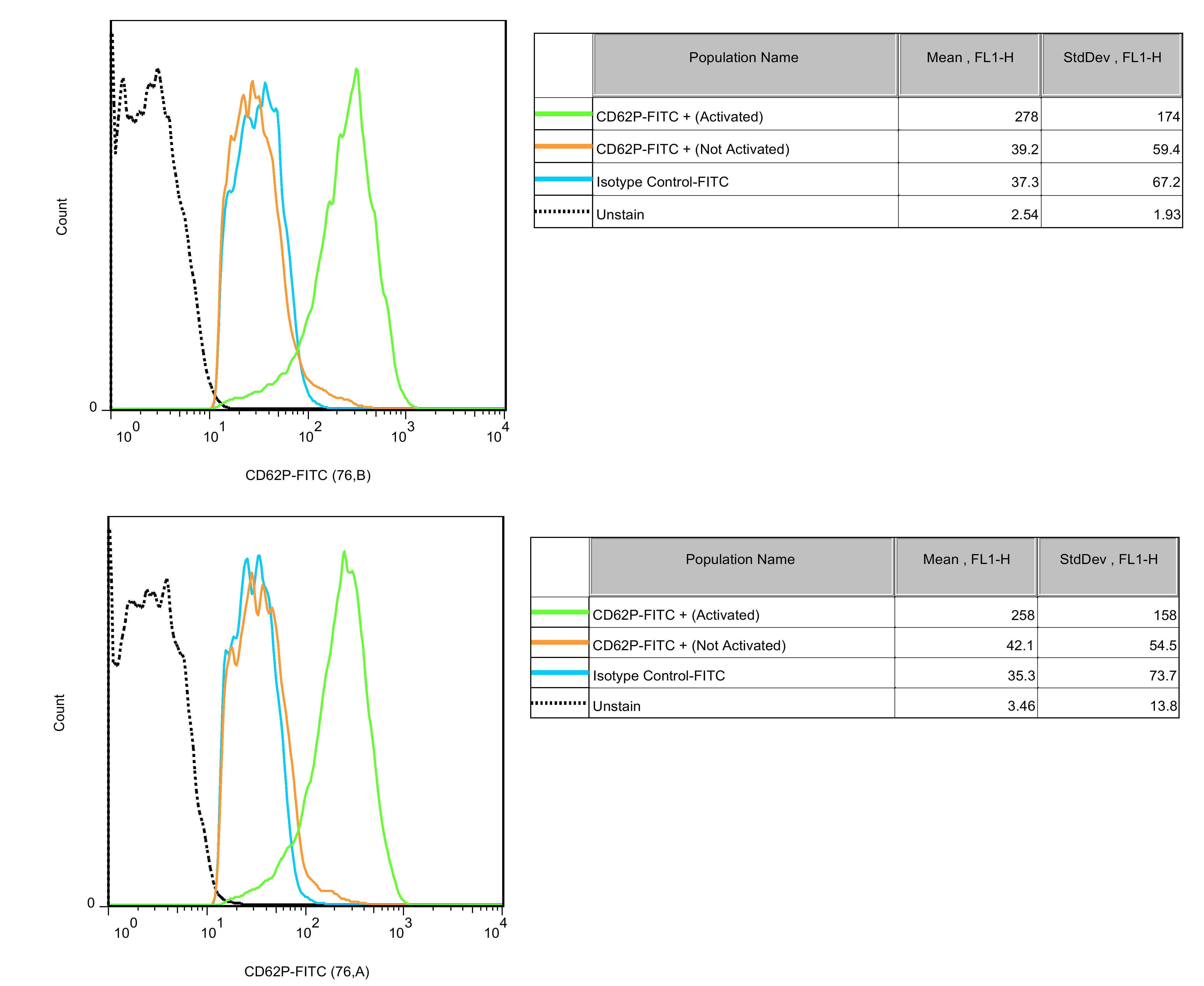

Supplement: Supplementary file 2 — Additional file 2: The Flow Cytometric assay results of per-patient levels of platelet CD62P Ag expression at baseline (B) and week 26 (A). [file 13098_2022_951_MOESM2_ESM.zip › 76.png]

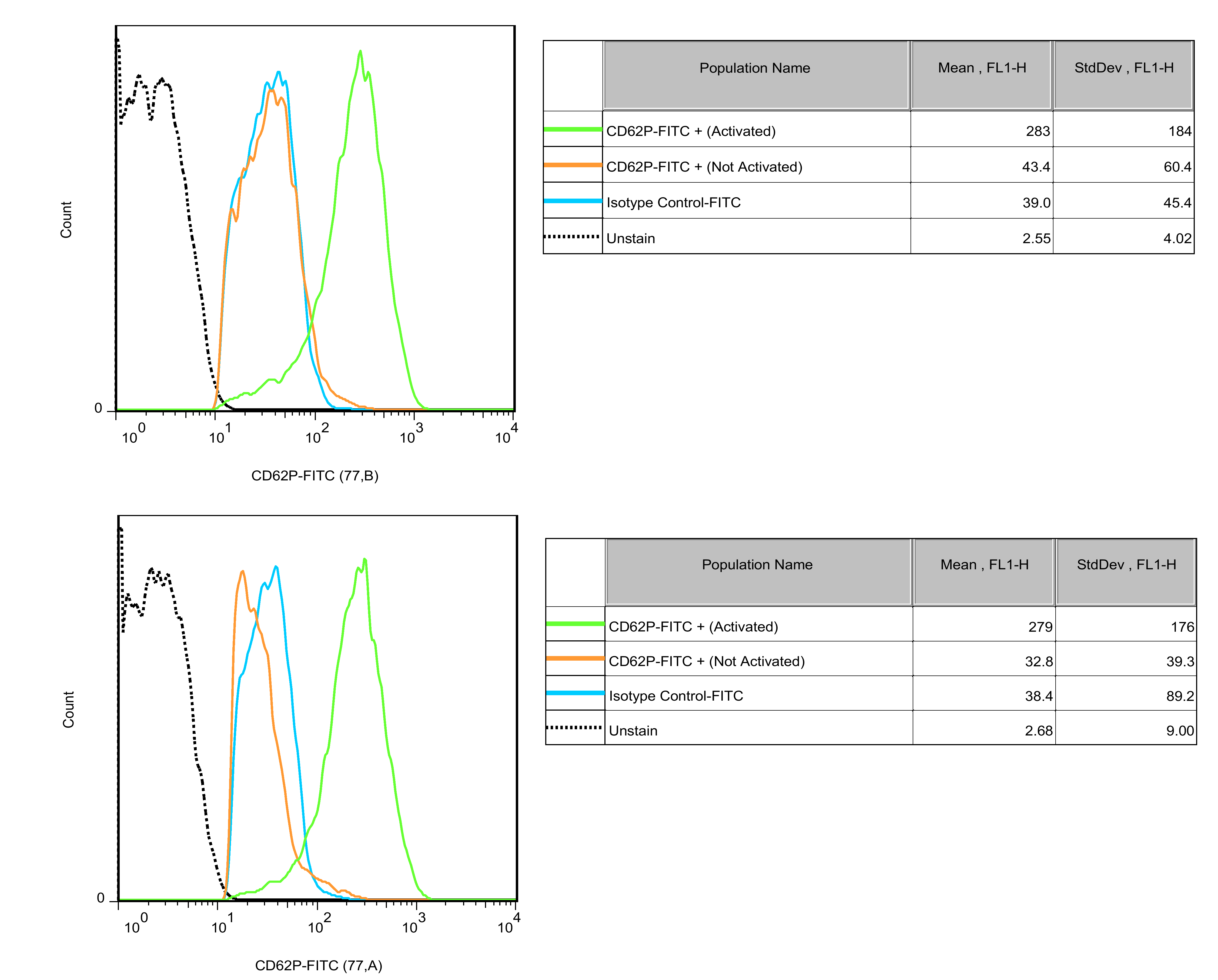

Supplement: Supplementary file 2 — Additional file 2: The Flow Cytometric assay results of per-patient levels of platelet CD62P Ag expression at baseline (B) and week 26 (A). [file 13098_2022_951_MOESM2_ESM.zip › 77.png]

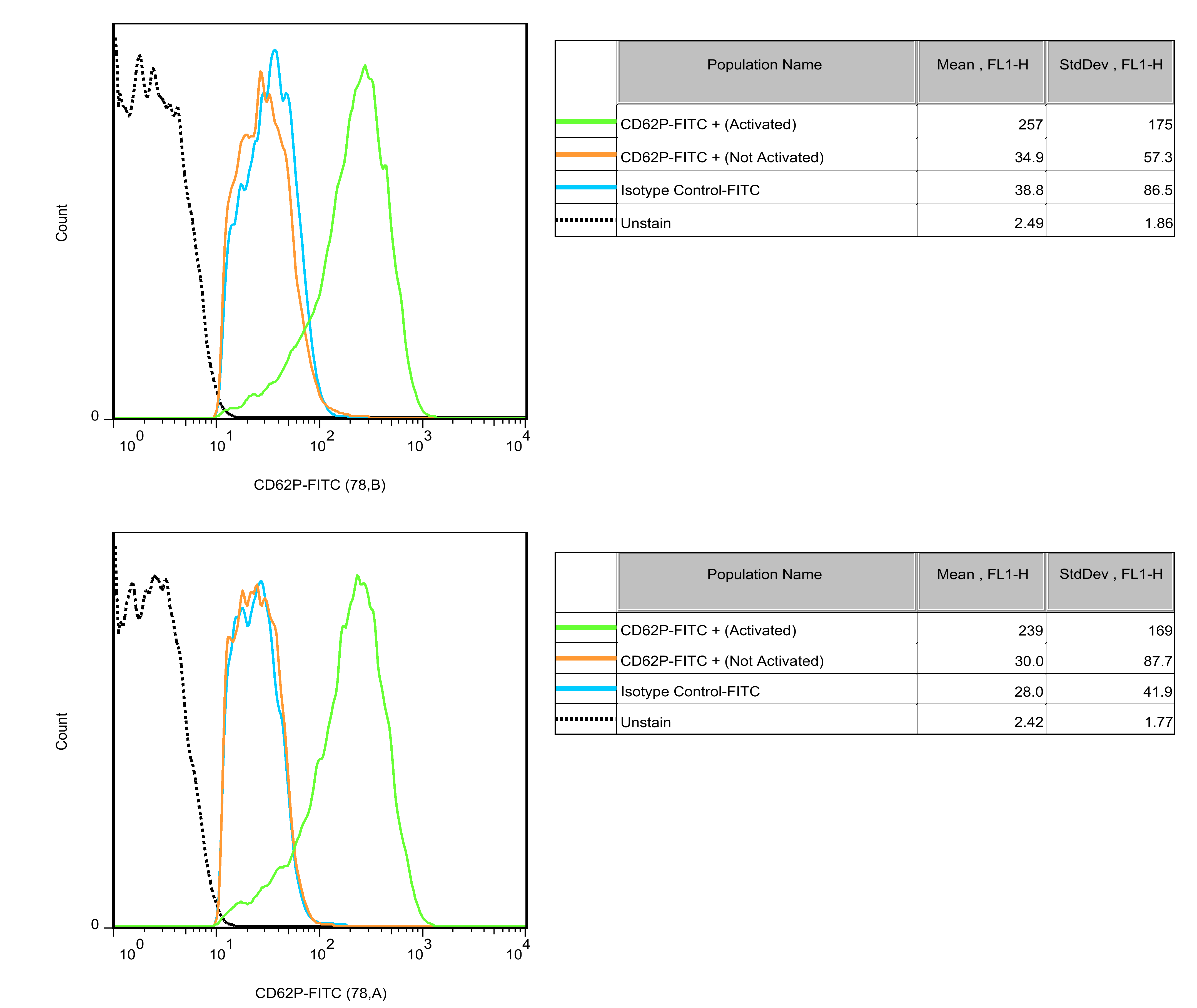

Supplement: Supplementary file 2 — Additional file 2: The Flow Cytometric assay results of per-patient levels of platelet CD62P Ag expression at baseline (B) and week 26 (A). [file 13098_2022_951_MOESM2_ESM.zip › 78.png]

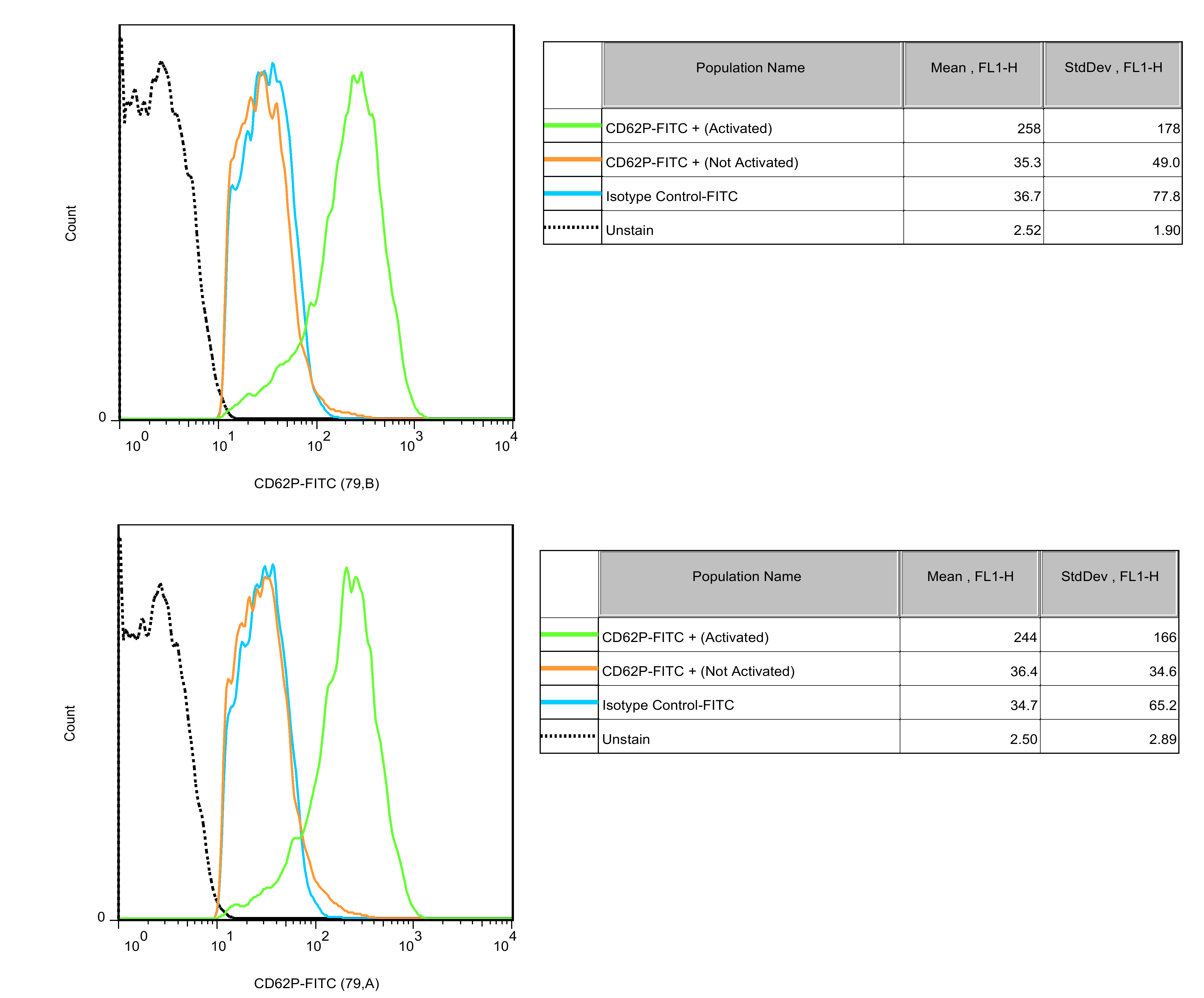

Supplement: Supplementary file 2 — Additional file 2: The Flow Cytometric assay results of per-patient levels of platelet CD62P Ag expression at baseline (B) and week 26 (A). [file 13098_2022_951_MOESM2_ESM.zip › 79.png]

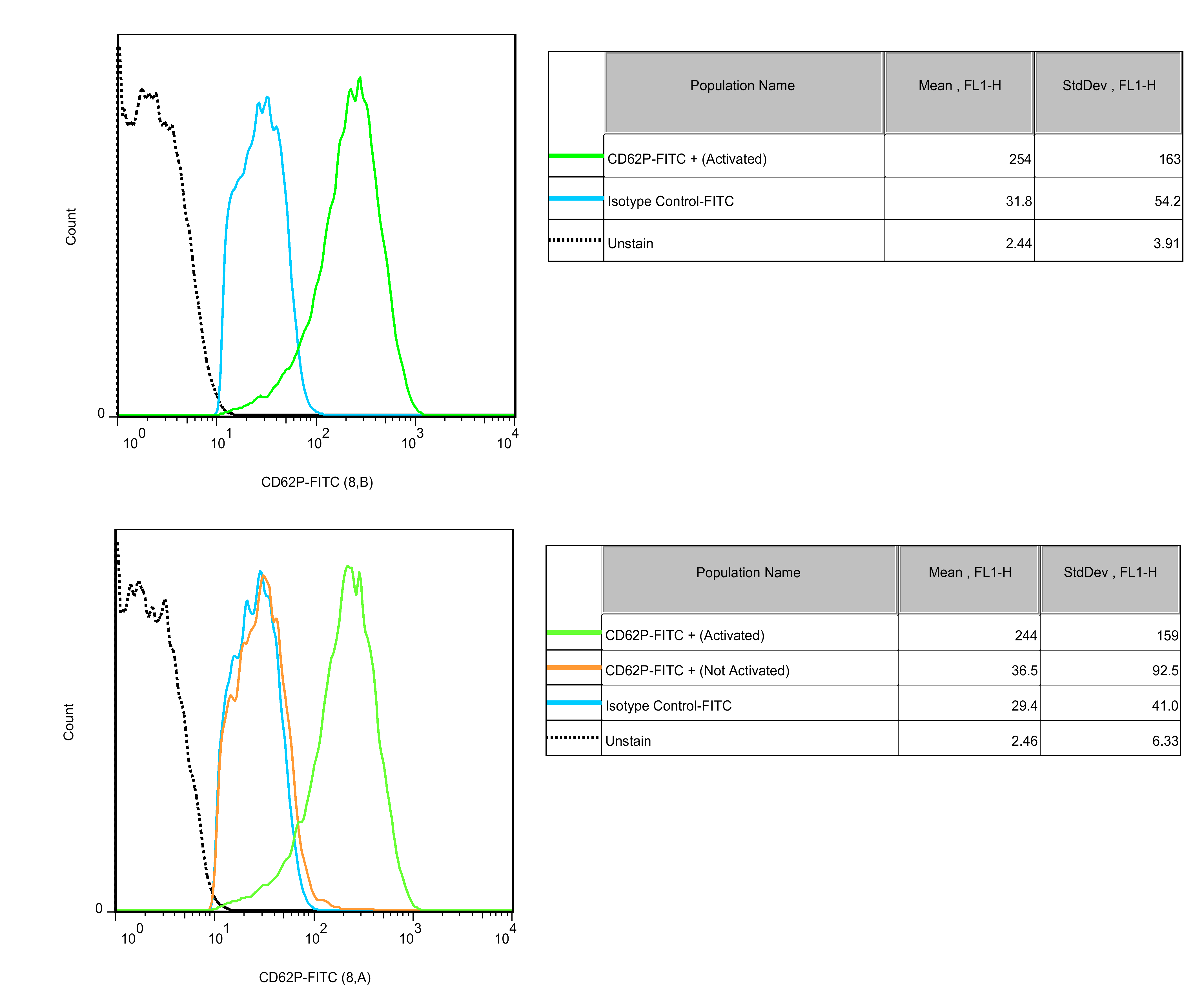

Supplement: Supplementary file 2 — Additional file 2: The Flow Cytometric assay results of per-patient levels of platelet CD62P Ag expression at baseline (B) and week 26 (A). [file 13098_2022_951_MOESM2_ESM.zip › 8.png]

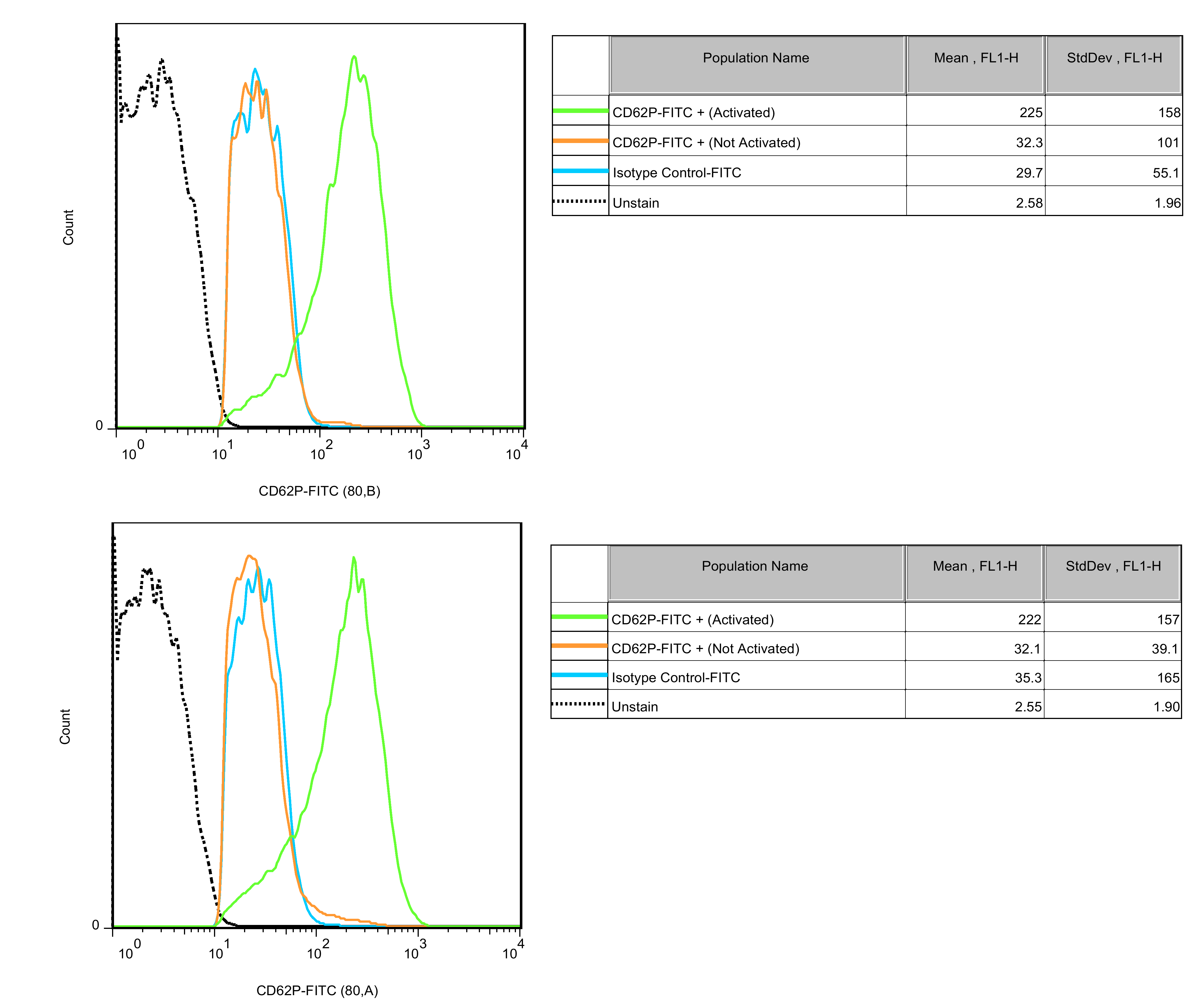

Supplement: Supplementary file 2 — Additional file 2: The Flow Cytometric assay results of per-patient levels of platelet CD62P Ag expression at baseline (B) and week 26 (A). [file 13098_2022_951_MOESM2_ESM.zip › 80.png]

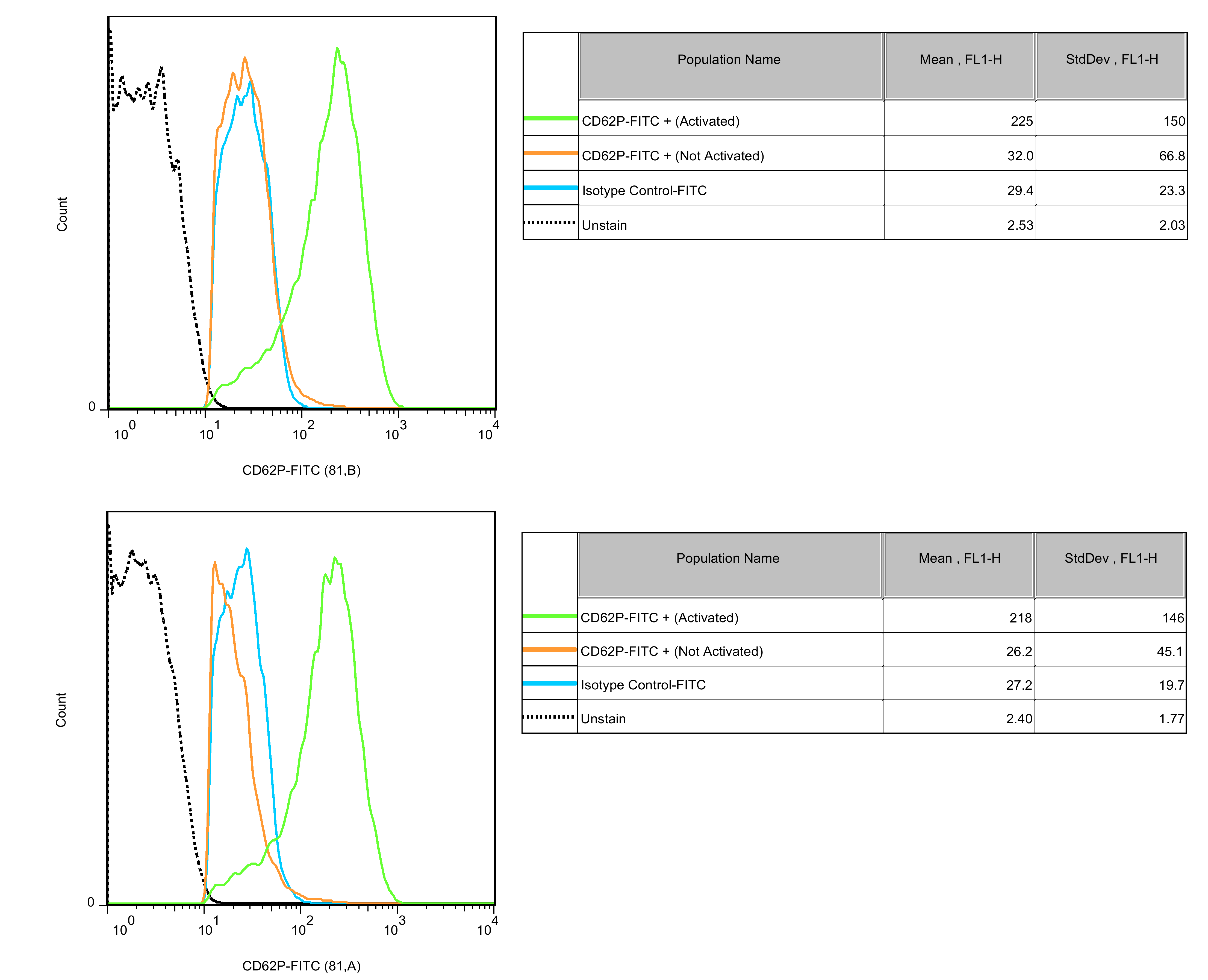

Supplement: Supplementary file 2 — Additional file 2: The Flow Cytometric assay results of per-patient levels of platelet CD62P Ag expression at baseline (B) and week 26 (A). [file 13098_2022_951_MOESM2_ESM.zip › 81.png]

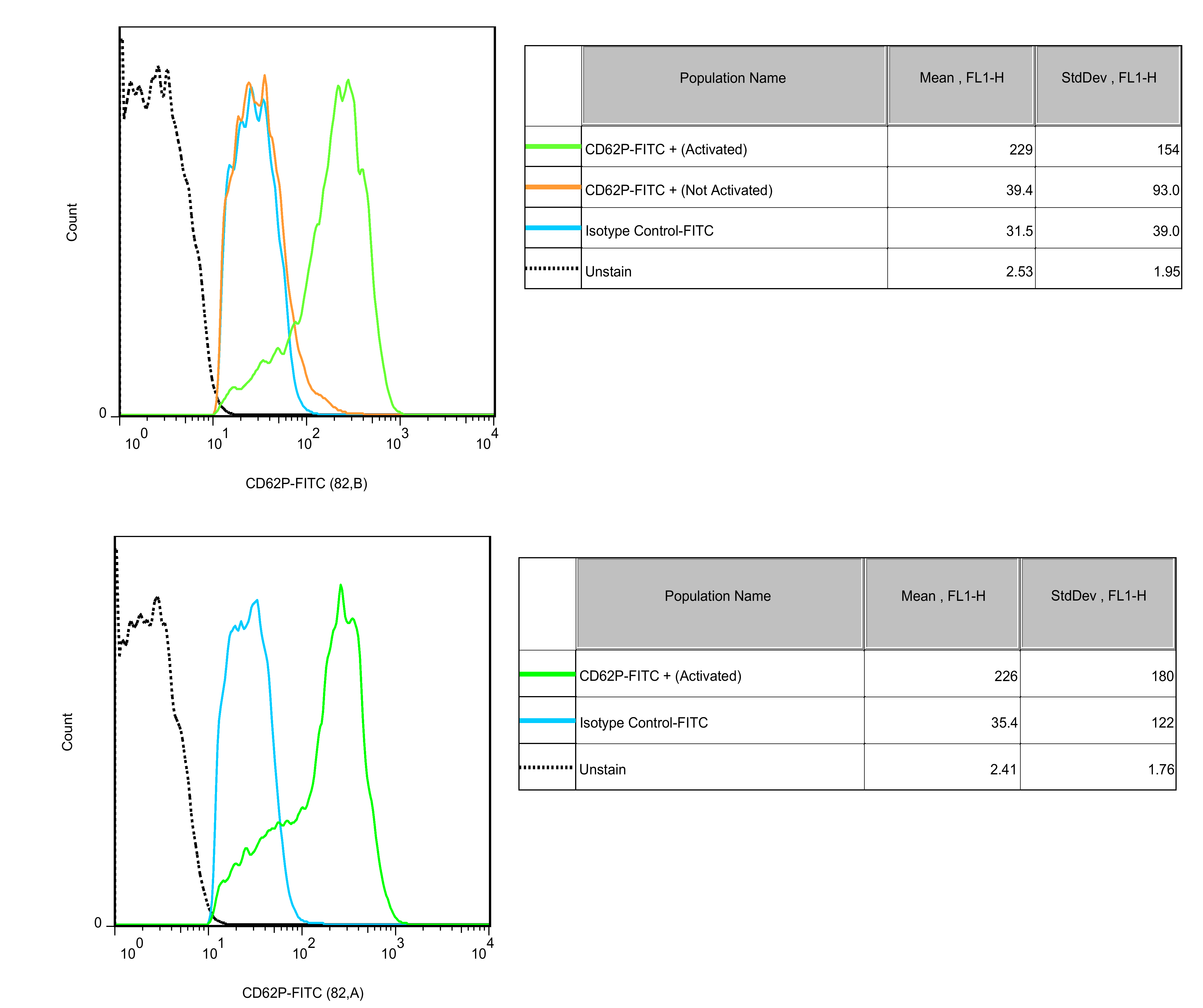

Supplement: Supplementary file 2 — Additional file 2: The Flow Cytometric assay results of per-patient levels of platelet CD62P Ag expression at baseline (B) and week 26 (A). [file 13098_2022_951_MOESM2_ESM.zip › 82.png]

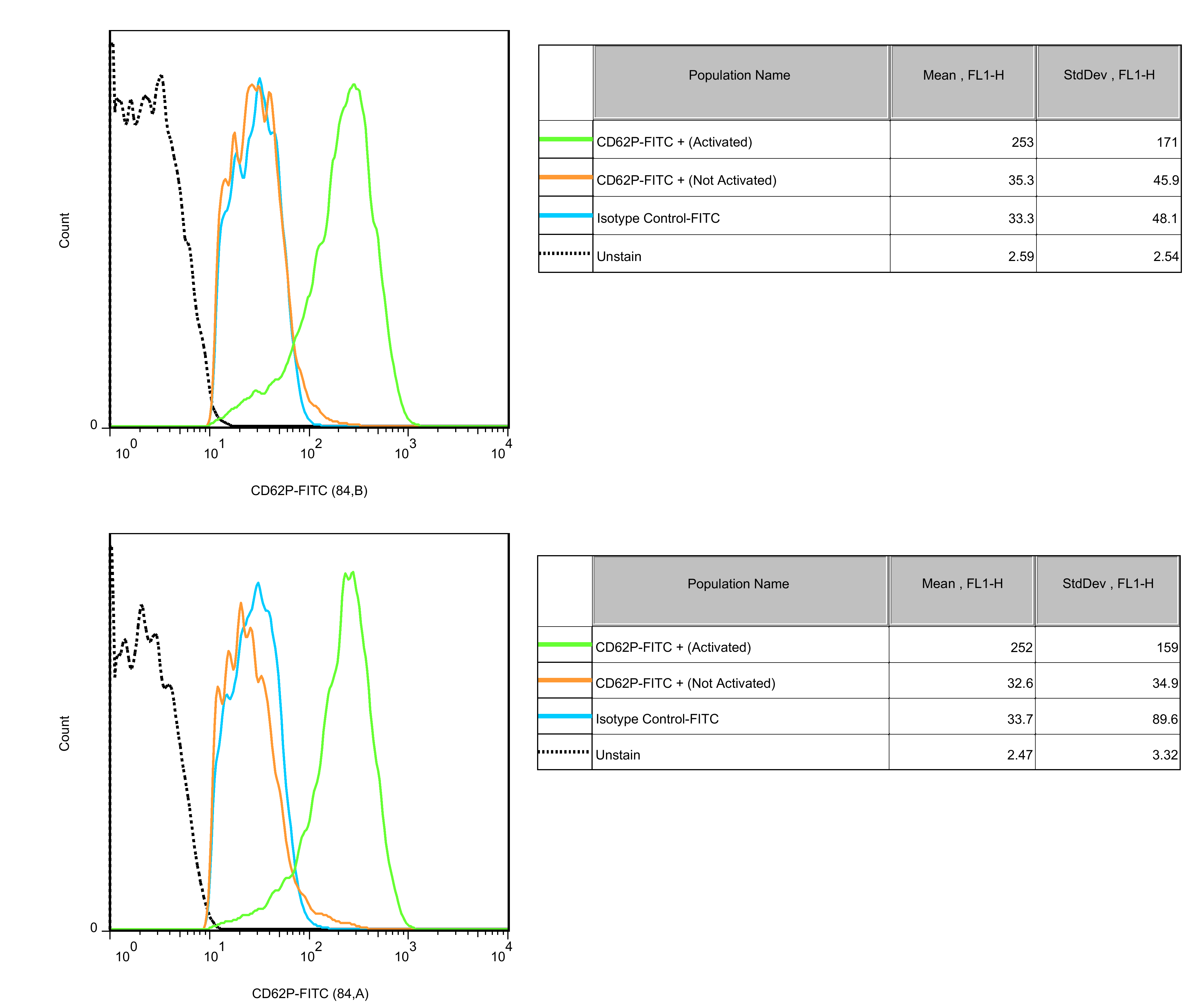

Supplement: Supplementary file 2 — Additional file 2: The Flow Cytometric assay results of per-patient levels of platelet CD62P Ag expression at baseline (B) and week 26 (A). [file 13098_2022_951_MOESM2_ESM.zip › 84.png]

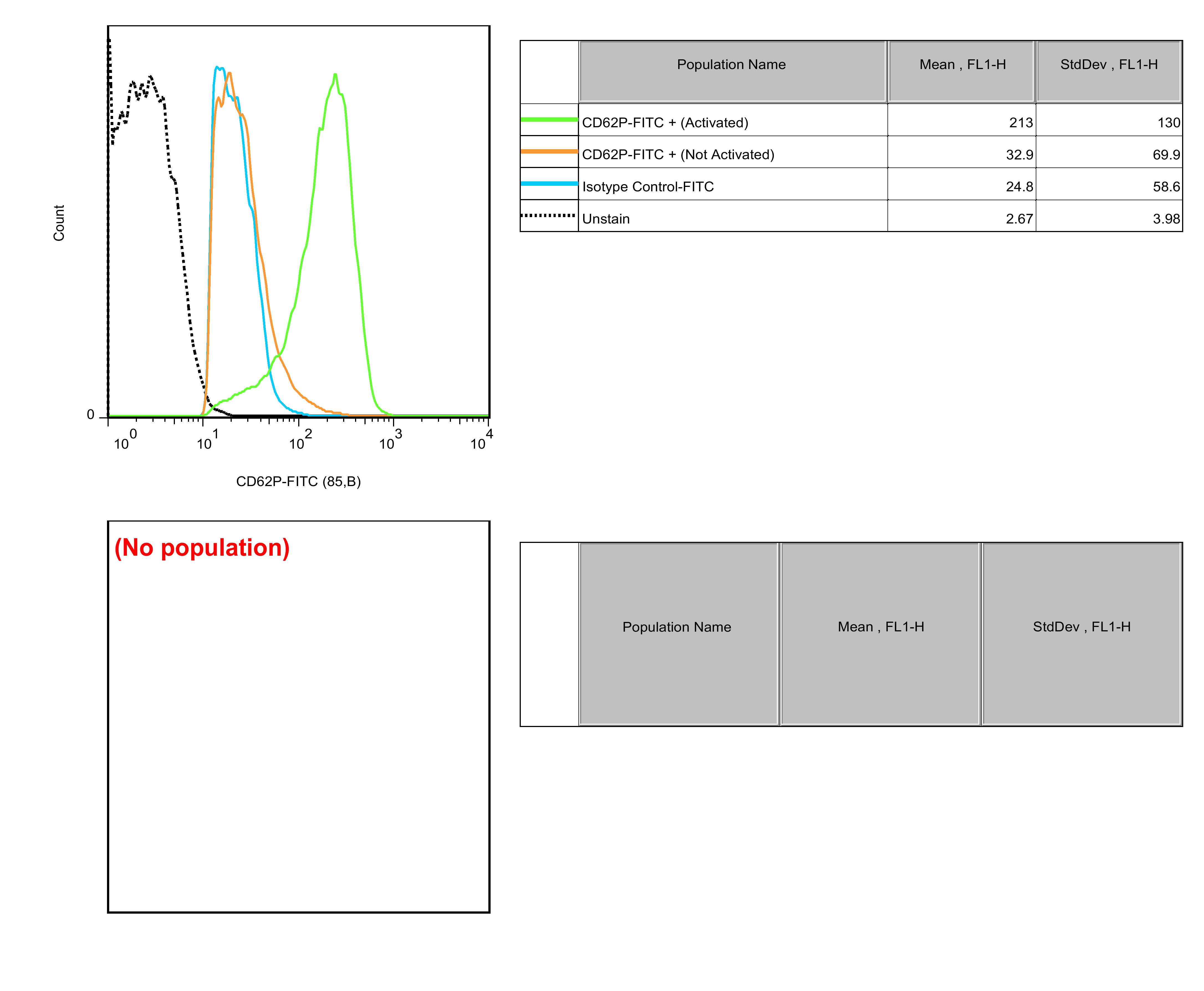

Supplement: Supplementary file 2 — Additional file 2: The Flow Cytometric assay results of per-patient levels of platelet CD62P Ag expression at baseline (B) and week 26 (A). [file 13098_2022_951_MOESM2_ESM.zip › 85.png]

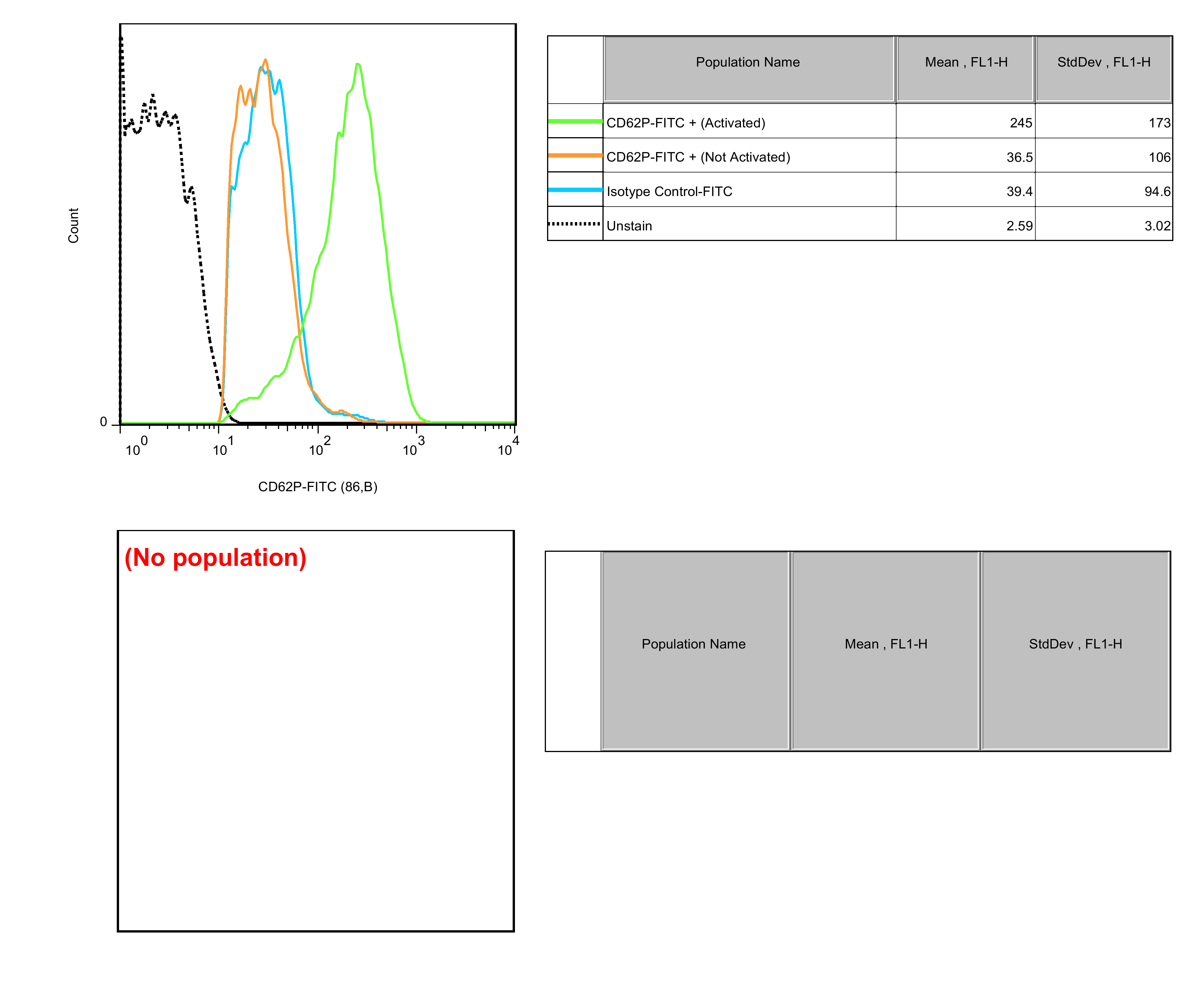

Supplement: Supplementary file 2 — Additional file 2: The Flow Cytometric assay results of per-patient levels of platelet CD62P Ag expression at baseline (B) and week 26 (A). [file 13098_2022_951_MOESM2_ESM.zip › 86.png]

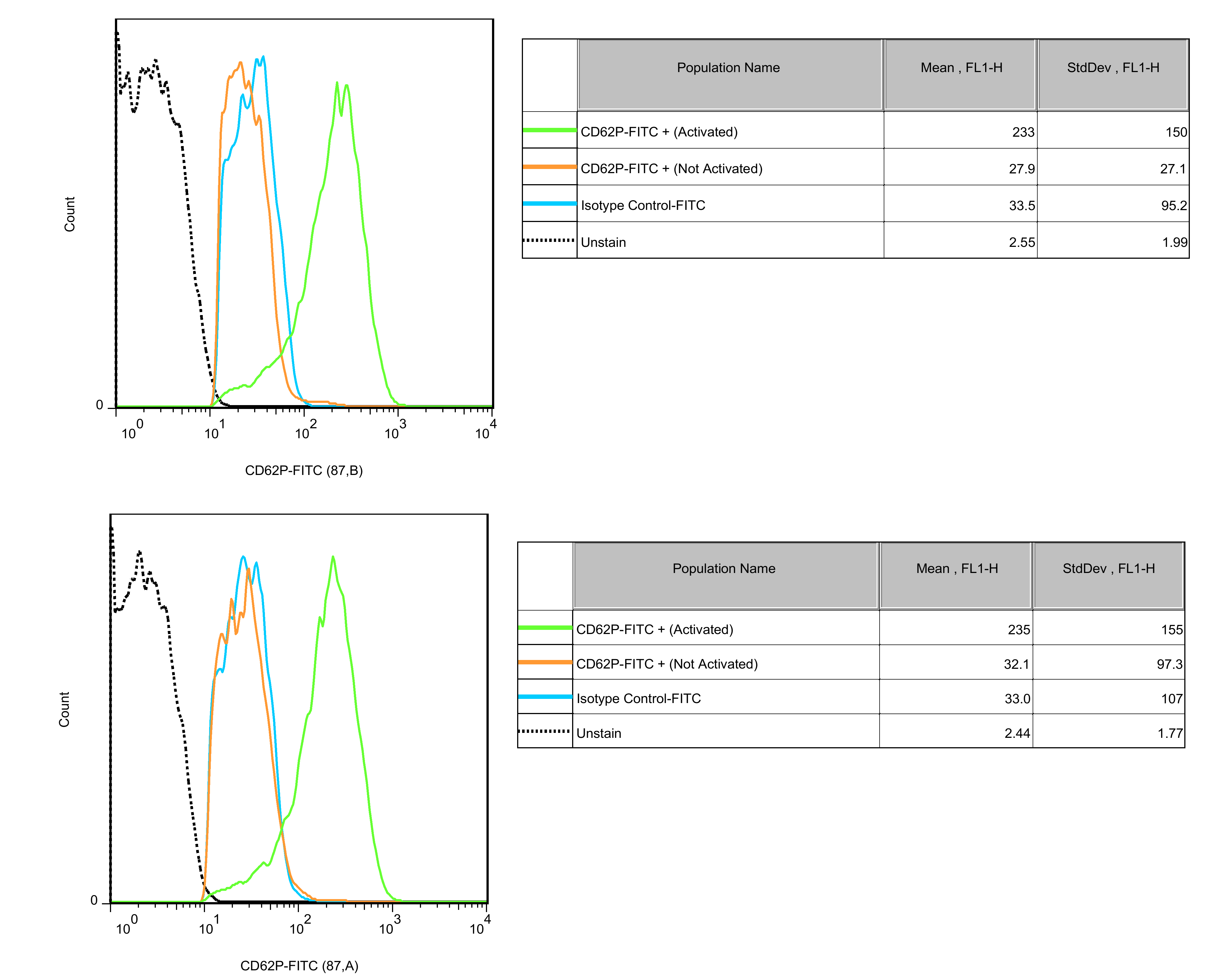

Supplement: Supplementary file 2 — Additional file 2: The Flow Cytometric assay results of per-patient levels of platelet CD62P Ag expression at baseline (B) and week 26 (A). [file 13098_2022_951_MOESM2_ESM.zip › 87.png]

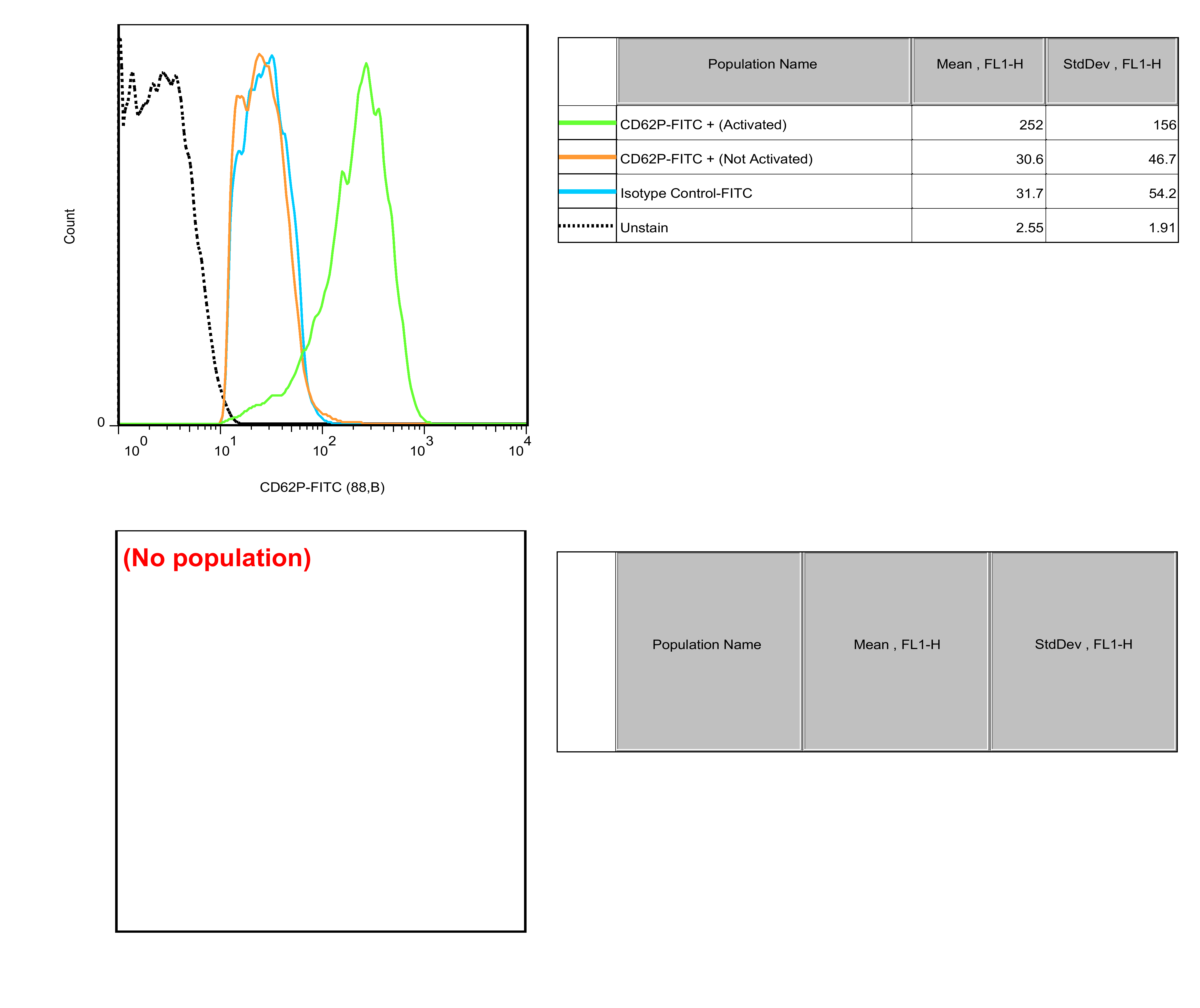

Supplement: Supplementary file 2 — Additional file 2: The Flow Cytometric assay results of per-patient levels of platelet CD62P Ag expression at baseline (B) and week 26 (A). [file 13098_2022_951_MOESM2_ESM.zip › 88.png]

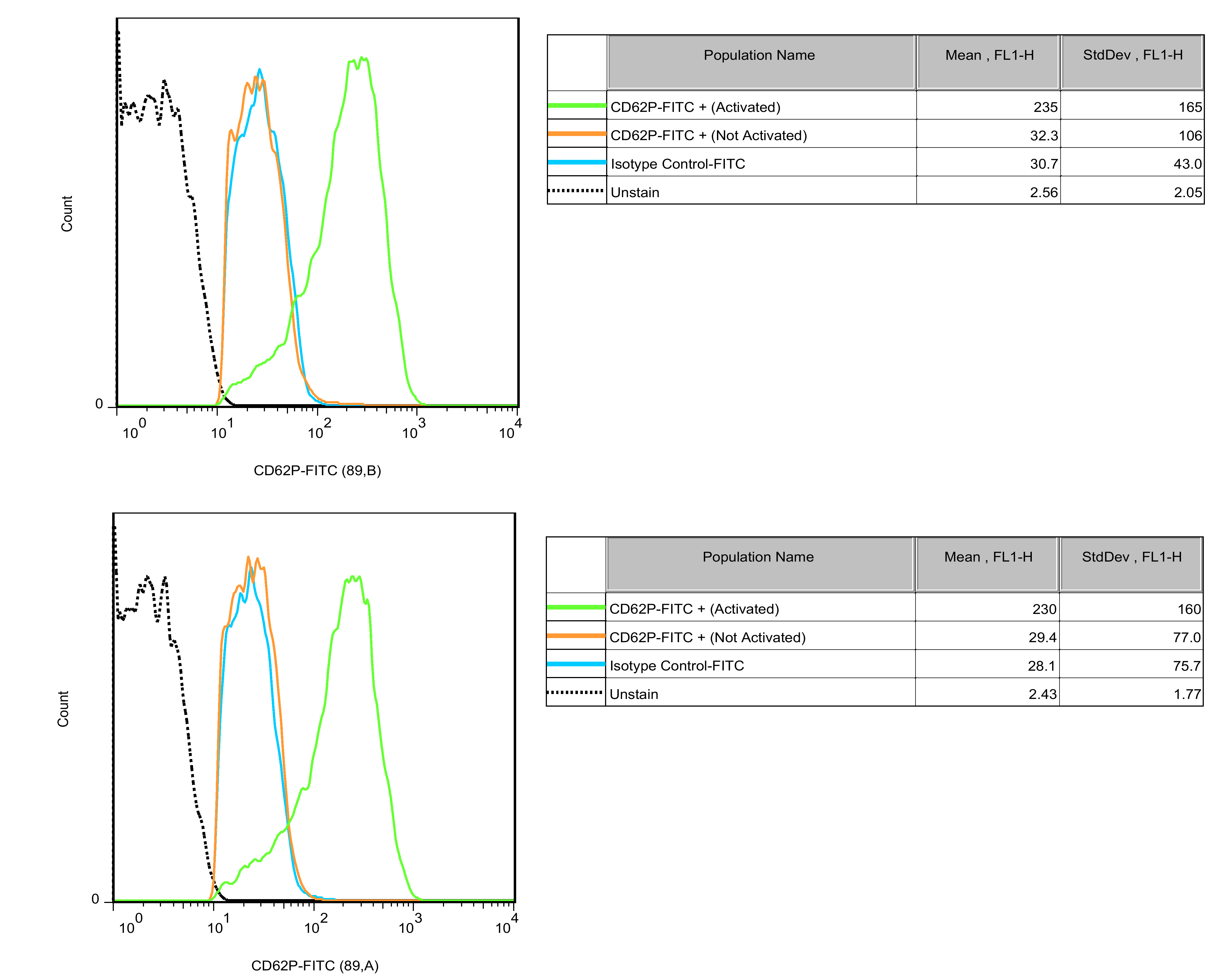

Supplement: Supplementary file 2 — Additional file 2: The Flow Cytometric assay results of per-patient levels of platelet CD62P Ag expression at baseline (B) and week 26 (A). [file 13098_2022_951_MOESM2_ESM.zip › 89.png]

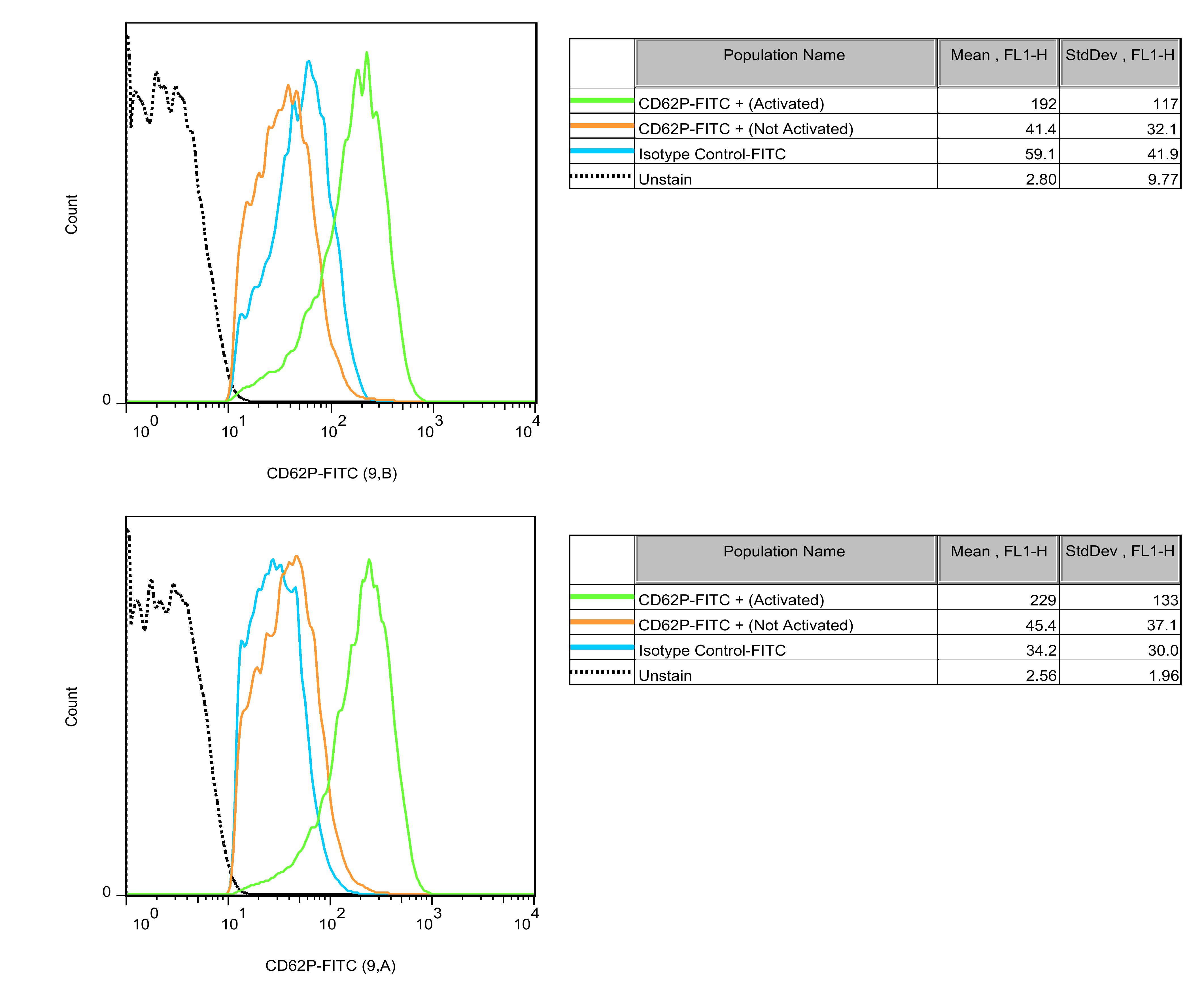

Supplement: Supplementary file 2 — Additional file 2: The Flow Cytometric assay results of per-patient levels of platelet CD62P Ag expression at baseline (B) and week 26 (A). [file 13098_2022_951_MOESM2_ESM.zip › 9.png]

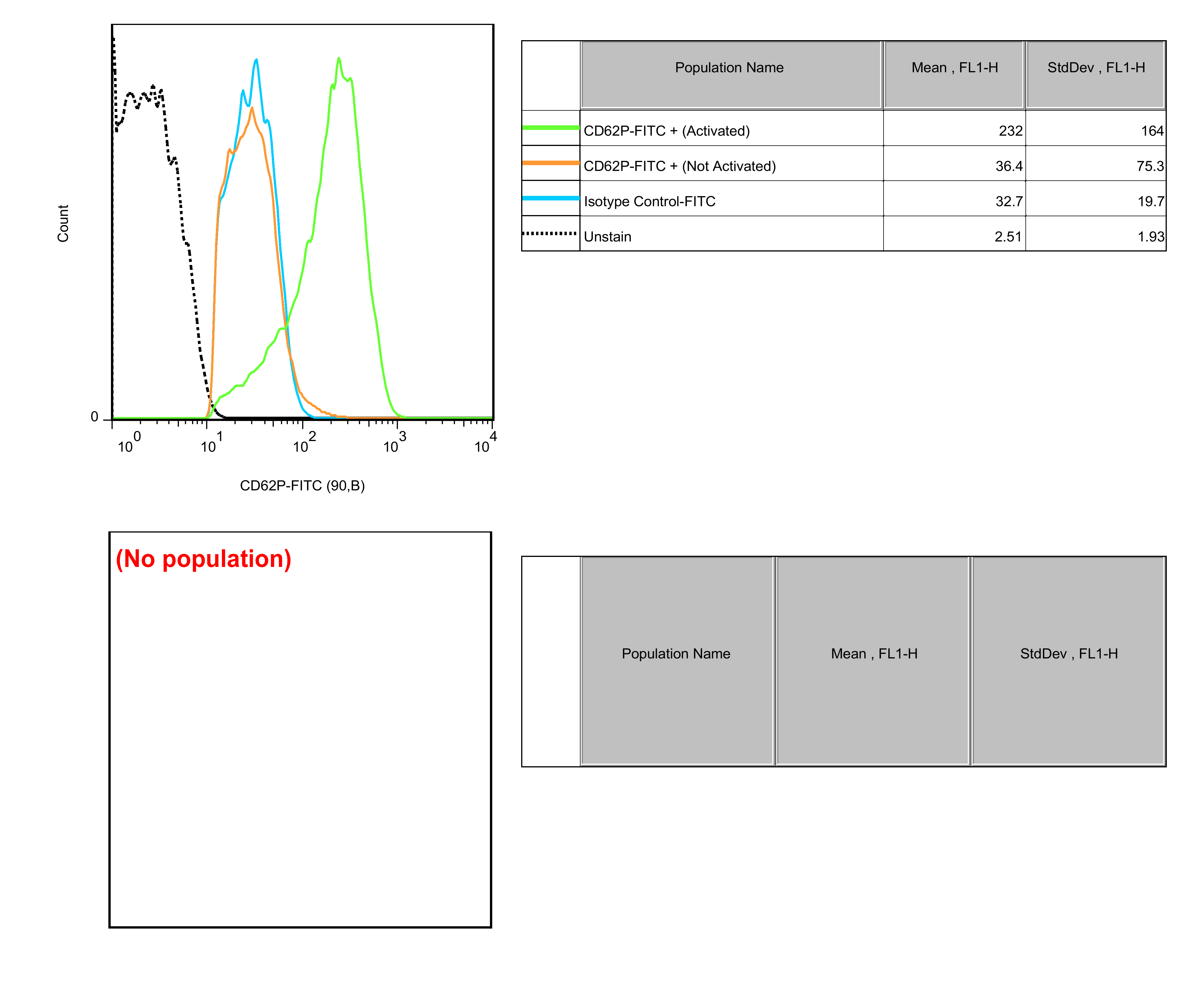

Supplement: Supplementary file 2 — Additional file 2: The Flow Cytometric assay results of per-patient levels of platelet CD62P Ag expression at baseline (B) and week 26 (A). [file 13098_2022_951_MOESM2_ESM.zip › 90.png]

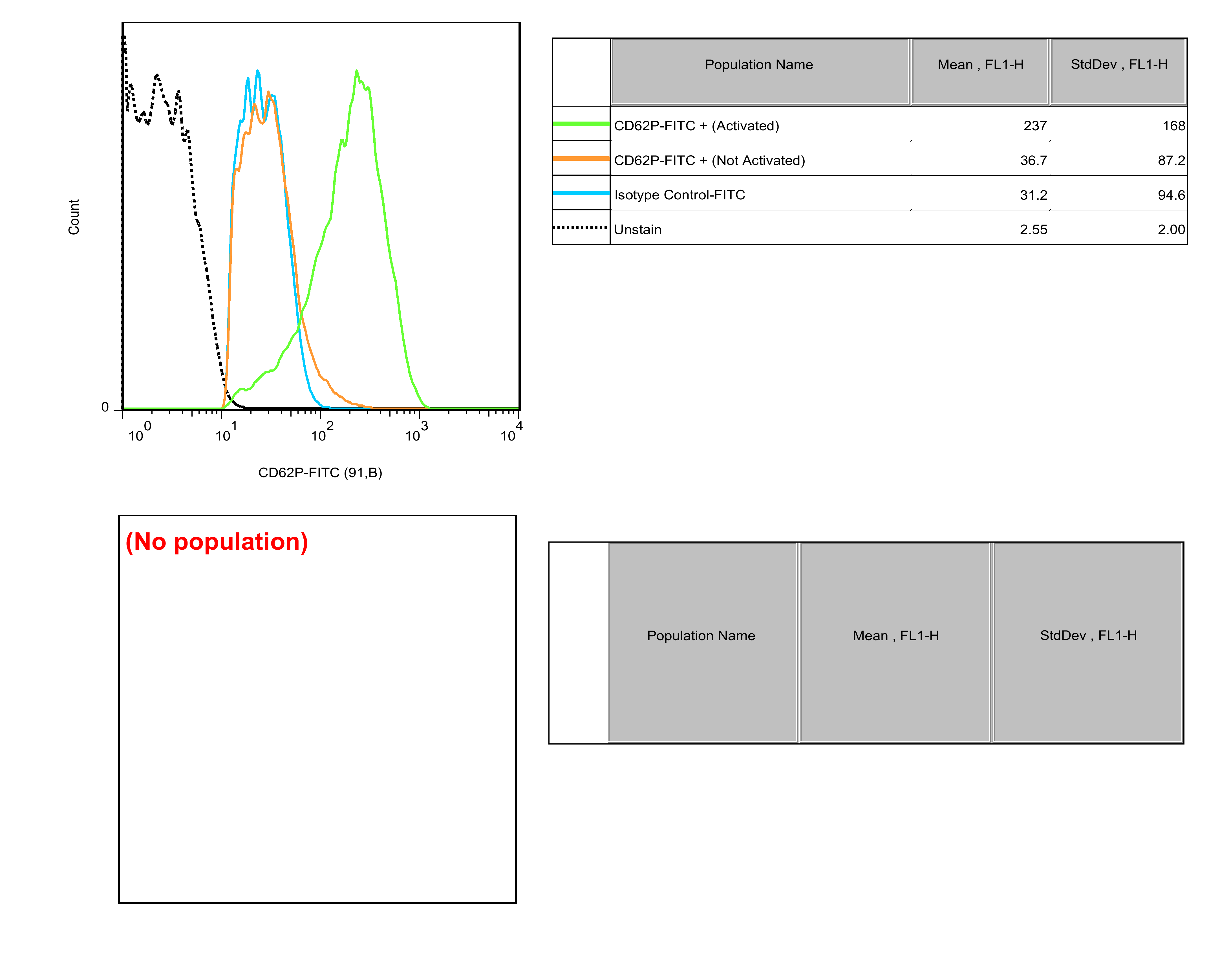

Supplement: Supplementary file 2 — Additional file 2: The Flow Cytometric assay results of per-patient levels of platelet CD62P Ag expression at baseline (B) and week 26 (A). [file 13098_2022_951_MOESM2_ESM.zip › 91.png]

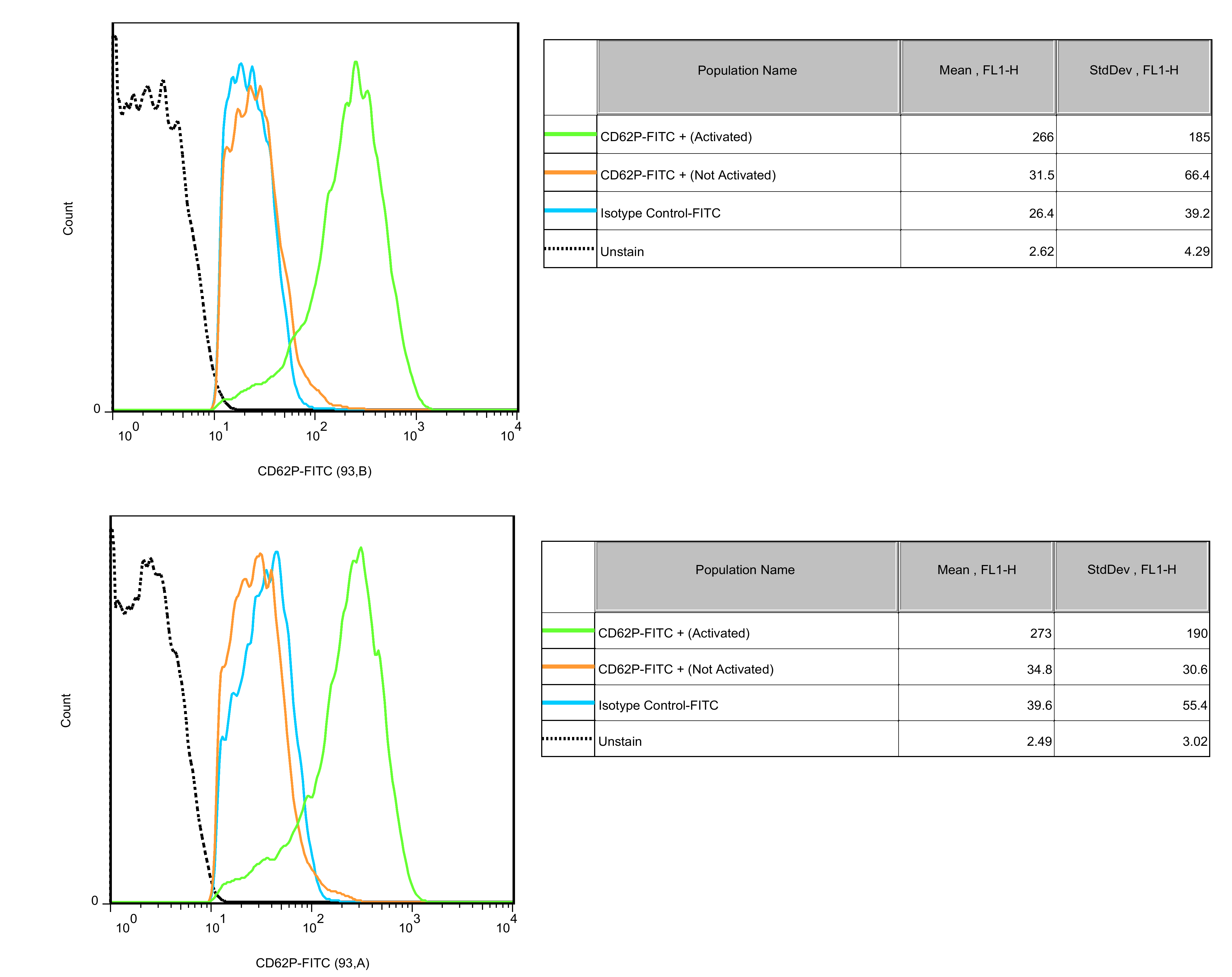

Supplement: Supplementary file 2 — Additional file 2: The Flow Cytometric assay results of per-patient levels of platelet CD62P Ag expression at baseline (B) and week 26 (A). [file 13098_2022_951_MOESM2_ESM.zip › 93.png]

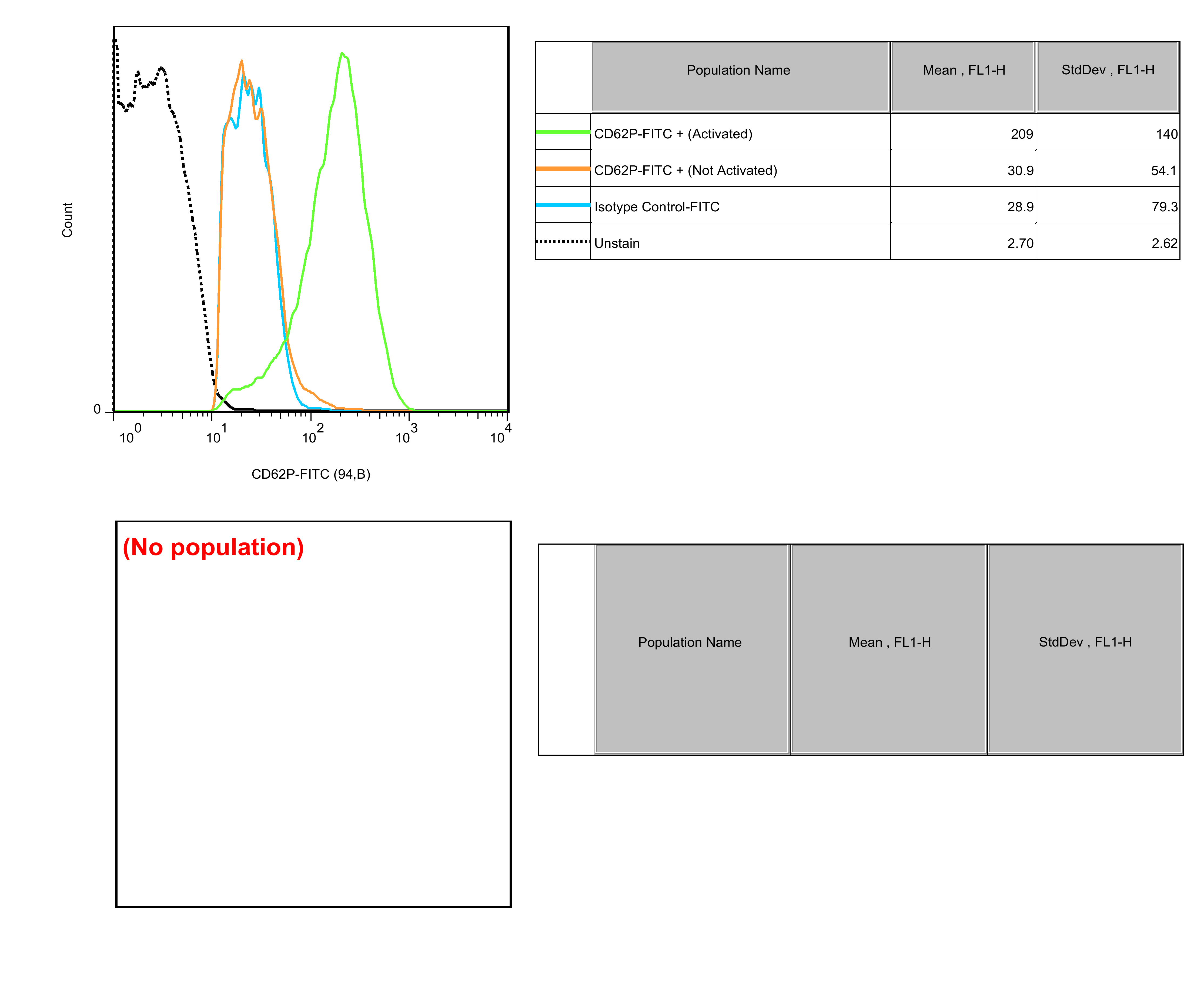

Supplement: Supplementary file 2 — Additional file 2: The Flow Cytometric assay results of per-patient levels of platelet CD62P Ag expression at baseline (B) and week 26 (A). [file 13098_2022_951_MOESM2_ESM.zip › 94.png]

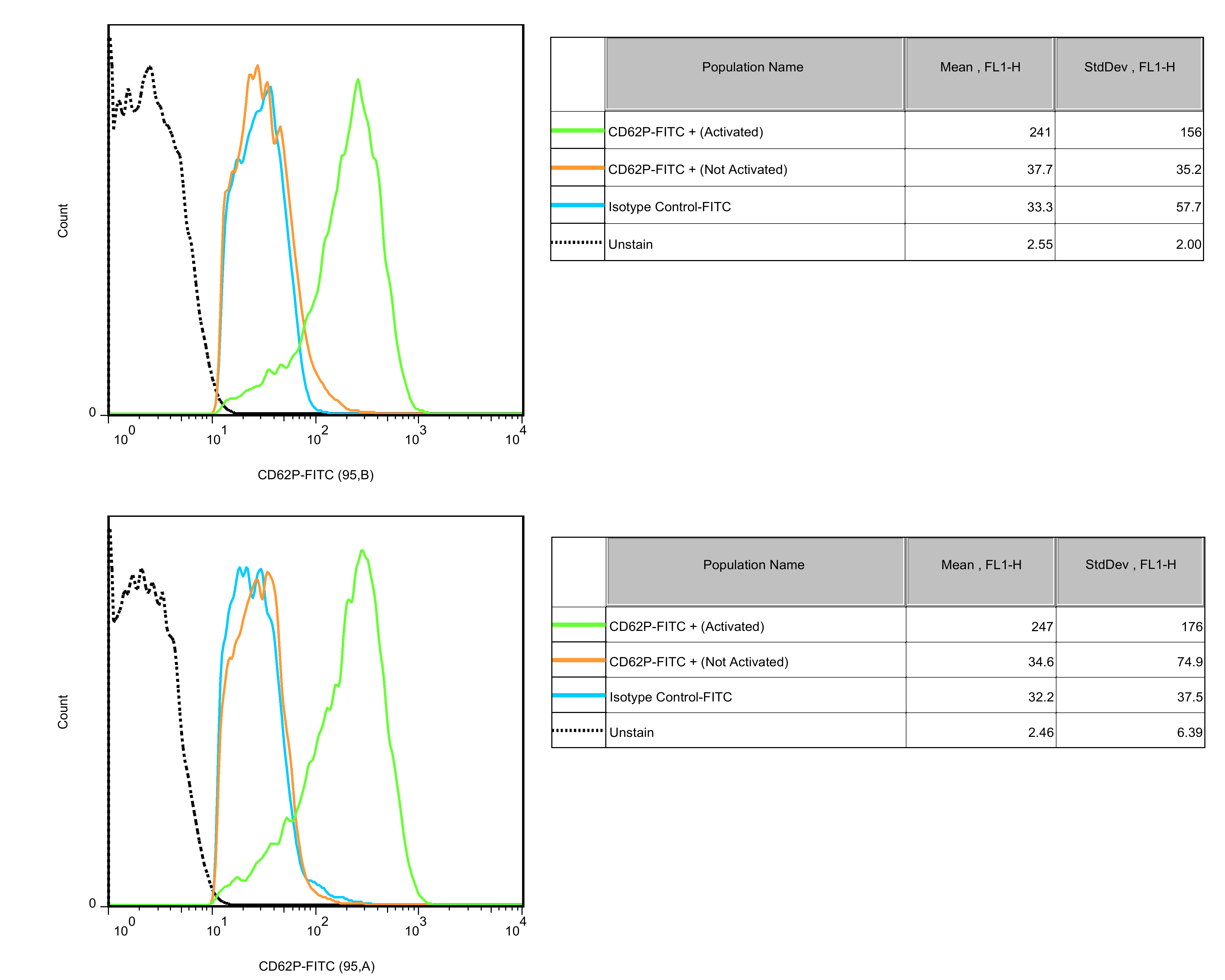

Supplement: Supplementary file 2 — Additional file 2: The Flow Cytometric assay results of per-patient levels of platelet CD62P Ag expression at baseline (B) and week 26 (A). [file 13098_2022_951_MOESM2_ESM.zip › 95.png]
